# European reference network for rare inherited congenital anomalies (ERNICA) evidence based guideline on the management of gastroschisis

**DOI:** 10.1186/s13023-024-03062-8

**Published:** 2024-02-12

**Authors:** Carmen Mesas Burgos, Willemijn Irvine, Alexandre Vivanti, Peter Conner, Egle Machtejeviene, Nina Peters, Joan Sabria, Ana Sanchez Torres, Costanza Tognon, Alberto Sgró, Antti Kouvisalo, Hester Langeveld-Benders, Rony Sfeir, Marc Miserez, Nils Qvist, Ausra Lokosiute-Urboniene, Katrin Zahn, Julia Brendel, Jordi Prat, Simon Eaton, Alexandra Benachi

**Affiliations:** 1 Department of Pediatric Surgery, Karolinska University Hospital, Eugeniavägen 23, C11:33, 17176 Stockholm, Sweden; 2 Department of Evidence Based Medicine and Methodology, Qualicura Healthcare Support Agency, Breda, The Netherlands; 3 Department of Obstetrics and Gynecology, Antoine Béclère Hospital, Paris Saclay University, Clamart, France; 4 Center for Maternal and Fetal Medicine, Karolinska University Hospital, Stockholm, Sweden; 5 Department of Gynecology, Hospital of Lithuanian University of Health Sciences Kaunas Clinics, Kaunas, Lithuania; 6 Department of Gynecology and Obstetrics, Erasmus MC, Rotterdam, The Netherlands; 7 Center for Maternal and Fetal Medicine, Hospital St Joan de Dieu, Barcelona, Spain; 8 Department of Neonatology, University Hospital La Paz, Madrid, Spain; 9 Department of Neonatology, University of Padua, Padua, Italy; 10 Department of Pediatric Surgery, University of Padua, Padua, Italy; 11 Department of Pediatric Surgery, Helsinki University Hospital, Helsinki, Finland; 12 Department of Pediatric Surgery, Erasmus MC, Rotterdam, The Netherlands; 13 Department of Pediatric Surgery, Centre Hospitalier Régional Universitaire de Lille, Lille, France; 14 Department of Surgery, UZ Leuven, Louvain, Belgium; 15 Department of Pediatric Surgery, Odense University Hospital, Odense, Denmark; 16 Department of Pediatric Surgery, Lithuanian University of Health Sciences Kauno Klinikos, Kaunas, Lithuania; 17 Department of Pediatric Surgery, Mannheim, Germany; 18 Department of Pediatric Surgery, Hannover Medical University, Hanover, Denmark; 19 Department of Pediatric Surgery, Hospital S Joan de Diu, Barcelona, Spain; 20 UCL Great Ormond Street Institute of Child Health, London, UK

**Keywords:** Gastroschisis, Abdominal wall defect, Silo

## Abstract

**Background:**

The European Reference Network for rare Inherited Congenital Anomalies, ERNICA, guidelines for gastroschisis cover perinatal period to help teams to improve care.

**Method:**

A systematic literature search including 136 publications was conducted. Research findings were assessed following the GRADE methodology. The evidence to decision framework was used to determine the strength and direction of recommendations.

**Results:**

The *mode or timing of delivery* do not impact neonatal mortality, risk of NEC or time on parenteral nutrition (PN). Intra or extra abdominal bowel dilatation predict complex gastroschisis and longer length of hospital stay but not increased perinatal mortality. Outcomes after *Bianchi procedure* and *primary fascia closure* under anesthesia are similar. *Sutureless* closure decreases the rate of surgical site infections and duration of ventilation compared to *surgical closure*. Silo-staged closure with or without *intubation* results in similar outcomes. Outcomes of *complex gastroschisis* (CG) undergoing early or delayed surgical repair are similar. Early *enteral feeds* starting within 14 days is associated with lower risk of surgical site infection.

**Recommendations:**

The panel suggests vaginal birth between 37 and 39 w in cases of uncomplicated gastroschisis. Bianchi’s approach is an option in simple gastroschisis. Sutureless closure is suggested when general anesthesia can be avoided, sutured closure. If anesthesia is required. Silo treatment without ventilation and general anesthesia can be considered. In CG with atresia primary intestinal repair can be attempted if the condition of patient and intestine allows. Enteral feeds for simple gastroschisis should start within 14 days.

**Supplementary Information:**

The online version contains supplementary material available at 10.1186/s13023-024-03062-8.

## Scope

Gastroschisis is an abdominal wall defect which occurs 3 per 10 000 live born and is predominantly (> 90%) diagnosed prenatally [1]. In fetuses with a gastroschisis, the bowel protrudes through a defect in the abdominal wall defect usually at the right of the umbilical cord. Although survival for liveborn infants with gastroschisis is more than 90% [2–6], the risk of an intrauterine fetal death (IUFD) is still 7.5 times higher than in the normal population and gastroschisis can be the cause of significant morbidity in the neonatal period.

The scientific literature suggests multiple options for every step along the care pathway of children with gastroschisis, both pre- and postnatally. The heterogeneity of practices, even within the same geographical area, warrants a careful evaluation of the evidence.

European Rare Disease Networks, such as ERNICA (European Reference Network for rare Inherited Congenital Anomalies) encourage healthcare teams from European countries to pool their data, compare their practice, challenge their protocols and write guidelines in order to improve the care of babies with rare diseases such as gastroschisis. Rare disease networks have also included parental/caregiver support groups in their multidisciplinary teams, and their experience and feedback are essential to the writing and implementations of guidelines.

The ERNICA guidelines for care of fetuses and babies with gastroschisis aims to answer 10 important questions covering the prenatal to early post-natal period in order to help teams and parents improve the quality of care of these babies, reduce unwanted heterogenic practice and highlight evidence gaps and promote research directions.

## Methodology

### Stakeholder involvement

A multidisciplinary guideline development group (GDG) was appointed to develop the guideline in January 2022. The members of GDG are primarily members of the abdominal wall defects working group within ERNICA. During the development of the guideline, the experts were divided in subgroups each working on a module. The PICO-questions, recommendations and considerations were created in joint meetings with most of the GDG members present. Details of all participating GDG members are displayed in Additional file 1: Appendix A. Unfortunately, the GDG and ERNICA did not succeed to recruit a patient representative for this project. The perspective of patients specific for gastroschisis care was included through a survey that was handed out to parents of patients of GDG members and one of the general patient representatives from ERNICA joined the working group session on outcome ratings.

### Implementation

The implementation of the guideline and the practical feasibility of the recommendations were taken into account during the different phases of guideline development. In doing so, explicit consideration was given to factors that could promote or hinder the implementation of the guideline in practice.

### Analysis of clinical care gaps

An initial group of experts interested in participating in the guideline was established after the third ERNICA annual meeting in Padua in April 2019. During the initiation phase, analysis and listing of clinical gaps was done through email discussions and google forms. The initial group was meeting in Rotterdam, December 2019, to finally discuss, select and agree to the most relevant topics that needed to be covered by the guideline.

### Questions and outcomes

**Fig. 1:**
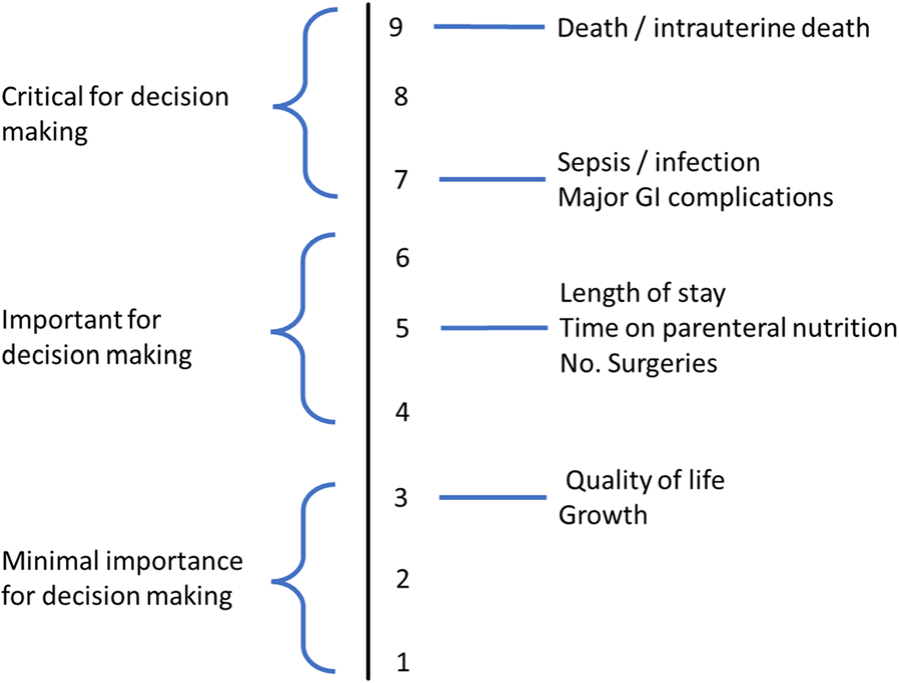
Outcome rating

The guideline was divided into 3 modules (prenatal care, management and closure of the abdominal wall and feeding) together covering 10 clinical questions. All questions were refined and structured according to the PICO framework. The GDG members pre-selected outcomes of interest to reflect the main concerns in gastroschisis care: mortality and morbidity. Specific outcomes were derived from the core-outcome set (COS) as published by Allin et al. [1] as the GDG considered this a valuable and balanced approach to effect evaluation for most gastroschisis topics and considered the approval of patients for this set an advantage. For some chapters, additional topic specific outcomes were included and, in every module, the evaluated outcomes are specified. During a working session with most of the GDG members and an ERNICA patient representative present, all considered outcomes were rated for their importance to clinical decision making and categorized as suggested by the GRADE system [2]. The outcome rating of the main outcomes used for evaluation in this guideline is displayed in Fig. 1.

### Literature search and selection of literature

A systematic literature search was conducted by a professional biomedical information specialist in January 2022 to identify all available literature on gastroschisis and synonyms. The search was conducted in Embase and Medline (all). The full search strategy is available in the supplementary material (Additional file 1: Appendix A). reported in the supplementary material. The chairs and methodologist screened all results (n = 394) based on title and abstract and excluded the following:

- Original studies with < 10 included patients
- Articles published before 2010
- Case reports
- Expert opinion
- Letters to the editor
- Editorials
- Reviews

**Fig. 2:**
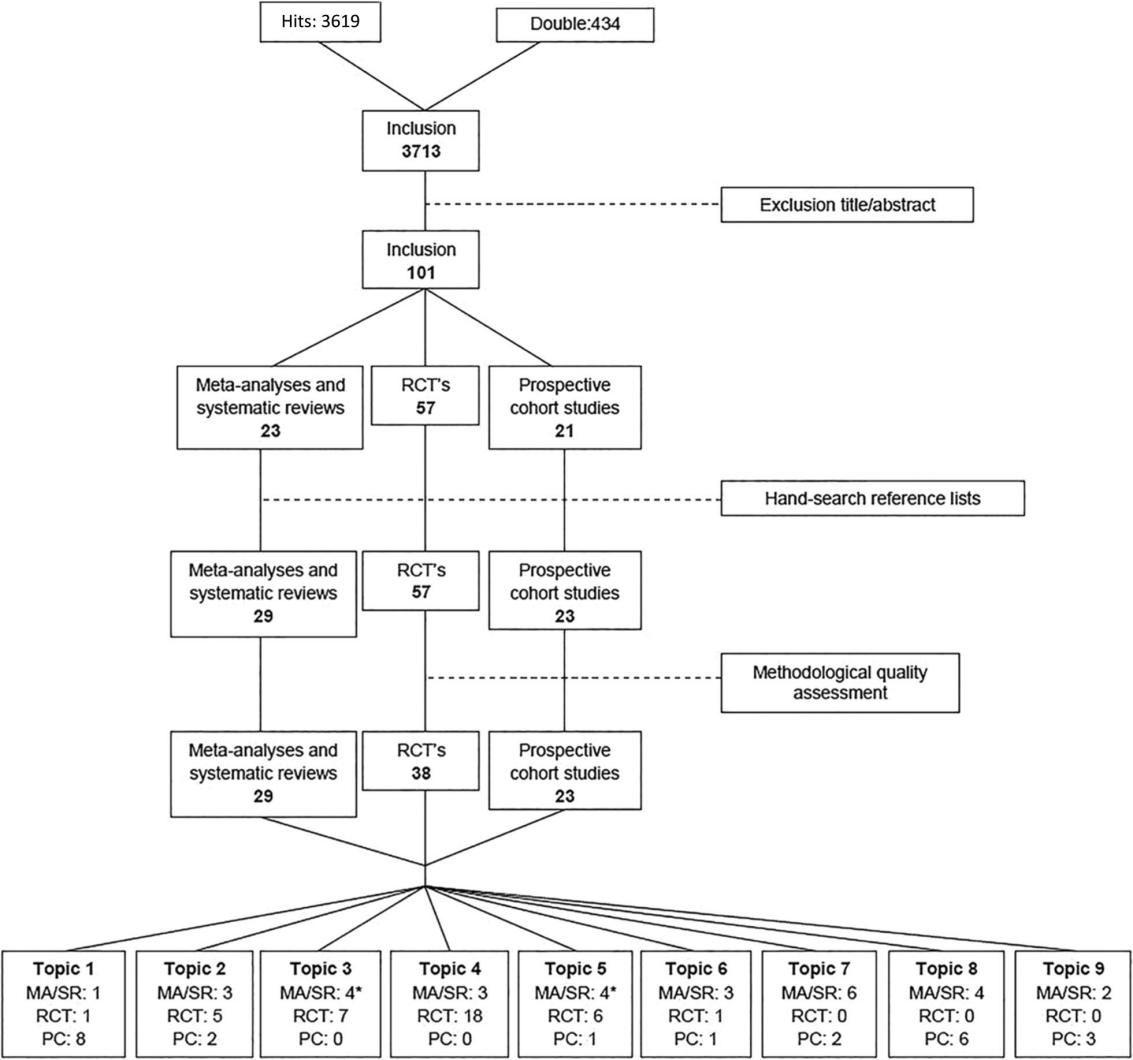
PRISMA flowchart

Although the Guideline is intended for European use, no geographical restrictions were applied, provided that the level of care was comparable to European Countries. After screening based on title and abstract, all included publications (n = 136) were included for full-text review and labelled to one of the modules according to their research topic (Fig. 2). A package of electronic full text papers was distributed to each subgroup for further evaluation of eligibility.

### Summary of findings

Research findings were summarized per outcome. Based on the ‘Grading Recommendations Assessment, Development and Evaluation (GRADE)’ methodology, all of the evidence on one outcome of interest, was collectively assessed as a ‘body of knowledge’ to determine the quality of evidence for that outcome (Table 1).

**Table 1:** Grade

Grade	Definition
High	We are very confident that the true effect lies close to that of the estimate of the effect
Moderate	We are moderately confident in the effect estimate: The true effect is likely to be close to the estimate of the effect, but there is a possibility that it is substantially different
Low	Our confidence in the effect estimate is limited: The true effect may be substantially different from the estimate of the effect
Very Low	We have very little confidence in the effect estimate: The true effect is likely to be substantially different from the estimate of effect

All conclusions were formulated in such a way that the strength of the evidence was reflected in the wording of the conclusion. The following wording was chosen:

- GRADE high: It has been shown…
- GRADE moderate: It is likely…
- GRADE low: There are indications…
- GRADE very low: There are cautious indications…

Justifications and GRADE profiles are available in the supplementary materials (Additional file 2: Appendix B).

### Considerations and recommendations

To determine the strength and direction of a recommendation, the evidence to decision framework was used. The purpose of Evidence to Decision (EtD) frameworks is to help people use evidence in a structured and transparent way to inform decisions in the context of clinical recommendations and other health system or public health decisions [3].

The framework was used as a guide to structure a two days consensus meeting with all members of the GDG where considerations, recommendations and research needs following the literature analysis were discussed, including consideration of articles, such as expert opinion articles and studies with < 10 patients, that did not meet the inclusion criteria. To prepare for this meeting, a summary of findings of all modules was distributed amongst GDG members accompanied by an electronic survey covering all the aspects of the evidence to decision framework (Panel Voice). The survey allowed panel members to collaboratively assess and develop judgements on the available evidence and additional considerations for each module. The survey included comment boxes after each question to invite GDG members to provide discussion points and rationale for their votes before the discussion.

The evidence to decision framework led to a collective agreement on the type of recommendations for all chapters. In accordance with the GRADE method, a low probative value of conclusions in the systematic literature analysis does not exclude a strong recommendation in advance, and weak recommendations are also possible with a high probative value. The GDG decided between five types of recommendations and chose uniform wording of recommendations for each of them as displayed in Table 2. All main outcomes of the EtD framework and recommendations are included in this executive summary, full EtD tables are available in the supplementary materials (Additional file 3: Appendix C).

**Table 2:** Type of recomendation

Type of recommendation	Wording
Strong recommendations against the intervention	The panel recommends against / to refrain from
Conditional recommendation against the intervention	The panel suggests against / to refrain from
Conditional recommendation for either the intervention or the comparison	The panel suggests either … or …
Conditional recommendation for the intervention	The panel suggests …
Strong recommendation for the intervention	The panel recommends …

### Organization of care

A fourth module was devoted to topics related to organisation of care. For this module, a less systematic but more pragmatic approach was used where evidence and expert opinion were combined to create recommendations during group discussions based on the principles of a consensus development conference [4].

## Results

A total of 32 studies were found eligible to include for one of the health questions. The GDG developed 23 recommendations. An overview of the clinical questions and their recommendations is provided in Table 3. Evidence, considerations and justification for each recommendation is summarized below. A complete version of each module is available at the ERNICA website.

**Table 3:** Recommendations

Module 1—Prenatal care	
1.1 a	The panel suggests birth between 37^+0^ and 39^+0^ weeks in children with uncomplicated gastroschisis
1.1 b	The panel suggests vaginal birth in children with uncomplicated gastroschisis
1.1	The panel suggests vaginal birth between 37^+0^ and 39^+0^ in children with uncomplicated gastroschisis
1.2	The panel suggests interpreting Intra-Abdominal Bowel Dilatation and Extra-Abdominal Bowel Dilatation on follow up ultrasound as predictors for complex gastroschisis
	The panel recommends evaluating the fetus with gastroschisis based on a complete image of different ultrasound parameters combined. It would, therefore, be useful to evaluate bowel thickness, gastric dilation, herniation of the stomach and/or bladder through the abdominal wall defect, presence of polyhydramnios, fetal growth parameters, fetal movement and size of the abdominal wall defect in addition to IABD and EABD
	There is insufficient evidence or expert experience to suggest using altered mesenteric artery flow as a prognostic factor for a complex gastroschisis. The experts do not formulate any recommendation
Module 2—Management and closure of the abdominal wall defect	
2.1	The panel suggests considering Bianchi’s approach as a possible option for treatment of neonates with simple gastroschisis and good bowel conditions
2.2	The panel suggests sutureless closure in neonates with gastroschisis and who undergo repair without general anesthesia (silo-staged or primary)
	If primary closure under general anesthesia is performed, the panel suggests a sutured closure to avoid possible hernia development (and surgery) later in life
2.3	The panel suggests primary intestinal repair for complex gastroschisis patients with atresia if the general condition and bowel allow for primary intestinal repair
	The panel suggests determining the treatment strategy for patients with complex gastroschisis based on the individual characteristics, general condition, and bowel condition of each patient
2.4	The panel suggests considering treatment without ventilation and general anesthesia as an option in case of patients with simple gastroschisis and a stable condition if staged closure is the treatment option of choice
	The panel recommends close monitoring of comfort and pain using objective measurements in patients undergoing staged closure while breathing spontaneously
2.5	The panel suggests using either synthetic or biologic mesh in cases where fascial closure is not feasible after silo reduction The final decision for the type of mesh should be based on inhouse expertis of the pediatric surgical team
Module 3—Feeding	
3.1a	The panel suggests starting enteral feeding within the first 14 days post repair. If the neonate’s condition is favorable with low aspirates, starting enteral feeds before the 7th day can be considered
3.1b	The panel suggests the implementation of a feeding protocol in centers to start enteral feeding after gastroschisis correction in neonates
3.2	The panel suggests the upper extremity as first choice in case of placement of a peripherally-inserted central catheter The risk difference is larger in patients with a SILO. Should lower extremity PICC line be the option of choice in a patient with a SILO, cautious surveillance for complications is warranted
Module 4—Organization of care	
4.1	Most parents were satisfied with the quality of care but suggestions for improvement were to obtain psychological support at all time, to provide more information in written folders, parents support groups, in the NICU: to allow relatives, adjusted sedation/analgesia protocols during reduction of bowels and structured and early feeding protocols
4.2	Decisions regarding antenatal follow-up, the timing, place and mode of delivery, as well as the procedures to be performed during the neonatal period should be discussed by the medical team prior to consultation with the parents to ensure that the information given is consistent and that the dialogue is homogeneous
4.3	A multidisciplinary care team for gastroschisis should include a maternal–fetal specialist, obstetrician, neonatologist, pediatric surgeon, pediatric anesthetist, pediatric gastroenterologist, dietitian, social worker and/or psychologist and coordinating nurse
4.4	There are indications that higher volume centers have a significantly lower mortality rate compared to lower volume centers
4.5	Measurement of QoL using a validated instrument is important The guideline development group endorses the recommendation by Allin et al.(2019), to use the PedsQL until a gastroschisis-specific QoL instrument is developed and validated

### Evidence, justifications and considerations

#### Module 1: Prenatal care

1.1. Delivery

1.1a. Timing of delivery: Is preterm delivery as compared to term delivery indicated in fetuses with gastroschisis?

One meta-analysis [5] could be included that covered the outcomes mortality, sepsis, severe gastro-intestinal complications and time on parental nutrition. Three observational studies in group G1 investigated the effect of planning a full-term birth versus an elective preterm birth (before 37 weeks). In total, 153 patients were born at term and 100 patients had an elective preterm birth. The results suggest that an elective preterm delivery birth is associated to a reduced risk of sepsis (OR 0.43 (95 CI 0.24–0.78); *p* = 0.006). Other outcomes did not yield significant differences between groups.

The following conclusions were drawn:

**Table:** 

	Grade
There are cautious indications that time of delivery does not impact neonatal mortality among fetuses with gastroschisis [5]	Very low
There are indications that an elective preterm delivery is associated to a decrease in the risk of neonatal sepsis among fetuses with gastroschisis [5]	Low
There are cautious indications that time of delivery may not impact the risk of necrotizing enterocolitis after birth among fetuses with gastroschisis [5]	Very low
There are cautious indications that time of delivery may not impact the time on parenteral nutrition after birth among fetuses with gastroschisis [5]	Very low

Overall, the evidence of certainty is low and this gives the panel too little indications that preterm birth has any advantages and we do have indications that preterm birth has possible neurodevelopmental disadvantages. Therefore, the panel sees no basis to recommend or suggest the invasive option of preterm delivery. The evidence on a decrease of the risk for sepsis is too uncertain to recommend an active intervention, however, we don’t have any information about babies with complications or suspected complex gastroschisis. The panel feels that even if there is no evidence, it feels uncomfortable to let the pregnancies continue further than 39 weeks because of the increase in risks for intra uterine fetal demise.

1.1b. Mode of delivery: Is delivery by cesarean section, as compared to vaginal delivery, indicated in fetuses with gastroschisis?

One meta-analysis was included that provided information on the outcomes: mortality, sepsis, necrotizing enterocolitis and time on parenteral nutrition. The meta-analysis by Kirollos et al. [6] included 38 observational studies an representing a total of 6577 patients with gastroschisis of whom 3019 (46%) were born vaginally and 3558 (54%) by caesarean delivery. The results did not yield significant differences for any of the outcomes. The following conclusions were drawn.

**Table:** 

	Grade
There are cautious indications that mode of delivery does not seem to impact neonatal mortality among fetuses with gastroschisis [6]	Very low
There are cautious indications that mode of delivery does not seem to impact the risk of neonatal sepsis among fetuses with gastroschisis [6]	Very low
There are cautious indications that mode of delivery does not seem to impact the risk of necrotizing enterocolitis after birth among fetuses with gastroschisis [6]	Very low
There are cautious indications that mode of delivery does not seem to impact the time on parenteral nutrition after birth among fetuses with gastroschisis [6]	Very low

The meta-analysis only included observational studies. Evidence synthesis showed a high risk of bias which resulted in the need to downgrade the level of certainty to very low for all outcomes. Although the quality of the evidence is very low, the panel considers vaginal delivery to be a safe option for children with uncomplicated gastroschisis and there is no evidence that suggests advantages of delivery via cesarean section. According to the WHO, cesarean sections should ideally only be undertaken when medically necessary [7]. As additional consideration the panel mentioned that avoiding a c-section may have a positive effect on the mother.

1.2 Prenatal ultrasound

1.2. Can ultrasonographic findings in fetuses with gastroschisis predict perinatal outcome?

Prenatal ultrasound markers for prediction of adverse outcome were mainly evaluated by a recent systematic reviews and meta-analysis from 2021 [8]. A systematic review and meta-analysis from 2015 [9] as well as recent observational studies were used as supportive evidence. With this data, the panel evaluated the value of intra-abdominal bowel dilatation (IABD), extra-abdominal bowel dilatation (EABD) and mesenteric artery flows as prognostic factors for mortality and morbidity.

#### IABD

The association between IABD and intrauterine death was only investigated by D’Antonio et al. [9]. They reviewed 6 studies (331 fetuses) with gastroschisis and found no significant association between IABD and intra-uterine death. Both meta-analysis [8, 9] looked into the association between IABD and neonatal death but neither found a significant relation. IABD was found to be associated with higher odds of complex gastroschisis [8–10]. Sun et al. reviewed 13 studies (1083 participants) with IABD. The risk of complex gastroschisis was higher in fetuses with IABD (OR = 5.42; 95% CI 3.24 to 9.06; *p* < 0.001; and low heterogeneity: I2 = 33%). compared with non-IABD fetuses. Second trimester IABD have greater specificity for the prediction of complex gastroschisis than third trimester values with specificity of 95.6% (95% CI 58,1—99,7) [8]. Notably, bowel dilatation ≥ 10 mm in the second trimester had the highest specificity (96%). The results of this meta-analysis also indicated that IABD is associated with longer hospital stay [8].

#### EABD

The analysis for EABD and neonatal death either yielded no significant results in both reviews [8, 9].

EABD on prenatal ultrasound was found to be associated with higher odds of complex gastroschisis after birth [8–10]. The association between EABD and complex gastroschisis was also described by Sun et al. [8]. They reviewed 14 studies (1439 participants) with EABD. The risk of complex gastroschisis was higher in fetuses with EABD (OR = 2.27; 95% CI 1.40 to 3.66; *p* < 0.001) compared with non-EABD fetuses. Second versus third trimester analysis showed that only EABD detected in the third trimester was significantly associated with higher odds of complex gastroschisis (OR = 2.05; 95% CI 1.23 to 3.42; *p* = 0.006).

### Mesenteric artery flow

There were some indications of very low quality of evidence that perturbed mesenteric artery flows are associated with higher mortality. The evidence on the importance of mesenteric artery flows was of very low quality but mainly due to lacking of a definition of ‘perturbed’ [11, 12]. The following conclusions were drawn:

**Table:** 

	Grade
It is likely that there is a significant association between IABD (especially second trimester) and complex gastroschisis [8]	Moderate
It is likely that there is a significant association between IABD and longer length of hospital stay [8]	Moderate
There are indications there is no significant association between IABD and intrauterine fetal demise or postnatal mortality [8]	Low
There are cautious indications there is no significant association between IABD and duration of TPN [8]	Very low
It is likely that there is a significant association between EABD (especially third trimester) and complex gastroschisis [8]	Moderate
It is likely there is no significant association between EABD and intrauterine fetal demise or postnatal mortality [8]	Moderate
There are cautious indications there is no significant association between EABD and longer hospital stay [8]	Very low
There are cautious indications there is no significant association between EABD and duration of TPN [9]	Very low
There are cautious indications that an association between higher mortality and perturbed mesentery artery flows exists. The definition of ‘perturbed’ is unclear[11, 12]	Very low

The harms of using these ultrasound parameters are trivial and we are moderately confident that IABD and EABD are predictors for complex gastroschisis. For very large dilation there are even higher concerns for complex gastroschisis although no clear cut-off could be defined from the analyzed literature. Prenatal identification of complex gastroschisis would improve parental counseling and perinatal planning. No significant harms associated with more frequent ultrasound monitoring were found in the evidence or came to discussion. Therefore, it seems beneficial to recommend the use of IABD and EABD as prognostic factors by including these measurements in prenatal evaluation. In this chapter only a preselected, prioritized group of ultrasound markers were reviewed, but the panel believes it remains important to consider the whole picture.

### Module 2: management and closure of the abdominal wall

#### Bianchi’s approach or primary surgical reduction

2.1. Is attempt to primary surgical reduction under general anesthesia (silo as rescue), compared to cot-side reduction and closure (Bianchi’s approach)​ associated with better outcome in neonates with simple gastroschisis?

Observational data was found on the outcomes: mortality, sepsis, bowel ischemia (severe gastrointestinal complications), time on parenteral nutrition(PN) and length of stay [13–17]. None of these studies yielded significant differences for these outcomes between the two interventions. The following conclusions were drawn:

**Table:** 

	Grade
There are cautious indications that the risk of mortality is comparable in patients who undergo Bianchi procedure compared with primary surgical reduction with silo as rescue under general anesthesia [13, 15–17]	Very low
There are cautious indications that the incidence of septicemia after Bianchi procedure is comparable with primary surgical reduction with silo as rescue under general anesthesia [14]	Very low
There are cautious indications that the risk of bowel ischemia in the Bianchi procedure is comparable with primary surgical reduction with silo as rescue under general anesthesia [13, 16, 17]	Very low
There are cautious indications that the risk of PN exceeding 60 days of duration, and the mean number of days on TPN in Bianchi procedure is comparable to patients who undergo surgical reduction with silo as rescue under general anesthesia [13, 17]	Very low
There are cautious indications that length of hospital stay is comparable in patients who undergo Bianchi procedure compared with primary surgical reduction with silo as rescue under general anesthesia [13, 14, 16, 17]	Very low

Because all evidence comes from retrospective observational studies, confounding and selection bias result in the need for careful interpretations of conclusions. Professionals on the guideline panel agree that Bianchi’s approach is not suitable as universal choice for closure of gastroschisis but can be safe and effective in selected patients with favorable anatomy and good general condition. An additional consideration was the possible avoidance of general anesthesia in Bianchi’s approach. As there are indications for neurotoxic effects of general anesthetics in neonates, avoiding anesthesia could be beneficial. Another consideration includes the evaluated outcomes. The importance of the main outcomes is clear to all stakeholders. However, patients might place higher value on the long-term cosmetic results and for parents, comfort and pain in the neonate during treatment is important. Neither outcome was evaluated in the current available studies. The panel believes that the cosmetic results are better in Bianchi’s approach combined with sutureless closure and that it could be beneficial for the neonate to avoid general anesthesia and intubation.

2.2. Sutureless closure

2.2. What are the (un)favorable effects of surgical closure under general anesthesia compared to cot-side plastic closure in neonates with gastroschisis who undergo staged closure (silo application)?

Two systematic reviews and meta-analysis could be included for the analysis of this chapter [18–20] A later published, larger observational study was used as supportive evidence or for outcomes that were not included in the meta-analyses [20]. Data was found on the outcomes: mortality, surgical site/wound infection, duration of ventilation, length of stay and time on parenteral nutrition. In the meta-analysis of Miyake et al. [18] 18 patients undergoing plastic closure and 18 undergoing surgical closure were compared. In their analysis, plastic closure was associated with a significantly decreased length of stay when compared to surgical closure (MD -14.06, CI -22.86,-5.26, *p* = 0.002). Fraser et al. [20], analysing 242 patients (30 plastic closure, 212 surgical closure), didn’t find a statistically significant difference in length of stay between methods. No evidence suggesting a significant risk differences for mortality and time on parenteral nutrition was found. Evidence suggested that sutureless closure has advantages concerning the risk of wound infection (RR 0.58 [95%CI 0.36–0.96]) [18, 19] and the duration of ventilation (MD -5.76 days) [18, 20]. The following conclusions were drawn:

**Table:** 

	Grade
There are cautious indications that there is no significant difference in mortality between sutureless plastic closure and surgical closure after silo staged repair for gastroschisis [18]	Very low
There are cautious indications that sutureless plastic closure significantly decreases the rate of surgical site/wound infections after silo staged repair for gastroschisis when compared to surgical closure [18–20]	Very low
There are cautious indications that sutureless plastic closure significantly decreases the duration of ventilations after silo staged repair for gastroschisis when compared to surgical closure [18, 20]	Very low
It is unclear if method of closure after silo staged repair for gastroschisis affects the length of stay [18, 20]	Very low
There are cautious indications that there is no significant difference in PN duration between sutureless plastic closure and surgical closure after silo staged repair for gastroschisis [20]	Very low

In this analysis sutureless plastic closure of the abdominal wall defect seems to be favorable in terms of infection rate and duration of ventilation and hospital stay. Ventilation and hospital stay may be affected by several events and factors and evidence is too scarce or conflicting to give a real recommendation against or for a specific closure technique based on the evaluation of these outcomes. The wound/surgical infection rate could be strictly related to the closure technique and the reported evidence would lead to the recommendation for a sutureless plastic closure when the patient's conditions and abdominal tension are appropriate. The avoidance of general anesthesia is an additional benefit.

### Complex gastroschisis

2.3 What are the (un)favorable effects of immediate surgery (resection with primary anastomosis or creation of enterostomy) compared to primary reduction and delayed surgery, in patients with complex gastroschisis?

After literature research the panel confirmed the definition of complex gastroschisis (CGS) as gastroschisis complicated by congenital intestinal atresia, necrosis, perforation or volvulus. All these features can be associated. The only data on risk differences for our outcomes of interest between early (< 21 days of life) and late surgery were described by Alshehri et al. [21] in a prospective national study, with 78 CGS patients. Their study suggests that early treatment (< 21days of life) is not associated with increased complications but allows patients to receive and tolerate enteral feeding earlier. Alsheri et al. [21] described the number of patients exclusively fed by TPN at 28 days of life. In this sample, patients who had undergone early surgery, were less likely to be depending on TPN at 28 days of life (RR 0.41, 95% CI, 0.17 to 1.02, *p* = 0.06) but it did not reach significance. There was however significant difference in age at the first enteral feed (14.8 ± 2.6 days versus 44.7 ± 7.4 days, *p* = 0.002). This indicates that patients who undergo early surgery receive and tolerate enteral feeding earlier [21].

**Table:** 

	Grade
There are cautious indications that there are no risk differences in mortality, infectious complications or the length of hospital stay in patients with complex gastroschisis who had early versus delayed surgical treatment [21]	Very low
There are cautious indications that patients with complex gastroschisis receive and tolerate enteral feeds earlier if they have early surgical treatment [21]	Very low

The available evidence is graded as very low. Low event numbers and small sample sizes may have caused imprecision and made it difficult to reach significance for some of the outcomes. Because the evidence was so scarce, the panel considered published opinions of other experts on this topic. Bhat et al. [22] suggest that the surgical approach of intestinal atresia must be individualized based on gestational age, birth weight, clinical status, and the condition of the bowel. In good condition, intestinal continuity restoration can be done together with primary closure. Other experts support this approach by [23, 24]proposing that a universal algorithm for these patients is not relevant because of the heterogeneous appearances of these cases. The panel agrees that the therapeutic strategy should be well designed in these patients based on the general condition of the patient and the bowel. However, the results from our literature analysis indicate that early establishment of intestinal continuity seems a safe option and does not lead to an increased complication risk in selected patients with gastroschisis and atresia.

2.4. Intubation and ventilation

2.4 What are the (un) favorable effects of spontaneous breathing during Silo reduction vs intubation and mechanical ventilation in neonates with gastroschisis?

After a distinct search for the best available evidence, only two studies with indirect evidence on silo treatment with and without ventilation could be included [25, 26]. In a retrospective cohort published by Hong et al. [26] they reported 17 non-complicated gastroschisis patients with a preformed silo reduction. In all patients, silo placement was performed as bed-side procedure, without general anesthesia. Looking at the ventilation days, 5 infants were never ventilated compared to 12 who were ventilated for 1 or more days. Between these groups, no significant differences were observed for mortality, wound infections, respiratory infections, days to full enteral feeding, bowel complications (reoperation and necrotizing enterocolitis) and length of stay. Owen et al. [25] compared silo staged closure without ventilation with primary surgical reduction. Results on all evaluated outcomes were comparable. The following conclusions were drawn:

**Table:** 

	Grade
There are cautious indications that silo treatment without intubation and ventilation results comparable outcomes on wound infections, respiratory infections, days to full enteral feeding, bowel complications (reoperation and necrotizing enterocolitis) and length of stay compared to silo-staged closure in ventilated infants [26]	Very low
There are cautious indications that silo treatment without intubation and ventilation and without general anesthesia results in comparable outcomes on wound infections, respiratory infections, days to full enteral feeding, bowel complications (reoperation and necrotizing enterocolitis) and length of stay compared to primary surgical closure [25]	Very low

Although the evidence is scarce, of low quality and indirect, no indications of particular harms of a silo staged closure without anesthesia were found. One positive effect is the avoidance of mechanical ventilation for the patient, it is an objective benefit for the newborn lung and also would diminish the need of sedation, risk of respiratory infections and would increase comfort and parents-newborn interaction. Another positive effect that can be hypothesized refers to the avoidance of early-life exposure to general anesthetics. The panel recognized that this evidence is limited but feels that the balance of effects could lean towards treatment without ventilation and general anesthesia in patients with simple gastroschisis and stable condition, which makes it reasonable to at least consider this treatment as an option. If staged reduction without ventilation and general anesthesia is chosen, adequate measurement and management of pain throughout the process is mandatory in these patients.

2.5. Patches

2.5 What are the (un)favorable effects of non-absorbable (synthetic) versus biological patches in neonates with gastroschisis if fascial closure is not possible?

No comparative evidence was found by using these inclusion criteria, therefore, the date and minimum sample size inclusion criteria were dropped. With this adjustment, there was still no evidence found comparing the outcomes for synthetic and biological patch materials. Anecdotal evidence is described in the complete guideline, available at the ERNICA website.

**Table:** 

	Grade
There is a lack of evidence comparing the outcomes of synthetic and biological patches and therefore, no conclusions can be drawn	–

Professionals on the guideline panel agree that there are no firm conclusions to recommend specific mesh above other for the closure of the large abdominal wall defects that can´t be closed conventionally. It is known that the combination of treatment in high volume centers and treatment according to surgeon’s expertise is associated with better outcomes. Therefore, the hospital taking care of this group of patients should select their own method.

### Module 3: Feeding

3.1 Starting enteral feeding

3.1a What are the (un) favorable effects of starting enteral feeds at < 7 days of life in newborns with gastroschisis?

Only one study was found that analyzed the effect of timing to first feed on infection risk with a clear cut-off at 7 days. Aljahdali et al. [27] analyzed the effect of timing of first feeds (TTFF) on outcome. Differences between four groups were analyzed. TTFF was ≤ 7 days (n = 70) for group 1, 8–14 days (n = 253) for group 2, 15–21 days (n = 152) for group 3 and > 21 days (n = 95) for group 4. Outcomes sepsis or surgical site infection and length of stay could be analyzed. Groups 3 and 4 both had significantly higher rates of surgical site infection (SSI) compared to group 1 (OR 2.4 respectively 4.8), but there was no significant difference between group 1 (≤ 7 days) and group 2 (8–14 days). Duration of parenteral nutrition and length of stay were both observed to be longer for infants in group 1 compared to group 2. Evidence from a meta regression study suggests that delay in the start of enteral feeding is associated with longer hospital stay, longer time to full enteral feeds and longer use of parenteral nutrition [28]. The following conclusions were drawn:

**Table:** 

	Grade
There are cautious indications that early enteral feeds starting within 14 days are associated with a lower relative risk for surgical site infection as compared to start of enteral feeding after 14 days, but that the risk of starting before or after 7 days is comparable [27]	Very low
There are cautious indications that enteral feeding < 7 days is associated with longer mean PN duration and longer mean hospital stay as compared to start of enteral feeding between 8 and 14 days [27, 28]	Very low

The evidence is too weak to make a judgement, especially because the main outcomes (infection rates and time on PN) are in favor of different directions when comparing feed initiation at < 7d with feed initiation at > 7 d. However, it all outcomes were worse when starting the feeds later than day 15. In most children clinical signs can indicate if feedings can be started/increased or not. If there is significant bilious vomit then it is probably not the right time to start. However, if a baby has only small aspiration volumes, it is reasonable to try and start or to increase the feeds.

3.1 Starting enteral feeding

3.1b What are the (un)favorable effects of a feeding protocol for the initiation of enteral feeding for patients with Gastroschisis?

Data was found on the outcomes sepsis or infection, time to full enteral feeds, time on parenteral nutrition and length of stay. One study by Lemoine et al. [29] compared a traditional feeding strategy to a new early feeding protocol. Their intervention protocol includes cycling of suction at routine intervals with standardized increase of intake. The relative risk of sepsis in the traditional group compared to the intervention protocol group was 3.5 but this did not reach significance, probably due to the small sample. The meta-analysis by Raduma et al. [30] included studies that compared patients treated with a feeding protocol to patients treated without a feeding protocol. Time to full enteral feeding was not significantly different between the protocolized and unprotocolized group. Results from the more recent study by Utria et al. [31] indicate protocol-fed babies reached goal feeds not only faster (14 days from start of enteral feeding vs 20 days), but were also younger at goal feeds (26 days vs 34 days, *p* = 0.001) compared to the control group. No significant differences in time on parenteral nutrition and length of stay were observed [30]. The following conclusions were drawn:

**Table:** 

	Grade
It is unclear whether a feeding protocol is associated with a significant smaller risk of infections [29]	Very low
There are cautious indications that there is no significant difference in time to full enteral feeds, time on parenteral nutrition and length of hospital stay between patients treated with a feeding protocol and controls [30]	Very low

There are indications that protocolized feeding is associated with lower risks for septicemia, while no associated harms were reported. It is likely that parents and caregivers of gastroschisis patients may have a positive opinion of the existence of a feeding protocol. The panel recognizes the mechanism of higher standardization leading to better care and lower costs, with a recent study indicating a decrease of hospital costs for gastroschisis with almost 10% after the implementation of a feeding protocol [31].

3.2 Central line placement

3.2 What is the preferred location for central venous line placement in patients with gastroschisis, considering upper versus lower extremity?

The retrospective study by Ma et al. [32] compares outcomes of upper versus lower extremity lines in patients with gastroschisis. No studies were found on non-cuffed central venous lines or broviac lines. There is only 1 retrospective study on complication rates of upper versus lower extremity PICCs in patients with gastroschisis (n = 138). Complications were defined as one or more of the following: infiltration, phlebitis, occlusion, migration, infection and thrombosis. This study suggests the risk of complications (mainly infiltration and phlebitis) is significantly lower when the line is placed in the upper extremity (RR 0.17, 95% CI 0.05 to 0.60. In group treated with SILO, the overall complication risk was over 9 times higher when a PICC line was placed in the lower extremity versus the upper extremity.

**Table:** 

	Grade
There are cautious indications that placement of a PICC line in the upper extremity is associated with a lower risk for complications [32]	Very low

Even though the quality of evidence is low the panel judged that the balance of effects leans towards placement in the upper extremity. As far as the current evidence tells, the upper extremity placement knows no particular harms compared to the lower extremity and does know benefits.

If complications such as infection and phlebitis are higher with a lower extremity line, suggesting the upper extremity line as a first choice can cause an increase in equity amongst patients treated in different hospitals as this results in equal estimated risks for infection for all patients. If the upper extremity placement fails, a lower extremity or central venous catheter (for example in the neck) can be considered.

### Module 4: Organisation of care

#### Patient perspectives to the gastroschisis care pathway

Due to the lack of European patient representative associations for gastroschisis, the patient’s perspective was included through a survey. Thirty families from 6 countries (Lithuania, Italy, Spain, Germany, France and Sweden) were contacted, with a 77% (23/30) response rate. The questions covered 4 domains: prenatal care, surgical treatment, feeding and organization of care. Additionally, parents were asked about their suggestions to improve the course of care. The complete survey is available in the supplementary materials (Additional file 4: Appendix D) and was translated into the common language for each recruiting centre.

All parents received enough information on why it is important to follow up pregnancies with gastroschisis. The majority of parents (87%) had the opportunity to discuss the mode of delivery with their doctor (87%), and to discuss the timing of delivery (70%). Any form of counselling before the surgery/treatment about the treatment options for closure of gastroschisis was offered to 91% of the parents.

A specific feeding protocol existed in only 61% of the hospitals, but all those parents found it helpful. However, the majority of the parents (91%) did not miss any kind of support/information regarding feeding the child while in the hospital.

### Organization of care

Regarding information obtained on gastroschisis, most of the parents (78%) received information on gastroschisis from their hospital, spoken and/or written, 65% searched google, and few (9%) looked into the ERNICA website.

Other mentioned sources of information were Facebook, and contact with other parents.

All parents received information on what to expect of the care pathway, from hospital stay, to discharge and follow up.

After discharge, the majority of the parents (91%) did not miss any kind of support/information with regards to feeding the child.

Social counseling or (psychological) support (before and/or after birth), was offered to 3 out 4 of the families, but only 1 out of 2 who obtained support found it helpful. Some families (17%) who did not receive psychological support did miss it.

Half of the parents were able to get in contact with other parents with a child with gastroschisis (before and/or after birth) and those parents found it helpful. One out of four families did not get in contact with other families, but would sooner have had the opportunity.

One third of the families found other parents going through the same issues on social media and got in touch with others without any organization, 1 out 4 through a closed Facebook group, only 9% through a parental support group organized by the hospital.

All parts of care, such as ward visits, information folders, websites or encounters with professionals (nurses, doctors, social workers) were perceived as very helpful during pregnancy and after birth, but also social media, Google, websites and written folders after discharge.

### Suggestions for improvement

Suggestions for improvement in the care, treatment, diagnosis or counselling of a child with gastroschisis were the possibility to obtain psychological support before and after birth, before and after discharge, to provide more information in written folders, parents support groups, to allow relatives in the NICU for support, sedation/analgesia protocols during reduction of bowels in the NICU and structured and early feeding protocols.

### Counselling

Most babies with gastroschisis will be diagnosed prenatally, therefore counselling, with its share of uncertainty, will focus on a yet unborn child. Ideally, counselling should be done by the pre and post-natal teams together to ensure that the information given is consistent and that the dialogue is homogeneous. Decisions regarding antenatal follow-up, the timing, place and mode of delivery, as well as the procedures to be performed during the neonatal period should be discussed by the medical team prior to consultation with the parents [33]. The pediatric surgeon mainly relies on the information delivered by the fetal medicine specialist through ultrasound and/or MRI images. The surgeon is unable to examine the future patient, but should attempt to give prognostic information about the severity of the malformation, the possible surgical repair, the expected morbidity, length of hospital stay and the potential complications, including death in severe cases. This task should be sustained by good knowledge of the natural history and prognosis of the prenatal condition, which may differ from the same condition diagnosed postnatally [33].

Gastroschisis is diagnosed more and more often in the first trimester at a stage of pregnancy when abortion is legal in many countries and a detailed description of the care pathway could be extremely distressing especially for a young primipara. The main concern of the prenatal team is to provide sufficient information to allow the parents to make an informed decision and to respect and protect parental autonomy [34] Parents should be informed that regular prenatal ultrasound follow-up refines the prognostic assessment and detects rare associated anomalies that may alter the prognosis. Therefore, giving accurate prognostic information from the first trimester onwards is difficult. However, in their paper on the effect of repeated consultations on parental anxiety, Aite et al. [35] conclude that early antenatal diagnosis should be encouraged in order to maximize the chance of repeated consultations for the prospective parents. Marokakis et al. [36] in a systematic review on prenatal counselling report that most parents preferred to attend counselling as soon as possible after prenatal diagnosis to reduce the stress associated with waiting. A minority expressed satisfaction with being given some time to process the diagnosis before counselling.

In some situations, it is mandatory to inform patients that the precise diagnosis is unknown and/or that the prognosis is uncertain. Most patients will appreciate sincerity and will understand the diagnostic and prognostic evaluation process even better when the fetal medicine specialist and the surgeon carry the same messages. Sincere counselling never jeopardizes the trust that parents put in the medical team [33].

The guideline development panel discussed extensively on the question if any possible complication should be explained to the parents. For example, should prenatal counselling regarding a fetus with gastroschisis include mention of the rare occurrence of bowel necrosis? The panel agrees that clear, concise and complete information should be given to parents. Potential major complications should be explained, but so should the possibility of diagnosing some of them early by ultrasound examination. If a severe complication does occur, the parents will then be prepared for the worst outcome. It is, however important to explain to parents the probabilistic nature of such counseling. The findings of follow-up studies are always couched in statistical terms and are not always easily applicable to individual cases [33].

An expert surgical consensus for prenatal counseling using the Delphi method [37] has been published for congenital pulmonary airway malformation and congenital diaphragmatic hernia but not for gastroschisis. Parents of children born with a malformation diagnosed prenatally need to be offered proper psychological support during the pre- and postnatal period [38]. The risk of after-effects similar to post-traumatic stress should not be overlooked [39].

### Multidisciplinary team

There is increasing evidence that a multidisciplinary team approach and the implementation of standardized feeding protocol improves outcome in patients with short bowel syndrome [40–43] However, the evidence regarding gastroschisis is still weak, due to the complexity, variability and rarity of the condition [44] as it is reflected in this guideline. Therefore, we aim to propose multidisciplinary teams as the gold standard in the management of gastroschisis in expert centers, to enhance communication of the individualized treatment plan to the family/patient, and to keep the continuity of the patient centered care throughout the entire treatment process and follow up.

Such a multidisciplinary team should cover all aspects of the condition, including maternal–fetal specialist, obstetrician, neonatologist, pediatric surgeon, pediatric anesthetist, pediatric gastroenterologist, dietitian, social worker and/or psychologist and coordinating nurse.

## Volume/outcome relationship

4.4 Is higher volume associated with better outcomes in gastroschisis care?

Volume/outcome relationships have been less studied in gastroschisis, but there is a recent comprehensive systematic review on the topic [45] which identified 12 studies reporting hospital volume / outcome relationships in gastroschisis. This review reported significantly lower mortality in higher volume than lower volume centres, whereas there was no clear association of other outcomes (LOS, sepsis, time on PN) with volume.

**Table:** 

	Grade
There are indications that higher volume centers have a significantly lower mortality rate compared to lower volume centers	Low

For the the included studies reporting mortality, high vs. low case volume per year was as follows:

1. High ≥ 14, medium 5–13, low ≤ 4
2. High median 9, medium median 4, low median 1
3. High ≥ 9, medium 5–8, low ≤ 4
4. High ≥ 11, medium 5–10, low ≤ 4
5. High ≥ 10**,** medium 3–9, low < 3

Hence it would appear that centres treating at least 9–10 cases per year have a lower mortality rate that those treating 4 or less cases per year. However, it has to be noted that the incidence of gastroschisis is decreasing [46]. Therefore the GDG urges to consider the several other possible factors contributing to lower mortality, including appropriate fetal medicine support (which is likely to be more prevalent in highly specialised centers), greater surgical experience, and more protocolised care.

### Quality of life: *which QoL measures are appropriate for gastroschisis patients?*

The literature search on QoL measures that had been used in gastroschisis yielded 9 papers published from 2010 in which QoL had been reported. Of these, 6 had used the PedsQL score[47–52] two had used the KIDCSREEN instrument [53, 54] and one had used an unvalidated scoring system [55].

Allin et al. (1) recommended that a validated QoL instrument should be used in reporting outcomes of gastroschisis, and agreement was reached that this should include the use of PedsQL. Specifically, the following recommendation was included for QoL in the final core outcome set:

Median (IQR and range) PedsQL score in each study group. If appropriate, the median (IQR and range) score from the PedsQL gastrointestinal symptoms and family impact modules in each study group should also be reported.

## Conclusion

This executive summary reports the results of the systematic development of the ERNICA guideline for Gastroschisis. To answer the predetermined clinical questions, 32 publications were included for analysis. This evidence-based guideline serves as a comprehensive and reliable resource for healthcare professionals and stakeholders involved in the management and treatment of Gastroschisis. By synthesizing the best available evidence including the most recent observational studies, systematic reviews and combining this evidence with expert consensus, this guideline provides clear and actionable recommendations for clinical practice. These recommendations are intended to optimize patient outcomes, improve the quality of care, and enhance decision-making processes.

### Future perspectives

It is essential to recognize that guidelines are living documents and should be regularly reviewed and updated as new evidence emerges. ERNICA is responsible for the maintenance of this document and will install a panel to review the currency and accuracy of the guideline no later than 2028. Because of the low or even very low quality of the evidence body for most subjects of this guideline, the GDG identified some research priorities for each topic covered in these guidelines of which a complete report is available in the supplementary materials (Additional file 4: Appendix D). For most topics goes that multicenter, prospective studies are the best route to higher quality evidence and more certainty on outcome estimates. Considering the identified knowledge gaps in the revision of the datasets for the gastroschisis registry is a way to make multicenter prospective database studies feasible. As ERNICA aims to facilitate continuous quality improvement and optimal implementation, the development of a set of quality and implementation indicators based on this guideline is planned for the near future.

### Supplementary Information

**Additional file 1. Appendix A:** Search strategy.

**Additional file 2. Appendix B:** Evidence tables and Grade profiles.

**Additional file 3. Appendix C:** Evidence to Decision tables.

**Additional file 4. Appendix D:** Survey for patient-parent perspective.

## Data Availability

All data is available and evidence tables are submitted as supplementary material.
