# Supplementary material for: European reference network for rare inherited congenital anomalies (ERNICA) evidence based guideline on the management of gastroschisis
Source: Orphanet J Rare Dis. 2024 Feb 12;19:60. doi: 10.1186/s13023-024-03062-8 (PMC10860293; doi:10.1186/s13023-024-03062-8)
Supplement: Supplementary file 1 — Additional file 1. Appendix A: Search strategy. [file 13023_2024_3062_MOESM1_ESM.docx]

**Appendix A : Search strategy**

*Systematic reviews*

**Embase.com 42**

(gastroschisis/mj OR (gastroschisis):ti) AND ('systematic review'/de OR 'meta analysis'/exp OR ((systematic* NEAR/3 review*) OR meta-analy* OR metaanaly*):ab,ti)

**Medline 35**

(* Gastroschisis/ OR (gastroschisis).ti.) AND (((systematic* ADJ3 review*) OR meta-analy* OR metaanaly*).ab,ti.)

1. **Gastroschisis prenatal**

1a/ 1b delivery mode

**Embase.com 86** (record)

(gastroschisis/mj OR (gastroschisis):ti) AND ('immature and premature labor'/mj/exp OR 'labor induction'/mj/exp OR 'cesarean section'/exp/mj OR 'mode of delivery'/mj OR (((immature OR premature OR preterm* OR term* OR induc* OR elective* OR schedule* OR timing OR planned OR mode OR weeks) NEAR/3 (labor* OR childbirth* OR birth* OR deliver*)) OR cesarean* OR caesarean* OR c-section*):ti) NOT [conference abstract]/lim AND [english]/lim NOT ([animals]/lim NOT [humans]/lim)

**Medline ALL 62** (medlars)

(* Gastroschisis/ OR (gastroschisis).ti.) AND (* Obstetric Labor, Premature/ OR * Labor, Induced/ OR exp * Cesarean Section/ OR (((immature OR premature OR preterm* OR term* OR induc* OR elective* OR schedule* OR timing OR planned OR mode OR weeks) ADJ3 (labor* OR childbirth* OR birth* OR deliver*)) OR cesarean* OR caesarean* OR c-section*).ti.) NOT (conference abstract) AND english.la. NOT (exp animals/ NOT humans/)

**2 Prenatal ultrasound**

**Embase.com 175** (record 1)

(gastroschisis/mj OR (gastroschisis):ti) AND ('fetus echography'/mj OR 'prenatal diagnosis'/mj OR (((fetal OR fetus* OR foetal OR foetus* OR prenatal* OR perinatal* OR pre-natal* OR peri-natal* OR antenatal* OR ante-natal* OR intrauterin* OR uter*) NEAR/3 (ultraso* OR echograph* OR diagnos* OR sonogra*))):ti) NOT [conference abstract]/lim AND [english]/lim NOT ([animals]/lim NOT [humans]/lim)

**Medline All 113** (medlars 1)

(* Gastroschisis/ OR (gastroschisis).ti.) AND (* Prenatal Diagnosis/ OR (((fetal OR fetus* OR foetal OR foetus* OR prenatal* OR perinatal* OR pre-natal* OR peri-natal* OR antenatal* OR ante-natal* OR intrauterin* OR uter*) ADJ3 (ultraso* OR echograph* OR diagnos* OR sonogra*))).ti.) NOT (conference abstract) AND english.la. NOT (exp animals/ NOT humans/)

1. **Closure of the abdominal wall**

**Embase.com 56** (record 2)

(gastroschisis/mj OR (gastroschisis):ti) AND (surgery/mj OR 'abdominal surgery'/mj OR 'abdominal wall closure'/mj OR (surger* OR surgical* OR closure* OR silo):Ab,ti) AND ('clinical trial'/exp OR (trial* OR rct OR random*):Ab,ti) NOT [conference abstract]/lim AND [english]/lim NOT ([animals]/lim NOT [humans]/lim)

**Medline ALL 36** (medlars 2)

(* Gastroschisis/ OR (gastroschisis).ti.) AND (* General Surgery/ OR (surger* OR surgical* OR closure* OR silo).ab,ti.) AND ((trial* OR rct OR random*).ab,ti.) NOT (conference abstract) AND english.la. NOT (exp animals/ NOT humans/)

1. **Feeding**

Enteral feeding

**Embase.com 17** (record 3)

(gastroschisis/mj OR (gastroschisis):ti) AND ('enteric feeding'/mj OR ((enteric* OR enteral* OR tube* OR intragastric* OR intestin* OR protocol* OR timing OR early OR late) NEAR/3 (feed* OR nutrition*)):ti) NOT [conference abstract]/lim AND [english]/lim NOT ([animals]/lim NOT [humans]/lim)

**Medline ALL 23** (Medlars 3)

(* Gastroschisis/ OR (gastroschisis).ti.) AND (* Enteral Nutrition/ OR ((enteric* OR enteral* OR tube* OR intragastric* OR intestin* OR protocol* OR timing OR early OR late) ADJ3 (feed* OR nutrition*)).ti.) NOT (conference abstract) AND english.la. NOT (exp animals/ NOT humans/)

CVL

**Embase.com 1** (record 4)

(gastroschisis/mj OR 'abdominal wall defect'/exp/mj OR (gastroschisis OR (abdom*-wall* NEAR/3 (defect* OR hernia* OR lesion* OR aplas* OR insuffic* OR rupture*)) OR omphalocel*):ti) AND ('central venous catheter'/mj OR ((central-ven* NEAR/3 (catheter* OR line*)) OR cvl):ti) NOT [conference abstract]/lim AND [english]/lim NOT ([animals]/lim NOT [humans]/lim)

**Medline ALL 3** (medlars 4)

(* Gastroschisis/ OR (gastroschisis OR (abdom*-wall* ADJ3 (defect* OR hernia* OR lesion* OR aplas* OR insuffic* OR rupture*)) OR omphalocel*).ti.) AND (* Central Venous Catheters/ OR ((central-ven* ADJ3 (catheter* OR line*)) OR cvl).ti.) NOT (conference abstract) AND english.la. NOT (exp animals/ NOT humans/)
