# Supplementary material for: European reference network for rare inherited congenital anomalies (ERNICA) evidence based guideline on the management of gastroschisis
Source: Orphanet J Rare Dis. 2024 Feb 12;19:60. doi: 10.1186/s13023-024-03062-8 (PMC10860293; doi:10.1186/s13023-024-03062-8)
Supplement: Supplementary file 2 — Additional file 2. Appendix B: Evidence tables and Grade profiles. [file 13023_2024_3062_MOESM2_ESM.docx]

**Appendix B : Evidence tables and Grade profiles**

### Module 1

*Question 1.1a*

| Reference | Study details | Patients and inclusion | Intervention and study aim | Control/vergelijking | Follow-up | Outcomes of interest and effect | Notes: |
| --- | --- | --- | --- | --- | --- | --- | --- |
| Landisch (2017)  J Pediatr Surg | Systematic review and meta-analysis of 13 studies (12 observational and 1 randomized controlled trial), data collection between 1997 and 2015 (USA, Canada, UK, Portugal, Norway, New Zealand, France, Netherlands)  Funding and conflict of interest:  None declared | n= 1430  - 772 preterm  - 658 controls  Inclusion criteria:  - English language peer reviewed articles (MEDLINE, PubMed, Scopus and Cochrane)  - Reporting of neonatal outcomes for infants with gastroschisis  - Studies with a preterm and comparative control group  Exclusion criteria  - Case reports | Pooled estimate of the OR using a random effects model (DerSimonian and Laird).  I^2^ statistic for interstudy heterogeneity evaluation.  Significancy p<0.05. | Intervention: preterm delivery (elective or for unknown reasons)  Control: managed expectantly or born at term gestational age  Group 1 (G1): Prospective or retrospective studies with elective preterm delivery at a designated GA or a stated plan for an elective delivery for gastroschisis before spontaneous labor  Group 2 (G2): Prospective or retrospective studies using a GA cut-off as a surrogate for elective preterm delivery  Group 3 (G3): Retrospective studies using elective preterm delivery and/or GA cut-off, but with significant limitations | Not reported | Evaluated outcomes for the guideline:  Death **(1)** and Neonatal Sepsis **(2)** and Severe gastrointestinal complication **(3)** and Time on parenteral nutrition **(4)**  **(1)**  “No difference” (G1 & G2)  I^2^=0  8 studies  N total = 538  **(2)** G1  OR 0.43 (0.24-0.78) ; p=0.006 ; I^2^=0  3 studies  N total = 253  G2  OR 4.90 (0.68-35.16) ; p=0.11 ; I^2^=0.40  2 studies  N total = 65  **(3)**  **Necrotizing enterocolitis** “No difference” (G1 & G2)  I^2^=?  4 studies  N total = 274  **(4)**  G1  Mean difference -11.24 (-23.23 -0.75) ; p=0.066 ; I^2^=0.87  6 studies  N total = 591  G2  Mean difference 9.84 (-3.16-22.85) ; p=0.138 ; I^2^=0.90  4 studies  N total = 270 | No definition for sepsis or NEC was found in included studies.  Major heterogeneity in the definition of the groups  No attempt to contact the authors if the information was unavailable  One RCT: Logghe (n = 21 / 21)  Subcategorization of the analysis based on elective preterm delivery (G1 studies) distinct from preterm GA (G2 studies) aimed to foster a clearer interpretation of results of the meta-analysis |
| Grant (2013)  Cochrane | Systematic review and meta-analysis of 1 RCT (Logghe 2005),  Funding and conflict of interest:  None declared | n= 42  - 21 planned early birth  - 21 planned later birth  Inclusion criteria:  - Articles (CENTRAL, Embase, MEDLINE, BioMed Central, handsearches of 30 journals)  - No language restriction  - Clinical trials  - Reporting of neonatal outcomes for infants with gastroschisis | None (1 RCT) | Intervention: Planned preterm birth (< 37 WG)  Control: Planned later birth (>= 37 WG) | Not reported | Evaluated outcomes for the guideline:  Death **(1)** and and Severe gastrointestinal complication **(3)**  **(1)** RR 5.00 (0.26-98.00) ; p=0.29 ;  N total = 20  **(3)**  **Necrotizing enterocolitis** RR 0.25 (0.03-2.05) ; p=0.20 ;  N total = 20 | Prof Thornton (co-author) is co-author of the included trial and assessed risk of bias  Logghe: Planned early birth = 36 WG  Control group: await spontaneous birth or need for elective birth for another reason  Seven women (33%) in the elective group and nine women (43%)  in the spontaneous group delivered by caesarean section (RR 0.78;  95% CI 0.36 to 1.70)  Small sample size (clinically important effects have not been ruled out)  Logghe study is also included in the systematic review of Landisch 2017 |

*Question 1b*

| Reference | Study details | Patients and inclusion | Intervention and study aim | Control/vergelijking | Follow-up | Outcomes of interest and effect | Notes: |
| --- | --- | --- | --- | --- | --- | --- | --- |
| Segel (2001)  AJOG | Systematic review and meta-analysis of 15 retrospective observational studies, data collection between 1983 and 2000 (USA, Canada, UK, Austria, New Zealand)  Funding and conflict of interest:  Not reported | n= 805  From 24 to 125/study  Inclusion criteria:  - English language peer reviewed articles (MEDLINE)  - Reporting of neonatal outcomes for infants with abdominal wall defects who delivered vaginally or by cesarean section  Exclusion criteria  - Case series  - Neonatal outcomes not abstracted from the data in the manuscript | Pooled estimate of the OR using both a random effects model (DerSimonian and Laird) and a fixed effects model (Mantel Haenszel).  Q statistic for interstudy heterogeneity evaluation.  Significancy p<0.10.  Continuous outcomes were pooled using a random effects model of the std difference of means between the treatment and control groups | Vaginal versus cesarean section | Not reported | Evaluated outcomes for the guideline:  Death **(1)** and Neonatal Sepsis **(2)** and Severe gastrointestinal complication **(3)** and Time on parenteral nutrition **(4)**  **(1)**  RR 1.14 (0.59-2.21) ; p=0.69 ; Q=0.12  12 studies  N total = 615  **(2)** RR 0.70 (0.30-1.62) ; p=0.41 ; Q=0.14  7 studies  N total = 262  **(3)**  **Necrotizing enterocolitis** RR 1.27 (0.35-4.58) ; Q=0.31  2 studies  N total = 84  **(4)**  RR -0.12 (-0.42-0.16) ; p=0.16 ; Q=0.53  5 studies  N total = 208 | Gastroschis and omphalocele outcomes were combined    No attempt to contact the authors if the information was unavailable  No RCT  No information on gestational age at birth  12 of 15 studies reported the proportion of abdominal wall defects detected prenatally (total 327/697; 47%)  No mention of how the infants were transported  No mention if transported infants were intubated.  Unscheduled and elective cesarean sections were pooled (except for 5/15 studies)  Small bowel obstruction and ischemic bowel data are reported but with lacking information on repeat laparotomy |
| Kirollos (2017)  BMJ | Systematic review and meta-analysis of 38 retrospective observational studies, data collection between 1983 and 2014 (USA, Canada, UK, Austria, New Zealand, Norway, Germany, Brazil, England, Jordan)  Funding and conflict of interest:  None declared | n= 6577  - 3019 vaginal deliveries  - 3558 cesarean sections  Inclusion criteria:  - English articles (Cochrane, Embase, MEDLINE, CINAHL, GoogleScholar, Web of Science, congress abstracts)  - Observational studies: cohorts, cross-sectional, and case-control studies  - Reporting of neonatal outcomes for infants with gastroschisis who delivered vaginally or by cesarean section  Exclusion criteria  - Case series | Pooled estimate of the RR using a random effects model.  I^2^ statistic for interstudy heterogeneity evaluation.  Significancy p<0.10.  Subgroup analysis: prenatally diagnosed (PD) cases. | Vaginal versus cesarean section | Not reported | Evaluated outcomes for the guideline:  Overall and Neonatal Death **(1)** and Sepsis **(2)** and Severe gastrointestinal complication **(3)** and Time on parenteral nutrition **(4)**  **(1)  Overall mortality** OR 0.82 (0.57-1.18) ; p=0.28 ; I^2^=0.38  30 studies  N total = 6185  *Subgroup analysis (PD)*  *OR 0.72 (0.35-1.49) ; p=0.38 ;*  *6 studies*  *N total = 615*  **Neonatal mortality**  OR 1.08 (0.54-2.15) ; p=0.83 ; I^2^=0.29  13 studies  N total = 1044  *Subgroup analysis (PD)*  *OR 0.76 (0.33-1.75) ; p=0.51 ;*  *5 studies*  *N total = 466*  **(2)** OR 1.35 (0.91-2.00) ; p=0.14 ; I^2^=0.37  12 studies  N total = 1301  *Subgroup analysis (PD)*  *OR 1.84 (0.65-5.16) ; p=0.25 ;*  *3 studies*  *N total = 436*  **(3)**  **Necrotizing enterocolitis** OR 1.21 (0.63-2.35) ; p=0.57 ; I^2^=0  7 studies  N total = 1271  **(4)**  OR -96 (-3.79 -1.88) ; p=0.51 ; I^2^=0.51  9 studies  N total = 719 | 33 studies from USA (33/38)  Overlap (Segel 2011) : 5 studies (Bethel, How, Kirk, Moretti, Swift)  Subgroup analysis between good and satisfactory quality studies (Newcastle-Ottawa scale) for overall mortality: no difference  No attempt to contact the authors if the information was unavailable  No long-term outcomes  No RCT  Publication year range = 33  No subgroup analysis for gestational age at delivery, location of birth, birthweight, elective versus unscheduled cesarean section |

**Question: 1a. Does time of delivery impact mortality and/or morbidity among fetuses with gastroschisis**

**Bibliography:** Landisch et al (2017) (1)

| **Certainty assessment** | | | | | | | **Results** | | | | **Certainty** | **Importance** |
| --- | --- | --- | --- | --- | --- | --- | --- | --- | --- | --- | --- | --- |
| **№ of studies  (patient)** | **Study design** | **Risk of bias** | **Inconsistency** | **Indirectness** | **Imprecision** | **Other considerations** | **GR 1**  **Events / patients** | **GR 2**  **Events / patients** | **Relative** | **Absolute** |  |  |
| **Mortality** | | | | | | | | | | | | |
| 8  (574) | **Meta-analysis of** 7 observational studies and 1 RCT | serious^a^ | not serious | not serious | not serious | See below^d^ | 21/313 (6.7%) | 10/261 (3.8%) | RR 1.75  95% CI 0.84 to 3.65 p= 0.57 | 29 more per 1000  In favor of birth at term  No difference between G1 and G2 studies | ⨁ VERY LOW | Critical |

| **Neonatal sepsis in elective preterm delivery versus spontaneous delivery**   \| 3  (253) \| **Meta-analysis**  3 observational studies \| serious^a^ \| not serious \| Some concerns ^c^ \| not serious \| Large effect (OR<0.5) \|  \|  \| **OR 0.43**  **(95CI 0.24-0.78)**  **P=0.006** \|  \| ⨁⨁ Low \| Major \| \| --- \| --- \| --- \| --- \| --- \| --- \| --- \| --- \| --- \| --- \| --- \| --- \| --- \| |
| --- | --- | --- | --- | --- | --- | --- | --- | --- | --- | --- | --- | --- | --- |

| **Severe gastrointestinal complication**   \| 4  (274) \| **Meta-analysis**  1 RCT (G1) Logghe  3 observational studies (G2) \| serious^a^ \| serious^b^ \| Some concerns^c^ \| not serious \| See below^d^ \| RCT 1/20 (5%)  3 obs studies (G2) 10/137 (7.3%) \| RCT 4/20 (20%)  3 obs studies (G2) 6/109 (5.5%) \| Necrotizing enterocolitis  RR 1.33   95%CI 0.50 to 3.53 p=0.57 \|  \| ⨁ VERY Low \| Major \| \| --- \| --- \| --- \| --- \| --- \| --- \| --- \| --- \| --- \| --- \| --- \| --- \| --- \| |
| --- | --- | --- | --- | --- | --- | --- | --- | --- | --- | --- | --- | --- | --- |

| **Time on parenteral nutrition in elective preterm delivery versus spontaneous delivery** | | | | | | | | | |
| --- | --- | --- | --- | --- | --- | --- | --- | --- | --- |
| 6  (591) | **Meta-analysis**  6 observational studies | serious^a^ | serious^be^  I^2^=0.87 | not serious | not serious | See below^d^ | G1 Mean difference -11.24 (95CI -23.23 - 0.75) ;;  6 studies  Not significant (p=0.066) | ⨁ VERY LOW | Minor |

**CI:** confidence interval

Explanations

1. No attempt to contact the authors if the information was unavailable, High risk of bias on comparability of groups as assessed by NOS risk of bias (see Table 2. Landish et al.)
2. I^2^ < 50% or unreported*,
3. No definition for sepsis or necrotizing enterocolitis was found in included studies.
4. Group 1 (G1): Prospective or retrospective studies with elective preterm delivery at a designated GA or a stated plan for an elective delivery for gastroschisis before spontaneous labor

Group 2 (G2): Prospective or retrospective studies using a GA cut-off as a surrogate for elective preterm delivery

e. CI of mean difference crosses zero

* The corresponding author of the study has been contacted for clarification of the results but has not responded

**Question: 1b. Does mode of delivery impact mortality and/or morbidity among fetuses with gastroschisis**

**Bibliography:** Kirollos et al (2017),

| **Certainty assessment** | | | | | | | **Results** | **Certainty** | **Importance** |
| --- | --- | --- | --- | --- | --- | --- | --- | --- | --- |
| **№ of studies** | **Study design** | **Risk of bias** | **Inconsistency** | **Indirectness** | **Imprecision** | **Other considerations** |  |  |  |

**Neonatal mortality**

| 13  (1044) | **Meta-analysis**  Retrospective observational studies | Some concerns ^a^ | not serious  I^2^=0.29 | not serious | CI of effect crosses null |  | OR 1.08 (0.54-2.15) ; p=0.83 ; | ⨁ Very LOW | Critical |
| --- | --- | --- | --- | --- | --- | --- | --- | --- | --- |

| **Neonatal sepsis** | | | | | | | | | |
| --- | --- | --- | --- | --- | --- | --- | --- | --- | --- |
| 12  (1301) | **Meta-analysis**  Retrospective observational studies | Some concerns ^a^ | not serious  I^2^=0.37 | serious^d^ | CI of effect crosses null |  | OR 1.35 (95CI 0.91-2.00) ; p=0.14 ; | ⨁ Very Low | Major |
| **Severe gastrointestinal complication** | | | | | | | | | |
| 13  (1271) | **Meta-analysis**  Retrospective observational studies | Some concerns ^a^ | not serious  I^2^=0 | serious^d^ | CI of effect crosses null |  | *Necrotizing enterocolitis*  OR 1.21 (95CI 0.63-2.35) ; p=0.57 ; | ⨁ Very Low | Major |
| **Time on parenteral nutrition** | | | | | | | | | |
| 9  (719) | **Meta-analysis**  Retrospective observational studies | Some concerns ^a^ | serious^b,c^  I^2^=0.51 | not serious | CI of effect crosses null |  | OR -96 (95CI -3.79 - 1.88) ; p=0.51 | ⨁ Very Low | Minor |

**CI:** confidence interval

Explanations

1. No attempt to contact the authors if the information was unavailable

Study 2: Publication year range = 33

1. I^2^ > 50%
2. Duration till first enteral feed
3. No definition for sepsis or necrotizing enterocolitis was found in included studies.

*Question 1.2*

| Reference | | Study details | | Patients and inclusion | | Intervention and study aim | | Control | | Follow-up | Core outcome set:A-mortality/IUDB-sepsis,C-growth,D-no of operations,E-severe gastrointestinal complication,F-time on parental nutrition,G-liver disease,H--quality of life for childI-LoS | | Notes: added IUD and LoS | |
| --- | --- | --- | --- | --- | --- | --- | --- | --- | --- | --- | --- | --- | --- | --- |
| Lap (2020) | | Nationwide prospective study | | Population:  Isolated fetal gastroschisis (n=104, 103 sigleton, 1 dichorionic twin pregnancy) of which 4 IUFD.  81 simple gastroschisis and 19 complex gastroschisis.  Minor additional  congenital abnormalities were unilateral clubfoot in one  child and hydronephrosis in six children. | | To  identify antenatal ultrasound markers that could differentiate  between complex and simple gastroschisis.  Parameters included: bowel dilatation, polyhydramnios, fetal biometry, blood flow pattern of the superior  mesenteric artery (SMA) | | Refernece values of healthy controls | |  | Evaluated outcomes for the guideline (E):  E) No difference between simple and complex gastroschisis cases in growth (AC and EFW) or umbilical artery flows.  **IABD** normal in simple gastroschisis (comparable to normal size colon). **IABD and EABD** larger diameters compared to healthy controls.  **IABD and EABD** larger in complex gastroschisis compared to simple gastroschisis (p<0.001 and p<0.005, resp.).  **>2 IABD** ≥97.7th percentile is associated with complex gastroschisis (OR 4.39 (95%CI, 1.46–13.21); P=0.009, RR 1.56 (95%CI, 1.02–2.10); P=0.006, sensitivity 40.9%, specificity of 86.4%, positive and negative predictive values of 50.0% and  81.4%, resp.)  **EABD** trajectories (multiple measurtements) showed no association with presence of complex gastroschisis.  Overall, 83% of  intra-abdominal and 89% of extra-abdominal **SMA-PI** measurements were below the median (50th percentile) of the normal reference ranges. The lowest PI values  were found for the extra-abdominal SMA. Simple and complex-gastroschisis cases had similar developmental trajectories for the intra- and extra-abdominal  SMA-PI measurements and they were were not predictive of complex gastroschisis. There was no  significant relationship between SMA-PI and the degree of bowel dilatation. | | 27 excluded: 1 IUFD <20 weeks’ GA, 12 TOP, 14 did not want to participate.  Bowel atresia in 94.7% (18/19) of  cases with complex gastroschisis. The remaining case  had perforation of the proximal jejunum without atresia.  Six cases with complex gastroschisis had >1 additional intestinal disorder.  *Three postnatal deaths occurred: 1 (1.2%) with simple gastroschisis and two (10.5%) complex-gastroschisis (P=0.09). Neonatal mortality significantly higher in cases with polyhydramnios (22.2% = 2/9 (1 simple 1 complex case)*  *than in those without (1.1% = 1/90 complex, P=0.02).* | |
| D'Antonio (2015) | | A meta-analysis  Data collection till 2014  Funding and conflict of interest:  no | | N=2023 | | Evaluated separately the  association between 6 potential  predictors (IABD, EABD, GD,  polyhydramnios, SGA) and 6 adverse  clinical outcomes (IUD, NND, BA,  TFEF, LTPN, LOS). | |  | |  | Evaluated outcomes for the guideline:  **(A)** IABD whereas the risk of IUD or  neonatal death was not significantly higher than fetuses without IABD  **(E)** IABD higher risk of  BA diagnosed at surgery (OR: 5.48, 95%  CI 3.1–9.8.  **(F)** **TFEF** - did not report any significant increased risk in fetuses showing **IABD** (2 studies).  Only 1 study explored the association  between **EABD** and TFEF and found that fetuses with EABD 25 mm had  significantly longer times to reach the  full enteral feeding (25.7 6 12.8 vs 18.2  6 9.9 days, P = .02) compared with  those without EABD. The only study exploring the association between EABD and LTPN could not find any significant association between this ultrasound sign and the observed outcome. | |  | |
| Ferreira (2021) | | Systematic review and meta-analysis  Data collection till 2020 | | Population:  **Complex GS** (GS with congenital intestinal atresia, necrosis, stenosis, perforation, or volvulus)  N = 1440 | | US markers: **IABD, EABD,** IUGR, polyhydramnios, intestinal wall thickness, **bowel dilatation**, liver and bladder herniation, **delta dilatation and final bowel dilatation**, abdominal circumference, herniation, dilation of the stomach, size, and position of stomach, size of the abdominal wall defect, description of **mesenteric circulation**, collapsed extra-abdominal bowel, description of **peristalsis** and volvulus | | Isolated GS | |  | Evaluated outcomes for the guideline:  **(A)** 15,0% prevalence of deaths in newborns with complex GS  **(E)** IABD RR 3.01, 95% CI 2.22 to 4.08; I2 = 16%, p = 0.310. EABD RR 1.55, 95% CI 1.01 to 2.39; I2 = 77%, p = 0.000. | | **EABD** with a prevalence of 58.0% (95% confidence interval (CI), 0.37–0.79), 49.0% **IABD** (95% confidence interval (CI), 0.35–0.62). The statistical heterogeneity was substantial (I2 = 82.45%, p = 0.00). The meta-regression showed that heterogeneity had an influence on the results of the analysis (tau2 = 13.42, I 2 = 85.26%, Adj R-squared = 57.61%) | |
| Sun (2021) | A systematic review and meta-analysis  36 studies  IABD 12 studies  EABD 15 studies  BD-NOS 10 studies  Data collection till 25 December 2020  Funding and conflict of interest:  none | | Ultrasonographic markers were analysed:  bowel wall thickness (BWT), polyhydramnio, abdominal  circumference <5th percentile, gastric dilation (GD),  intra-abdominal  bowel dilation **(IABD),** extra-abdominal  bowel  dilation **(EABD)** and bowel dilation not otherwise specified  **(BD-NOS)**  N = 3054 | | The primary aim:  OR of  complex gastroschisis occurring in the presence of some specific  antenatal ultrasonographic markers (such as **IABD, EABD,** etc).  The secondary aim: OR of complex gastroschisis  occurring with other perinatal outcomes such as: (1)  length of hospital stay (LOS) in the neonatal intensive care unit  (NICU), (2) time to full enteral feeding (**TFEF**), (3**) postnatal**  **mortality**, (4) necrotising enterocolitis **(NEC)** and (5) short  bowel syndrome (SBS). | |  | |  | | | Evaluated outcomes for the guideline:  **(A) Postanatal mortality** IABD 5 studies (385 participants) OR or MD (95% CI) 1,73 (0,64-4,65) I^2^ 0%; EABD 7 studies (802 participants) ) OR or MD (95% CI) 0,98 (0,50-1,91) I^2^ 24%;  **(E Complex GS)**  IABD 13 studies (1083 participants) OR or MD (95% CI) 5.42 (3.24 to 9.06) I^2^33%  EABD 14 studies (1439 participants) OR or MD (95% CI) 2.27 (1.40 to 3.66) I^2^48%  BD-NOS  5 studies (426 participants) OR or MD (95% CI) 6.27 (1.97 to 19.97) I^2^65%  **(F) TFEF**  IABD 4 studies (334 participants) OR or MD (95% CI) 6.26 (-16.16,  28.67) I^2^79%  BD-NOS  3 studies (75 participants) OR or MD (95% CI) 10.65 (-1.66,  22.95) I^2^0% | | Complex gastroschisis has been defined as gastroschisis  associated with intestinal atresia, perforation,  volvulus and necrosis.  There was no significant difference between  fetuses with prenatal IABD and those without IABD, in terms of  postnatal mortality, NEC and TFEF  Second versus third  trimester analysis showed that IABD at ultrasound in either trimester  (T2: OR=19.70; 95% CI 1.69 to 229.06; p=0.022) and (T3:  OR=4.56; 95% CI 2.98 to 6.99; p<0.001) is significantly associated  with complex gastroschisis.  No significant difference was found between  EABD and non-EABD  groups regarding postnatal mortality, SBS and  LOS in hospital.  Second versus third trimester analysis showed that  only EABD detected in the third trimester was significantly associated  with higher odds of complex gastroschisis |
| Martilloti G, 2015 | Retrospective observational study  Cases from 2000 to 2011 | | N=119 | | **IABD** adjusted for GA  **EABD** (>13mm 2^nd^ trim or > 25mm 3 trim)  Stomach dilatation or herniation  Abnormal **Mesenteric** color and pulsed Doppler  Bowel lumen  Echogenic dilated loops  (2nd and third trimester)  For the prediction of perinatal outcome  (Primary outcome: complex GS) | |  | | 117  2 terminations | | | Evaluated outcomes for the guideline:  Death  **Death**: 16 dead, 101 Alive  2^nd^ trim US:  IABD diameter ratio observed/expected: 2.3 mm dead VS 1.9 m alive (p=0.006)  Perturbed mesenteric Doppler 6,3% in dead 3% in alive (p=0.049)  EABD no significant differences  3^rd^ trim US:  Perturbed mesenteric Doppler 25% in dead 5.9% in alive (p=0.030)  IABD and EABD no significant differences | | No definition of perturbed mesenteric Doppler |
| Frybova (2015) | Retrospective cohort study 2004-2013  (Czech republic) | | Population:  64 prenatally diagnosed neonates with gastroschisis | | To identify which  prenatal US findings (EFW, AFI, **IABD** (>10 mm), thickened bowel  wall (>3 mm), abdominal defect size or herniation of stomach) in fetuses with gastroschisis  correlate with complicated postnatal outcome. | |  | |  | | | Evaluated outcomes for the guideline (D, E, F, I):  (D) **prenatal intra-abdominal bowel dilatation is associated with greater number of additional surgical procedures (p<0.05)**  (E) prenatal intra-abdominal bowel dilatation is associated with presence of atresia (p<0.01)  (F) prenatal intra-abdominal bowel dilatation, thickened bowel wall and oligohydramnios (AFI <8 cm) is associated with longer administration of parental nutrition (median 53 vs. 21 days; p<0.05, median 34 vs. 20 days; p<0.01 or 30 vs. 16 days, p<0.05)  (I) prenatal intra-abdominal bowel dilatation or thickened bowel wall (=>3 mm) required (median 68 vs. 36 days; p<0.05 or median 44 vs. 37 days; p<0.05) | | Colon eventration, stomach herniation, and the size of the  defect of the abdominal wall measured at 30th week of  pregnancy did not correlate with adverse outcome. |
| Geslin (2017)  (In Sun 2021) | Retrospective multicentre (6 university hospitals) study between 2000-2013 (France) | | Population:  Prenatal diagnosis of gastroschisis (n=200)  Exclusion: TOP, miscarriage, lost to FU | | To evaluate prenatal ultrasound parameters (fetal growth, defect diameter, superior mesenteric artery index, intra-abdominal and axtra-abdominal bowel diameters (dilated definition: >6 mm inner-inner), changes in bowel appearance: hyperechogenic, segmental loss, increased peristalsis, bowel wall thickning) as prognostic factors for complex and vanishing gastroschisis | | Two/Three groups:  - simple gastroschisis (SG, n=148, 74%): without anatomical gastrointestinal abnormalities  -complicated gastroschisis (n=52, 26%): gastro-intestinal complications (n=42; bowel atresia (n=10), stenosis (n=8), volvulus (n=4), ischemia (n=2) and fibrous bands responsible for bowel wall compromise (n=24) or closing/ vanishing gastroschisis (n=10) | |  | | | Evaluated outcomes for the guideline (E):  (E) Increased risk of GI complications i.e. CG with smaller defect size (<9.2 mm at 2^nd^ trim and <12.5 mm 3^rd^ trim) and intra-abdominal bowel dilatation (defined as >19.0 mm - T2: n=8/73 without vs 14/31; p=0.0003 with and T3: n=25/107 without vs 23/39 with; p=0.0003)  No difference in extra-abdominal bowel diameter, bowel aspect. Unavailable data on mesenteric artery flows or AFI. | | Mortality rate: 4 IUFD \| 8 NND: 4/148 SG (extreme prematurity, anoxia, maternal sepsis, SID 3 months of age) & 4/52 (cardiac arrest postOR, bowel occlusion, short bowel syndrome, digesive bleeding)  Total IUGR 19/200 and significantly more in the CG group (p=0.01, n=10/52 vs 9/148)  Closing gastroschisis: clinical entity, composed of a closed wall defect associated with a small or vanishing herniated bowel, was confirmed by a fetopathologist examination or at birth |

**Question 1b: Can ultrasound findings predict outcome of patients with gastroschisis?**

**Bibliography:** Sun et al (2021)(1), D’Antonio et al (2015)(2), Ferreira et al (2021)(3), Frybova et al (2015)(4), Geslin et al.(2017)(5), Martillotti et al (2016)(6), Lap et al (2020)(7)

| **Certainty assessment** | | | | | | | **Results** | **Certainty** | **Importance** |
| --- | --- | --- | --- | --- | --- | --- | --- | --- | --- |
| **№ of studies** | **Study design** | **Risk of bias** | **Inconsistency** | **Indirectness** | **Imprecision** | **Other considerations** |  |  |  |
| **IABD for Mortality** | | | | | | | | | |
| 2  (1) | Systematic reviews, meta-analysis | Serious^a^ | Not serious | Not serious | Serious ^b^ | None | No significant correlation was found between IABD and intra uterine fetal death or neonatal death | ⨁⨁ Low | CRITICAL |
| **EABD for Mortality**   \| 2  (1) \| Systematic reviews, meta-analysis, observational studies \| Serious^a^ \| Not serious \| Not serious \| Not serious \| none \| No significant correlation was found between EABD and intra uterine fetal death or neonatal death \| ⨁⨁⨁ Moderate \| CRITICAL \| \| --- \| --- \| --- \| --- \| --- \| --- \| --- \| --- \| --- \| --- \|   **Mesenteric Artery flow for Mortality**   \| 2  (5,6) \| Observational studies \| Serious^a^ \| Not serious \| Serious ^c^ \| Not serious \| None \| There are cautious indications that mesenteric artery flow is altered in surviving foetuses with gastroschisis compared to those who died in one observational study (6). No definition for perturbed mesenteric flows was provided. \| ⨁ VERY LOW \| CRITICAL \| \| --- \| --- \| --- \| --- \| --- \| --- \| --- \| --- \| --- \| --- \| | | | | | | | | | |
| **IABD for Major gastrointestinal complications**   \| 1 (1) \| Systematic review, meta-analysis \| serious \| none \| none \| none \| none \| Studies have shown that there is a significant association between IABD (especially at second trimester) and complex gastroschisis. (1, 2 ,3)  Globally: OR=5.42 (95% CI 3.24 to 9.06) at second trimester: OR=19.70 (95% CI 1.69 to 229.06) and specificity of 96% (1) \| ⨁⨁⨁ Moderate \| CRITICAL \| \| --- \| --- \| --- \| --- \| --- \| --- \| --- \| --- \| --- \| --- \|   **EABD and Major gastrointestinal complications**   \| 1  (1) \| Systematic review, meta-analysis \| serious^a^ \| Not serious \| Not serious \| Not serious \| none \| Studies have shown that there is a significant association between EABD (especially third trimester) and complex gastroschisis. (1, 2 ,3)  OR=2.27 (95% CI 1.40 to 3.66) (1) \| ⨁⨁⨁ Moderate \| CRITICAL \| \| --- \| --- \| --- \| --- \| --- \| --- \| --- \| --- \| --- \| --- \| | | | | | | | | | |
| **IABD for Length of stay**   \| 1  (1) \| Systematic reviews and meta-analysis \| serious^a^ \| some ^d^ \| not serious \| Not serious \| none \| Mean difference in length of stay between IABD (+) and IABD(-) patients is 28.94 days [4.70-53.18] p=0.02 \| ⨁⨁⨁ Moderate \| Important \| \| --- \| --- \| --- \| --- \| --- \| --- \| --- \| --- \| --- \| --- \|   **EABD for Length of stay**   \| 1  (1) \| Systematic reviews and meta-analysis \| serious^a^ \| Serious ^e^ \| not serious \| Serious ^b^ \| none \| Mean difference in length of stay between EABD (-) and EABD(+) patients is -20.82, 95% CI -67.86 to 26.62; p=0.39; \| ⨁ Very Low \| Important \| \| --- \| --- \| --- \| --- \| --- \| --- \| --- \| --- \| --- \| --- \|   **IABD for Time on parenteral nutrition**   \| 1  (1) \| Systematic reviews and meta-analysis \| serious^a^ \| serious^f^ \| not serious \| Serious ^b^ \| none \| No significant correlation was found between IABD and TPN (1) \| ⨁ Very Low \| Important \| \| --- \| --- \| --- \| --- \| --- \| --- \| --- \| --- \| --- \| --- \|   **EABD for Time on parenteral nutrition**   \| 1  (2) \| Retrospective study \| serious^a^ \| some \| not serious \| Serious ^g^ \| none \| No significant correlation was found between EABD and TPN (2) \| ⨁ Very Low \| Important \| \| --- \| --- \| --- \| --- \| --- \| --- \| --- \| --- \| --- \| --- \|   **IABD for Number of operations**   \| 1  (4) \| Retrospective study \| serious^a^ \| serious \| not serious \| not serious \| none \| Prenatal intra-abdominal bowel dilatation (cut-off 10 mm) correlated with greater number of additional surgical procedures (<>0.05) \| ⨁ Very low \| Important \| \| --- \| --- \| --- \| --- \| --- \| --- \| --- \| --- \| --- \| --- \| | | | | | | | | | |

**CI:** confidence interval

Explanations

a. Due to inclusion of many studys with a retrospective design and no known correction for confounders. Heterogenic cut-offs used for IABD.
b. CI of the effect is very wide, crossing the null point.
c. No definition was given for perturbed mesenteric flows (6), in the other article information was very sparse.
d. I2 heteregeneity 68%
e. I2 heterogeneity 96%
f. I2 heterogeneity 79% .
G. Small sample, data comes from only one observational study

### Module 2

*Question 2.1*

| ***Reference*** | ***Study details*** | ***Patiënts and  inclusion*** | ***Intervention and study aim*** | ***Control/vergelijking*** | ***Follow-up*** | ***Outcomes of interest and effect*** | ***Notes:*** |
| --- | --- | --- | --- | --- | --- | --- | --- |
| *Pet et al 2017* | 1. Observational study,retrospective | Clinical characteristics and outcomes between 2 groups :  gastroschisis closure w/wo intubation  were compared. | Sutureless gastroschisis closure attempted without intubation  (n=23) (WR) | Sutureless gastroschisis closure with planned intubation in general anaesthesia (n=30  OR | Until discharge from hospital | **Mortality**  WR1/23  OR 0/30  OR = 4.1(95%CI = 0.16 – 104)  p=0.39  **Sepsis:-**  **Bowel ischemia**  WR 0/23  OR 1/30  OR = 0.42 (95%CI= 0.01-11) p=0.60    **TPN** >60days  WR 0/23  OR 4/30    OR = 0.12    **Hospital length of stay** ,days(median range)  WR 35.46 (31.27–60.61)  OR36.26 (26.67–79.44)  P = 0.85 |  |
| *Miyata et al, 2022* | Observational study,retrospective | rate of successful primary repair and post-operative outcomes between intubated and non-intubated patients | \|  \| Attempted closure without intubation (case) *N* = 115 (WR) \|  \| \| --- \| --- \| --- \| | Attempted closure with intubation (control) *N* = 271 (OR) | Until discharge from hospital | **Septicemia**      **Hospital length of stay, mean (SD) days**  WR 51.0 (51.)  OR 54.0 (61.1) p=0.63 |  |
| *Rao et al 2009* | Observational study,retrospective | compare the short-term outcomes of WR with the standard procedure of reduction under general anaesthesia | WR n=11 | OR n=27 | Until discharge from hospital | **Mortality**  WR 0/11  OR 0/27  OR=2.4 (95%CI=0.04 -128)  p=0.67  **Bowel ischemia**  WR 3/11  OR 1/27  **TPN >60days**  WR 2/11  OR 1/27  **Hospital length of stay,median (IQR),days**  WR 20 (15-49)  OR 23 (18-30)  OR= 1.21,95%CI= (0.50 - 2.97),p=0.67 |  |
| *Choi et al 2012* | Observational study,retrospective | Short-term and the long-term outcomes associated with sutureless WR vs others (n=70) | Sutureless WR (n=44) | Silo reduction (n=23)  OT reduction (n=3)   total OR n=26 | 7yrs 10mo | **Mortality**  WR 2/44  OR2/26  OR=0.57  95%CI (0.08-4.3)  p=0.57  **Septicemia**  WR 3/44  OR 4/26  OR=0.40 (95%CI = 0.1 – 2.0)  p=0.26  **Bowel ischemia**  WR 2/44  OR 3/26  OR=0.37  95%CI(0.1-2.3)  p=0.29  **Hospital length of stay ,days (median,IQR)**  WR26 (20.25-35.0)  OR 37 (30.0-44.0  p=not given |  |
| *Leadbeater et al 2010* | Observational study,retrospective | compare the outcomes of WR with tOR | WR (n=22) | OR (n=15) | Until discharge from hospital | **Mortality**  WR 0/22  OR 1/15  OR= 0.23, 95%CI=0.0 – 6.0)  p=0.38 |  |

**Question: What are the (un)favorable effects of primary fascial closure in the operating room (PC) compared to ward reduction and closure (bianchi’s approach, WR) in neonates with gastroschisis?**

Setting: Inpatient neonatology unit

**Bibliography: Pet et al. (2017)(12), Miyata et al., (2022) (14), 20), Rao  et al., (2009)(20), Choi et al.,(2012)(21), Ledbater et al.,(2010)(22)**

| **Certainty assessment** | | | | | | | **№ of patients** | | **Effect** | | **Certainty** | **Importance** |
| --- | --- | --- | --- | --- | --- | --- | --- | --- | --- | --- | --- | --- |
| **№ of studies** | **Study design** | **Risk of bias** | **Inconsistency** | **Indirectness** | **Imprecision** | **Other considerations** | **WR** | **PC** | **Relative(95% CI)** | **Absolute(95% CI)** |  |  |
| \| Mortality \| \| \| \| \| \| \| \| \| \| \| \| \| \| --- \| --- \| --- \| --- \| --- \| --- \| --- \| --- \| --- \| --- \| --- \| --- \| --- \| \| 4  (184)  (12, 20-22) \| observational \| serious^a^ \| not serious \| not serious \| not serious \|  \| 4/105  3.8% \| 3/79  3.8% \| OR 0.1.003  95%CI0.2 -4.6  p=0.9966 \| No difference \| ⨁  VERY LOW \| CRITICAL \|      \| Septicemia \| \| \| \| \| \| \| \| \| \| \| \| \| \| --- \| --- \| --- \| --- \| --- \| --- \| --- \| --- \| --- \| --- \| --- \| --- \| --- \| \| 1  (70)  (14) \| 1  observational \| serious^a^ \| not serious \| not serious \| not serious \|  \| 3/44  6.8% \| 4/26  15% \| OR =0.424  95%CI=(0.08 – 1.96)  p=0.26 \| **82 fewer per 1000** (from 140 fewer to 109 more) \| ⨁  VERY LOW \| CRITICAL \| \| Bowel ischeamia (severe gastrointestinal complication) \| \| \| \| \| \| \| \| \| \| \| \| \| \| 3  (165) \| 3  observational \| serious^a^ \| not serious \| not serious \| not serious \|  \| 5/78  6.4% \| 4/87  4.6% \| OR 1.412  95%CI0.4 -5.5  p=0.60 \| **26 fewer per 1,000**  (from 42 fewer to 40 more) \| ⨁  VERY LOW \| Important \| | | | | | | | | | | | | |
| Time on TPN  (>60 days) | | | | | | | | | | | | |
| 2  91 | 2  observational | serious^a^ | not serious  I | not serious | not serious |  | 2/26  7.7% | 5/65  7.7% | OR =1.00  95%CI=(0.18 – 5.51)  p=1.00 | NO DIFFERENCE | ⨁  VERY LOW | Important |
| Length of stay | | | | | | | | | | | | |
| 4  547 | 4  observational | serious^a^ | not serious | not serious | not serious |  | WR (n=175)  median 20-37 days (3 studies)  mean 51 days (1 study) | PC (n=372)  median 23-39 days (3 studies)  mean 51 days (1 study) | no statistical difference of NICU LOS in any of 3 studies  p = 0.60 – 0.67  p=0.63 |  | ⨁  VERY LOW | Important |

**CI:** confidence interval;

*Explanations*

ª High risk of methodological bias due to selection procedure. All patients for WR are selected, no randomization or all-inclusive intention to treat

*Question 2.2*

| ***Reference*** | ***Study details*** | ***Patiënts and  inclusion*** | ***Intervention and study aim*** | ***Control/vergelijking*** | ***Follow-up*** | ***Outcomes of interest and effect*** | ***Notes:*** |
| --- | --- | --- | --- | --- | --- | --- | --- |
| *Miyake et al, 2018* | Systematic Review and **meta-analysis**  comparing surgical closure (SC) under GA and plastic closure (PC)    6 studies (all retrospective single cohort)    **4 grouped and analysed addressing the PICO population** (after silo reduction):   1. Orion et al 2011 2. Machida et al, 2013 3. Schlueter et al 2015 4. Dariel et al, 2015 | Paper clearly distinguishes two different analysis based on population:    1) Undergoing primary closure (not of interest for PICO, not considered)    **2) Undergoing Silo reduction (of interest and considered):**  **a) 31 (PC 15, SC 16)**  **b) 5 (PC 3, SC 2)**  **c) 59 (PC 8, SC 51)**  **d) 64 (PC 23, SC 41)** | For analysis 2, of interest:    To asses efficacy and safety of plastic closure in gastroschisis patients undergoing Silo reduction | Surgical closure under GA in gastroschsis patients undergoing silo reduction | - | **Bold: considered in our COS reference**    **1) Mortality**  **2) Complications (wound infections)**  **3) duration ventilation**  **4)** feeding (4a: time to start; 4b: time to FEF**)**  **5) LOS**    1) 3 studies (a,b,d). 100 pts (PC 41, SC 59).  No diff (P 0,78, OR 1.39, CI 0.14-13.89)    2) 3 studies (b,c,d). 128 pts (PC 34, SC 94).  Favours PC (P 0,008, OR 0,24, CI 0.09-0.69)    3) 2 studies (a,b). 36 pts (PC 18, SC 8).  Favours PC (P 0.03, MD -5.76 (CI-10.93,-0.60)    4a) 2 studies (a,b). 36 pts (PC 18, SC 18).  Favours PC (P<0.0001, MD -9.42, CI -13.68,-5.16)    4b) 2 studies (a,b). 36 pts (C 18, SC 18).  No difference (P 0.19, MD -6.78, CI -17.03-3.46)    5) 2 studies (a,b). 36 pts (PC 18, SC 18).  Favours PC (P 0.002, MD -14.06, CI -22.86,-5.26) | 1 study includes complicated and simple GS, 3 only simple    Few outcomes when reporting COS considered for guidelines. For the specific PICO we should consider also other outcomes. |
| *Yousseff et al, 2016* | Systematic Review and meta-analysis comparing fascial closure (SC) or flap closure (plastic closure, PC) on population NOT addressing the PICO (includes primary reduction and after silo)    **BUT**    **There is a analysis for one outcome on a specific sub-group addressing the PICO (“exclusively after silo reduction”):**   1. Schleuter et al, 2015 2. Emami et al, 2015 3. Dariel et al, 2015 | Sub-group of interest:  460 gastroschisis pts undergoing silo reduction. 103 repaired by PC, 357 by SC:  a) 59 pts (PC 8, SC 51)  b) 337 pts (PC 72, SC 265)  c) 64 pts (PC 23, SC 41) | Sub-group of interest:  Comparison between flap (plastic) closure and surgical fascial closure exclusively in pts undergoing silo reduction | - | - | **Surgical Site infection:**    **Favours flap closure (P 0.01, OR 0.44, CI 0.24-0.82)** | 2 out of 3 papers analysed in this subgroup are the same reported in Miyake meta-analysis.    NOTE: Surgical site infection to be considered as “complication” minor outcome! |
| *Fraser et al, 2020* | Retrospective multicenter cohort study (Midwest pediatric surgery consortium) | Patients only simple gastroschisis undergoing plastic closure or surgical closure, either after primary or silo-staged reduction.    Total patients included: 315    **Specific analysis of PC vs SC under silo reduction:**  **- total patients: 242**  **- PC: 30**  **- SC: 212** | Comparison of Sutureless (PC) and Sutured (SC) after silo-staged reduction  of simple gastroschisis      (Analysis of same comparators are performed for patients undergoing primary reduction) | - | - | Outcomes:  **a) LOS**  b) **TPN duration**  c) use of anesthetics  d) Time to start feeding  e) time to FEF  **f) time of ventilation**  g) time birth to final closure  h) **Wound and deep infections**  i) days of antibiotics after closure  l)use of antibiotics after closure    a, **b**, d, e, g, **h**,i: **no statistical differences**    Statistically favours **PC in c (p<0,001), f (**ventialtor use **p 0,001, time on ventilator MD –3.5** days**, p 0,007),** and **l (p 0,001)** | Wound and deep infections are related to surgical site, no data on sepsis.    Data are not reported about anesthesia method during PC or SC closure |

**Question: What are the (un)favorable effects of surgical closure under general anesthesia compared tot cot-side plastic closure in neonates with gastroschisis who undergo staged closure (silo application)?**

**Setting: Inpatient neonatology unit**

**Bibliography: Miyake et al., 2018 (1), Youssef et al (2),  Fraser et al., 2020 (3)**

| **Certainty assessment** | | | | | | | ​​**№ of patients**​ | | **Effect** | | **Certainty** | **Importance** |
| --- | --- | --- | --- | --- | --- | --- | --- | --- | --- | --- | --- | --- |
| ​​**№ of studies**​ | **Study design** | **Risk of bias** | **Inconsistency** | **Indirectness** | **Imprecision** | **Other considerations** | **Sutured** | **plastic (sutureless)** | **Relative(95% CI)** | **Absolute(95% CI)** |  |  |
| \| Mortality \| \| \| \| \| \| \| \| \| \| \| \| \| \| --- \| --- \| --- \| --- \| --- \| --- \| --- \| --- \| --- \| --- \| --- \| --- \| --- \| \| (1) \| Meta analysis \| serious \| Not serious \| Not serious \| Serious \| none \| 1/59 \| 1/41 \| 1.39 [014.-13.89] \| 6 more per 1000 (from 15 fewer to 176 more) \| Very Low \| CRITICAL \|   **ROBINS-I and GRADE assessment already performed by Miyake et al for the outcome Mortality**   \| Surgical site/Wound infection \| \| \| \| \| \| \| \| \| \| \| \| \| \| --- \| --- \| --- \| --- \| --- \| --- \| --- \| --- \| --- \| --- \| --- \| --- \| --- \| \| (1), (2), (3) \| Meta analysis and observational studies \| serious \| Not serious \| Serious  B \| serious \| none \| 114/571 \| 16/136 \| RR 0.58 [0.36-0.96] \| 49 fewer per 1,000  (from 72 fewer to 5 fewer) \| Very Low \| CRITICAL \|      \| Length of Stay \| \| \| \| \| \| \| \| \| \| \| \| \| \| --- \| --- \| --- \| --- \| --- \| --- \| --- \| --- \| --- \| --- \| --- \| --- \| --- \| \| (1),, (3) \| Meta analysis and observational studies \| serious \| Not serious \| Not serious \| serious \| none \| 18 \| 18 \| -not estimable \| MD 14.06 **days fewer** (22.86 lower to 5.26 lower) \| /Very Low \| IMPORTANT \|   **ROBINS-I and Grade assesment already performed by Miyake et al (1) that is the main meta-analysis. Fraser et al (3) did not find statisticaaly difference between methods**  Ventilation time | | | | | | | | | | | | |
| (1), (3) | Meta analysis and observational studies | serious | Not serious | Not serious | serious | none | 18 | 18 | -not estimable | MD **5.76 days fewer** (10.93 fewer to 0.6 fewer)  c | Very Low | IMPORTANT |
| **ROBINS-I and Grade assessment already performed by Miyake et al (1) that is the main meta-analysis.**    Time on TPN | | | | | | | | | | | | |
| (3) | Retrospective cohort study | serious | Not serious | Not serious | serious | none | 212 | 30 | Not estimable | 27 vs 25 days, not significant (p=0.73) | Very Low | IMPORTANT |

**CI:** confidence interval; **MD:** mean difference

*Explanations*

ª High risk of methodological bias assessed with ROBINS-I with the main issue being bias due to confounding and outcome measurement (Miyake and Fraser results),  or NOS , with the main issue being group comparability (mixing of complex gastroschisis and simple in one analysis) (youssef paper) .

B  The primary outcome of interest is sepsis, this outcome is a surrogate chosen by a lack of data on sepsis. It is however logical that a sepsis would start with a surgical site/wound infection.

C The most recent data from an observational study (Fraser et al., 2020) supports that the effect is in favor of plastic closure (n=232). Their study resulted in 0.5 (0–4.75 IQR) days with sutureless approach versus  4 (1–7 IQR) with sutures (p= 0.007).

*Question 2.3*

| *Reference* | *Study details* | *Patients and  inclusion* | *Intervention and study aim* | *Control* | *Follow-up* | *Outcomes of interest and effect* | *Notes:* |
| --- | --- | --- | --- | --- | --- | --- | --- |
| 1. 5.2 Behram, 2021 | *Retrospective, 2b* | *29 patients:*  *23 simplex, 6 complex* | *To evaluate perinatal outcomes and surgical and clinical characteristics among GS patients based on their type of GS, abdominal wall closure method, and delivery timing.* | *simplex vs complex* | *NA* | *This study indicated that simple GS, compared with complex GS, was associated with improved neonatal outcomes. The authors suggest that the main factor affecting the patients’ outcomes is whether the patient is a simple or complex GS rather than the abdominal wall closure method.* | *Nice study with detailed protocol in material and methods.*  *Silo was used in both groups and for all patients*  *The authors suggest that if the cause of complex GS was intestinal atresia, the defect was closed, and a second surgery was performed within a few weeks to constitute bowel continuity.* |
| 1. 5.1 Bradnock 2011 | national cohort study | 393 GS:  251 simplex  31 complex | To describe one year outcomes for a national cohort of infants with gastroschisis. | simplex vs complex | 1 year fup | This study provides a comprehensive picture of current UK practice and outcomes that will inform and enhance the processes of antenatal counselling and postnatal prognosis The classification of gastroschisis into simple and complex groups,as defined by Molik et al,14 provides a pragmatic readily available method of risk stratification that we have now shown can predict outcome on a population based level | This population based cohort study of gastroschisis provides representative “benchmark” 1 year outcome data on a national basis. This study validates the classification of gastroschisis into simple and complex subgroups that predict length of stay in hospital, duration of parenteral nutrition, and risk of late complications. The lack of clinically relevant differences in most outcome measures between neonates managed with operative primary fascial closure or application of a preformed silo confirms the position of equipoise required for a national RCT |
| 1. [Riddle](https://pubmed.ncbi.nlm.nih.gov/?sort=date&term=Riddle+S&cauthor_id=34155327) J Perinatol . 2022 Feb;42(2):254-259. 5.1 | Retrospective | 2078 patients with 183 intestinal atresia | Compare in-hospital outcomes in gastroschisis with intestinal atresia versus simple gastroschisis (GS) using a national database. |  |  |  | A large multicenter cohort showed gastroschisis with atresia results in worse outcomes and complications, including necrotizing enterocolitis, feeding delays, and enteral feeding tube dependence. |
| 1. Ferreira 2021 5.1 | Systematic review: 44 studies included from 1156 papers | 3770  fetuses | To investigate the diagnosis, prognosis, delivery assistance, pregnancy results and postnatal management in gastroschisis |  |  |  | Confirm difference in outcomes between SG and CG  Some numbers are confusing |
| 1. *Bergholz 2014 5.1* | *SR/Metanalysis* | *Thirteen studies were included. All reported at least one or more of the above defined outcome parameters between newborns with sGS and cGS.*  *1301 patients with cCG* | *to evaluate the impact of complex cases on short term morbidity and mortality of gastroschisis.* | *Simplex gastroschisis* | *NA* | *Primary outcome: rate of intra hospital mortality between infants with cGS and sGS. Secondary outcomes were: length of hospital stay, duration of mechanical ventilation, start of enteral feedings, duration to full enteral feedings, duration of parenteral nutrition, number of infants with tube feedings on discharge, number of infants with parenteral nutrition on discharge, the rate of abdominal compartment syndrome, bowel obstruction/ileus, short bowel syndrome, necrotizing enterocolitis (NEC) and sepsis.* | *The most common sign was intestinal atresia (70.76%) followed by ischemia/perforation/necrosis (26.67%), volvulus and closing gastroschisis (each 8%).* |
| 1. i [Alshehri](https://pubmed.ncbi.nlm.nih.gov/?term=Alshehri+A&cauthor_id=24094951) J Pediatr Surg. 2013 Oct;48(10):2022-6. 5.2 | *Prospective national database* | *Of these 629, , 78 patients (12.4%) fulfilled the criteria for complex GS, including 57 patients (9.1%) with intestinal atresia, 44 (7%) with bowel necrosis, and 21 (3.3%) with bowel perforation.* | *Patients with intestinal atresia who underwent an intestinal operation during the first 21 days of life (EARLY GROUP)  14 patients* | *who underwent operations later (LATE GROUP) 13 patients* |  | *Early intestinal operations in patients with gastroschisis and intestinal atresia are not associated with increased complications and allow patients to receive and tolerate enteral feeding earlier.* | This presents some evidence that early establishment  of intestinal continuity in patients with GS, complicated  by atresia, allows for earlier initiation of enteral feeding and higher tolerance of enteral feeding during the first month of life. The practice does not appear to increase the common complications associated with complex gastroschisis, such as  line sepsis, bowel obstruction, and wound complications.  Given these results, early establishment of intestinal  continuity should be considered a safe option in select groups of patients with GS and atresia, where the procedure is deemed feasible. |

**Question:** Primary repair compared to primary reduction and delayed surgery for patients with complex gastroschisis

**Bibliography: Alsheri, 2013**

| **Certainty assessment** | | | | | | | **№ of patients** | | **Effect** | | **Certainty** | **Importance** |
| --- | --- | --- | --- | --- | --- | --- | --- | --- | --- | --- | --- | --- |
| **№ of studies** | **Study design** | **Risk of bias** | **Inconsistency** | **Indirectness** | **Imprecision** | **Other considerations** | **Primary repair** | **primary reduction and delayed surgery** | **Relative**  **(95% CI)** | **Absolute**  **(95% CI)** |  |  |
| **Mortality** | | | | | | | | | | | | |
| 1 | observational studies | serious^a^ | not serious | not serious | serious^b^ | none | 0/14 (0.0%) | 1/13 (7.7%) | **RR 0.3110**  (0.1380 to 7.0223) | **53 fewer per 1,000**  (from 66 fewer to 463 more) | ⨁◯◯◯  Very low | CRITICAL |
| **Infectious complications (sepsis or wound infection)** | | | | | | | | | | | | |
| 1 | observational studies | serious^a^ | not serious | not serious | serious^b^ | none | 2/14 (14.3%) | 4/13 (30.8%) | **RR 0.6964**  (0.1912 to 2.5368) | **93 fewer per 1,000**  (from 249 fewer to 473 more) | ⨁◯◯◯  Very low | CRITICAL |
| **Length of stay (no days in the hospital)** | | | | | | | | | | | | |
| 1 | observational studies | serious^a^ | not serious | not serious | serious^b^ | none | 123 | 144.2 | - | SMD **0.6314 SD lower**  (1.409 lower to 0.142 higher) | ⨁◯◯◯  Very low | CRITICAL |
| **TPN dependence at 28 days old** | | | | | | | | | | | | |
| 1 | observational studies | serious^a^ | not serious | not serious | serious^b^ | none | 4/14 (28.6%) | 9/13 (69.2%) | **RR 0.41**  (0.17 to 1.02) | **408 fewer per 1,000**  (from 575 fewer to 14 more) | ⨁◯◯◯  Very low | CRITICAL |

**CI:** confidence interval; **RR:** risk ratio; **SMD:** standardised mean difference

**Explanations**

a. Multivariate analysis missing

b. Probably due to small sample size

*Question 2.4*

**EVIDENCE TABLE PICO 6: Is intubation and mechanical ventilation during silo reduction in newborn with gastroschisis associated to better outcomes?**

Outcomes:

1. Mortality
2. Sedation
3. Infection: Sepsis or Bronchopneumonia
4. Days of Hospital Stay
5. Days to full enteral feeding
6. Bowel complications

| ***Reference*** | ***Study details*** | ***Patiënts and  inclusion*** | ***Intervention and study aim*** | ***Control*** | ***Follow-up*** | ***Outcomes of interest and effect*** | ***Notes:*** |
| --- | --- | --- | --- | --- | --- | --- | --- |
| *A. Owen et al.*  *J Ped Surg 2006* | Retrospective matched case-control  1990-2004    Sheffield Children´s Hospital (UK) | N= 65 consecutive patients  35 operative fascial closure  4 Bianchi  **26** Preformed silo, no general anesthesia (GA). **21 w/o ET/MV**  17 excluded because of urgent surgical conditions (bowel perforation, atresia, ward reduction, medical complications needing MV) | **21 No ET/MV**  preformed silo, no GA    Aim: Effectiveness of preformed silo without general anesthesia | 27 GA and operative closure | Unknown, follow up at least until enteral feeds. | 1. --- 2. --- 3. No differences in wound infections (0,49) or respiratory infections (p 0,41) 4. No differences (p 0,32) 5. No differences (p 0,26) 6. No differences in reoperating (0,41) or NEC (0,46) | Retrospective  Year 2006  Low nº patients |
| *L. Hong et al*  *Eur J Obst Gyn Repr Biol 2010* | Retrospective cohort  2004-2008 | N= 17 gastroschisis patients treated with preformed silo and No GA | 5 No ET/MV | 12 MV | from 1 to 51 months (range) | 1. (20 vs 8%) No differences because low nº 2. --- 3. No differences in wound infections (20 vs 25%) 4. No differences (median 16 vs 18 d) 5. No differences (median 15 vs 16d) 6. No differences (20 vs 16%) | Retrospective cohort  Year 2010  Low nº patients |

*Question 2.5*

**Question : What are the (un) favourable effects of non-absorbable patch versus biologic patch,  in neonates with gastroschisis if facial closure is not possible?**

| ***Reference*** | ***Study details*** | ***Patients and inclusion*** | ***Intervention and study aim*** | ***Contro*** | ***Follow-up*** | ***Outcomes of interest and effect*** | ***Notes:*** |
| --- | --- | --- | --- | --- | --- | --- | --- |
| *Stephania,(2020)*    *Biologic patch coverage for definitive management of giant gastroschisis defects* | Cases report | **n= 2**  Giant gastroschisis    Biological patch (Strattice ®)    Not amenable to primary closure |  |  | 1#: 17 months  2# 3yr 2 months | 1# the patient remains dependent on nasogastric tube feeds  2#prolonged ileus, sepsis, full gastrostomy tube feeding | complete, spontaneous closure of the fascial defects over  time, without the need for further operation |
| *Zmora, 2017*    *The biological prosthesis is a viable option for abdominal wall reconstruction in pediatric high risk defects* | Retrospective chart review    Single institution  2007-2015 | Mixed with other abdominal wall defects (n=23)    **N=11 gastroschisis**  (2 complicated cases: perforated bowel/ ileal atresia)    **Biological** mesh  (Alloderm™ was most commonly, Surgisis™, Strattice™, Flex-HD™ and Permacol®) | Demographic and clinical variables, technique and complications were described and compared between prosthesis types |  | Range 1.5months–72months    Mean 16.5 months | 5- no complications  1-death (renal failure)  2 skin separations  1-enterocutaneous fistula  1-recurrent hernia at follow-up.  1-subcutaneus infection, mesh removed  Most of the gastroschisis patients in this study had skin closure over the biologic patch.  83% of patients not developing hernia | No difference between mesh type;  use of a biological prosthesis may offer advantages over a synthetic mesh in paediatric high risk abdominal wall defects. |
| *Rahn,2007*    *Single-center 10-year experience in the management of anterior abdominal wall defects* | Retrospective data analysis    1995-2005 | Mixed with other abdominal wall defects (n=33)    N= 24 gastroschisis    **N=9 with patch**    3 single patches  6 two patches  **7 Synthetic (**Gore-Tex patch)  **5 biological** (4 dura, 1 bovine pericardium) | Present experience using biomaterials and evaluate the outcomes. |  | none | Intestine related complications in 8 (24%): relaparotomies in 6 patients due to mechanical ileus and 2 intestinal perforation) – no data which ones were with patches    Patch associated complications: 3 wound infections, 2 patch revisions    No biomaterial related complications | Gore-Tex well suited for large defects and Successful secondary abdominal wall reconstruction in all patients with patches  However, all patients required re-operation after 3 weeks for exchange of the patch |
| *Thanh, 2021*  *Successful secondary abdominal wall reconstruction in all patients with patches* | Case series | Mixed with other abdominal wall defects (n=3)    **N=1 large gastroschisis**    **Synthetic** Gore-Tex patch after silo reduction | Describe vacuum assisted closure |  | 24 months | Abdominal wall became infected, patch removed, vacuum assisted closure after 30 days.    On follow up – no facial defects, skin intact |  |
| *Paul M.Willis, 1995*  *Long-term results following repair of neonatal abdominal wall defects with Gore-Tex* | Retrospective data analysis    1985-1992 | Mixed with other abdominal wall defects (n=10)    **N=5** gastroschisis    **Synthetic** Gore-Tex patch | Report experience with Gore-Tex when facial closure is not possible.  Patch related complications, infection rates |  | Mean 2.2years  Range 13-63 months | All patients required graft removal because patch-related complications    No sepsis or enteric fistula    No ventral hernia afterwards    Gore-Tex closure is associated in all cases with either early or late exteriorization and/or infection. | Removal is easy and complication free.    Gore-Tex is useful synthetic material for closure but should be considered a temporary bridge to subsequent fascial closure |
| *Stringel, 1993*  *Large gastroschisis: Primary repair with gore-tex patch* | Case series | **N=4** gastroschisis    **Synthetic** Gore-Tex patch | Report experience with Gore-Tex when facial closure is not possible. |  | 4mo-3 years | 2 babies graft removal because of mild infection after 4 and 6 weeks.  One baby remained in the hospital for 4 weeks because of persistent vomiting caused by severe gastroesophageal reflux; Nissen fundoplication was done. | Gore-Tex patch closure is a safe and good alternative in large abdominal wall defects when primary fascial repair is not feasible |
| **Hussein Naji, 2014*  *Use of Surgisis for abdominal wall reconstruction in children with abdominal wall defects*  **additional article from reference list* | retrospective chart  2000 - 2010 | Mixed with other abdominal wall defects (n=13)    **N=2 gastroschisis**    **Biological patch** (Surgisis) | Data extracted included cause of defect, age at operation, possibility of skin coverage, recurrence, length of follow-up, and rate of wound infection. |  | median follow-up of 60 months (range, 10-90) | Immediate skin coverage was obtained in 1 of the 2 patients, whereas the patch in the other patient was left uncovered and allowed to granulate and epithelialize  Both patients had some postoperative wound erythema; however, neither child had recurrence of the defect. | Surgisis is moderately successful in the repair of paediatric abdominal wall defects. |
| **Gabriel, 2006*  *Management of complicated gastroschisis with porcine small intestinal submucosa and negative pressure wound therapy*  **additional article from reference list* | retrospective chart  2002-2005 | N=55 gastroschisis    **N=3 with patch**    **Biological patch** (Surgisis) | experience with visceral coverage in complicated cases of gastroschisis with porcine small intestinal submucosa (SIS) augmented by negative pressure wound therapy (NPWT). |  | 2# 6 months | 1# caecal resection due to stricture, neo-fascia removed  2#small umbilical hernia after 6 months (operated)  3#small hernia on follow up (conservatively) | SIS did not provide a complete and permanent fascial closure in all of our cases because 2 of our 3 patients developed ventral hernias. These hernias were, however, much smaller than the original defects  combination of SIS and NPWT can provide a safe and effective means of visceral coverage and, in some cases, definitive abdominal wall closure. |
| *Carachi R et al (1995)*  *Collagen-coated vicryl mesh. A new bioprothesis in pediatric surgical practice* | Retrospective analysis.    Inclusion period 1987-1992 | 28 patients with both thoracic wall defect after tumor surgery and patients with abdominal wall defects of various reasons, including **6 patients with gastroschisis**. | Two patients with skin closure, four without and one of these had a failed primary closure |  | 11 days to 45 months. | One died not related to procedure. | Oozing in one patient otherwise no “significant” postoperative complications.  One patient died of herpes hepatitis transferred from the mother |
| *Ikoma-N et al. (2013)*  *Technical note. Component separation technique with double layered biologic mesh for neonate with large gastroschisis.* | Case-report | N=1 | Onlay- and sublay mesh combined with component separation.  Mesh: Strattice® |  | unknown | Skin dehiscence due to poor blood supply in mobilised skin flaps | The double layered mesh is recommended, but there is no evidence for this. |
| *Chivukula-KK et al (2012)*  *Human aceelular dermal matrix for neonates with complex abdominal wall defects. Short- and long-term outcomes.* | Case serie  on 43 patients operated for both omphalocele and gastroschisis | N= 3 of gastroschisis received intervention. | Human Acellular dermal matrix (HADM) with secondary epithelisation. | - Conventional closure N= 24 | Two years | In two patients the mesh was removed because of infection and because of delayed primary closure | No ventral hernias  Significant longer length of stay at hospital, time to full nutrition and days on TPN compared to those with primary closure. |
| *Patti G et al. (1996)*  *Long-term results following repair of neonatal abdominal wall defects with Gore-tex* | Short communication on 6 patients with gastroschisis (2), omphalocele (2) and congenital diaphragmatic hernia (2). | 2 patients | Gore-Tex with primary skin closure | - None | Median 32 months for all patients, otherwise not specified. | In one patient the mesh was removed of cosmetic reasons. In the other patient, the mesh still was in situ. | None |
| *Rokitansky AM et al (1995)*  *Patchplasty with expanded polytetrafluroethylene and skinplasty for the closure of large congentinal abdominal wall defects.* | No patients included, just a description of methodology also recommend applied in gastroschisis |  | Gore-Tex |  |  | Traction method with delayed primary closure and skin closure including skin-flap mobilisation. |  |
| *Risby et al.*  *Congenital abdominal wall defects. Staged closure by dual mesh.* | Retrospective study with follow-up | 27 patients with gastrochisis and 7 with omphalocele | Gor-Tex (dual mesh®)  Staggen reduction technique. | None | 1-18 years | Closure obtained in  3 patients died not procedure-related.  Infectious complications 15%  Other mesh-related complications: 15%  At follow-up 10% were operated for a ventral hernia and 10% for ileus. | Removal of mesh within in 50% within 11 days and in 75% within 22 days. |

### Module 3

Question 3.1

| ***Reference*** | ***Study details*** | ***Patients and  inclusion*** | ***Intervention and study aim*** | - ***Control/comparison*** | - ***Follow-up*** | ***Outcomes of interest and effect*** | ***Notes:*** |
| --- | --- | --- | --- | --- | --- | --- | --- |
| *Aljahadali et al. 2013:*  *Effect of timing of enteral feeding on outcome in gastroschisis* | Observational,  Prospective data collection but retrospective analyzation    descriptive    CAPS-Net:  May 2005 and August 2011, a total of 630 liveborn babies with GS achieved “completed” status. Sixty babies were excluded due to missing essential fields, leaving 570 who compose the study cohort. | 570 cases (16% with “high risk” bowel injury). Group distribution:  Group 1 (≤7 days): n = 69 (12%)  Group 2 (8–14 days): n = 253 (44%)  Group 3 (15–21 days): n = 149 (26%)  Group 4 (N21 days): n = 98 (17%) | Purpose: analyze the effect of timing of first feeds on outcome.    Regression analysis of different milestones in relation to time to first enteral feeding.  Categorization of time to enteral feeding by weeks of life: Groups 1-4 |  |  | A  delay in enteral feeding by one day results in increases in TPN duration by 1.55 days and in LOS by 1.39 days.      There does not appear to be any advantage (and there may in fact be an outcome disadvantage from the perspective of TPN duration and LOS) to starting feeds within the first 7 days after abdominal closure> there may be a benefit to wait at least 7 days after closure to initiate feeds    Both TPN duration and LOS were significantly lower in Group 2 patients (28 ± 23 days; 39± 37 days) than Group 1 (38 ± 58 days; 55 ± 83 days), Group 3 (39 ± 25 days; 54± 34 days) or Group 4 patients (80 ± 70 days; 102 ± 71 days). From the perspective of infection, Groups 3 and 4 had significantly higher rates than Groups 1 (OR 2.4 and 4.8), which were not significantly different from each other | - mean duration of TPN and mean LOS were 44 ± 56 days and 112 ± 71 days  - surgical site infections (SSI) in 70 patients (12%) and 88 patients (15%) developed one or more episodes of culture-proven bacteraemia.  - 134 (24%) patients had either a SSI or at least one bacteremic episode  - risk attributes of these groups were comparable, except for a significantly greater proportion of high risk GPS patients within Group 4  - No intervention or control group.  - Very low evidence. |

*Question 3.2*

| ***Reference*** | ***Study details*** | ***Patiënts and  inclusion*** | ***Intervention and study aim*** | ***Control/vergelijking*** | ***Follow-up*** | ***Outcomes of interest and effect*** | ***Notes:*** |
| --- | --- | --- | --- | --- | --- | --- | --- |
| *Ma (2014)* | retrospective comparative study      data collection 2004-2013    1 center      Funding and conflict of interest:  no funding, no  disclosures | n=138  patients with gastroschisis , initial PICC    PICC placement:  40 pt(31%) LE  98 pt(69%) UE | complication rates of upper versus lower extremity PICCs in patients with gastroschisis    Catheter dwell time and complication rates (infiltration, phlebitis,occlusion, migration, infection and thrombosis) between the initial UE and LE PICCs were compared. |  |  | Complication rate:  LE: 20%  UE:3.4%  (OR 5;  95% CI: 1.2–21.5)    Infiltration:  LE: 11.5%  UE:1.4%  p = 0.025    phlebitis:  LE: 11.5%  UE: 0%; p<0.01    Sub analysis for SILO patients :  Complications in LE vs UE PICC :  OR 9.7 (1.9-49.7) | No multivariate analysis , but authors applied correction for type of treatment (SILO vs primary closure) and accounted for duration of PICC line insertion by calculating the risk in the first 5 days, as complications can increase over time. |

**Question: Upper extremity versus lower extremity PICC line**

**Bibliography: Ma et al, 2014**

| **Certainty assessment** | | | | | | | **№ of patients** | | **Effect** | | **Certainty** | **Importance** |
| --- | --- | --- | --- | --- | --- | --- | --- | --- | --- | --- | --- | --- |
| **№ of studies** | **Study design** | **Risk of bias** | **Inconsistency** | **Indirectness** | **Imprecision** | **Other considerations** | **Upper extremity picc line** | **Lower extremity picc line** | **Relative**  **(95% CI)** | **Absolute**  **(95% CI)** |  |  |
| **Complication rate (infection or phlebitis) within the first 5 days after placement of the line.** | | | | | | | | | | | | |
| 1 | observational studies | Very serious^a^ | not serious | not serious | not serious | none | 3/89 (3,4%) | 8/40 (20%) | RR 0.167  (0.050 to 0.600) | 167 fewer per 1,000  (from 190 fewer to 80 fewer) | ⨁◯◯◯  Very low | CRITICAL |

**CI:** confidence interval; **RR:** relative risk

**Explanations**

a. Although the study corrected for treatment type and time of catheter in place, the lack of a multivariate analysis, could be an issue for reliability of the results. The selection procedure of patients, even if drawn from the same sample, the placement of lines according to the nurse preference location could have led to selection bias.
