# Supplementary material for: European reference network for rare inherited congenital anomalies (ERNICA) evidence based guideline on the management of gastroschisis
Source: Orphanet J Rare Dis. 2024 Feb 12;19:60. doi: 10.1186/s13023-024-03062-8 (PMC10860293; doi:10.1186/s13023-024-03062-8)
Supplement: Supplementary file 3 — Additional file 3. Appendix C: Evidence to Decision tables. [file 13023_2024_3062_MOESM3_ESM.docx]

**Appendix C : Evidence to Decision tables.**

### Module 1

| Question 1.1a | |
| --- | --- |
| **Should preterm delivery vs. birth at term be used for woman expecting a child with gastroschisis ?** | |
| **Population:** | woman expecting a child with gastroschisis |
| **Intervention:** | preterm delivery |
| **Comparison:** | birth at term |
| **Main outcomes:** | Neonatal mortality and morbidity. |
| **Setting:** | HIgh resourse hospital |

Assessment

| Problem  Is the problem a priority? | | |
| --- | --- | --- |
| Judgement | Research evidence | Additional considerations |
| ○ No ○ Probably no ● Probably yes ○ Yes ○ Varies ○ Don't know | In some hospitals, preterm delivery (either elective vaginal delivery or cesarean section) is a standard procedure. Some believe that preterm birth will decrease the amount of complex cases. However, we are unsure if the expected benefits of this policy have any evidence base to support them. |  |
| Desirable Effects  How substantial are the desirable anticipated effects? | | |
| Judgement | Research evidence | Additional considerations |
| ○ Trivial o  Small ○ Moderate ○ Large ○ Varies ○ Don't know | Effect for mortality, severe GI-complications and time on TPN were all not significant and very low quality of evidence. Only for sepsis there was a significant effect, indicating a decrease (OR 0.43) in the risk of neonatal sepsis after elective preterm delivery (<37wks) but this was only based on 3 observational studies which inclines a high risk of bias.   Summarizing all the available evidence, there is no evidence to show that preterm birth has any beneficial effects on the selected outcomes. |  |
| Undesirable Effects  How substantial are the undesirable anticipated effects? | | |
| Judgement | Research evidence | Additional considerations |
| ○ Large ○ Moderate ○ Small ○ Trivial ○ Varies ● Don't know | No clear negative effects of the intervention were found looking at the selected outcomes. | The guideline panel discussed preterm delivery as everything before 37 weeks of gestation. The term for elective preterm delivery of a child with gastroschisis will often be at 36-37 weeks of gestation. The effects of moderate or late preterm birth on neurodevelopmental outcome are not reported as evaluated outcomes for this question, but should be considered. The risks of neurodevelopmental problems are reported to be double in moderate to late preterm babies compared to term born peers.  Reference: Johnson et al. (2015) |
| Certainty of evidence  What is the overall certainty of the evidence of effects? | | |
| Judgement | Research evidence | Additional considerations |
| ● Very low ○ Low ○ Moderate ○ High ○ No included studies | The included studies only had one RCT and this RCT only covered two of the outcomes (mortality and severe GI complications). The rest of the meta-analysis was comprised of prospective or retrospective observational studies, comparing groups through either a cutoff of 37 wks gestation at birth or elective preterm delivery vs elective term delivery. There was a high risk of bias according to the NOS scale and some outcomes were rated as indirect evidence. |  |
| Values  Is there important uncertainty about or variability in how much people value the main outcomes? | | |
| Judgement | Research evidence | Additional considerations |
| ○ Important uncertainty or variability ○ Possibly important uncertainty or variability ○ Probably no important uncertainty or variability ○ No important uncertainty or variability | All the main outcomes are perceived as the most important markers of wellbeing in the child with gastroschisis. | Not relevant for the main outcomes on the baby, but for the mother, there could be cultural differences in the importance of choices around birth. In some European countries the option to deliver as ‘natural as possible’ will be much more important to the parents than in some other countries where birth is already perceived much more as a medical intervention. |
| Balance of effects  Does the balance between desirable and undesirable effects favor the intervention or the comparison? | | |
| Judgement | Research evidence | Additional considerations |
| ○ Favors the comparison ○ Probably favors the comparison ○ Does not favor either the intervention or the comparison ○ Probably favors the intervention ○ Favors the intervention ○ Varies ○ Don't know | As elective preterm delivery can only decrease risk in neonatal sepsis with quality of evidence low++, it looks like preterm delivery can cause more complications from prematurity for the baby. There is no evidence to recommend an active intervention, however, we don’t have any information about babies with complications or suspected complex gastroschisis (Landish et al., 2017)  The panel feels that even if there is no evidence, it feels uncomfortable to let the pregnancies continue further than 39 weeks because of the increase in risks for intra uterine fetal demise.  Overall, the evidence of certainty is low, but we have no indications that preterm birth has any advantages and we do have indications that preterm birth has possible neurodevelopmental disadvantages. Therefore, the panel sees no basis to recommend or suggest the invasive option of preterm delivery. | The effects of moderate or late preterm birth on neurodevelopmental outcome (double risk compared to term born peers) are not reported (Johnson et al., 2015) |
| Resources required  How large are the resource requirements (costs)? | | |
| Judgement | Research evidence | Additional considerations |
| ○ Large costs ○ Moderate costs ○ Negligible costs and savings ○ Moderate savings ○ Large savings ○ Varies ○ Don't know | Delivery before or after 37 weeks will not change the required resources | It is more the context of the whole treatment for gastroschisis that will determine the cost, where in general less complications at birth, will lead to less costs. |
| Certainty of evidence of required resources  What is the certainty of the evidence of resource requirements (costs)? | | |
| Judgement | Research evidence | Additional considerations |
| ○ Very low ○ Low ○ Moderate ○ High ○ No included studies |  |  |
| Cost effectiveness  Does the cost-effectiveness of the intervention favor the intervention or the comparison? | | |
| Judgement | Research evidence | Additional considerations |
| ○ Favors the comparison ○ Probably favors the comparison ○ Does not favor either the intervention or the comparison ○ Probably favors the intervention ○ Favors the intervention ○ Varies ● No included studies |  |  |
| Equity  What would be the impact on health equity? | | |
| Judgement | Research evidence | Additional considerations |
| ○ Reduced ○ Probably reduced ○ Probably no impact ○ Probably increased ○ Increased ○ Varies ○ Don't know | If an assumption is made that neurodevelopmental outcome is impaired in elective moderate-late preterm delivery, this effect will be worse in low socio-economic background and therefore reduce health equity |  |
| Acceptability  Is the intervention acceptable to key stakeholders? | | |
| Judgement | Research evidence | Additional considerations |
| ○ No ○ Probably no ○ Probably yes ○ Yes ○ Varies ○ Don't know | Preterm delivery is common for gastroschisis babies in many European countries. Recommending it as a first choice could be acceptable but not without a sufficient evidence base. |  |
| Feasibility  Is the intervention feasible to implement? | | |
| Judgement | Research evidence | Additional considerations |
| ○ No ○ Probably no ● Probably yes ○ Yes ○ Varies ○ Don't know |  | It is feasible but does not seem advisable. |

Summary of judgements

|  | **Judgement** | | | | | | |
| --- | --- | --- | --- | --- | --- | --- | --- |
| **Problem** | No | Probably no | **Probably yes** | Yes |  | Varies | Don't know |
| **Desirable Effects** | **Trivial** | Small | Moderate | Large |  | Varies | Don't know |
| **Undesirable Effects** | Large | Moderate | Small | Trivial |  | Varies | **Don't know** |
| **Certainty of evidence** | **Very low** | Low | Moderate | High |  |  | No included studies |
| **Values** | Important uncertainty or variability | Possibly important uncertainty or variability | **Probably no important uncertainty or variability** | No important uncertainty or variability |  |  |  |
| **Balance of effects** | Favors the comparison | **Probably favors the comparison** | Does not favor either the intervention or the comparison | Probably favors the intervention | Favors the intervention | Varies | Don't know |
| **Resources required** | Large costs | Moderate costs | **Negligible costs and savings** | Moderate savings | Large savings | Varies | Don't know |
| **Certainty of evidence of required resources** | Very low | Low | Moderate | High |  |  | **No included studies** |
| **Cost effectiveness** | Favors the comparison | Probably favors the comparison | Does not favor either the intervention or the comparison | Probably favors the intervention | Favors the intervention | Varies | **No included studies** |
| **Equity** | Reduced | Probably reduced | Probably no impact | Probably increased | Increased | Varies | Don't know |
| **Acceptability** | No | Probably no | **Probably yes** | Yes |  | Varies | Don't know |
| **Feasibility** | No | Probably no | **Probably yes** | Yes |  | Varies | Don't know |

Type of recommendation

| Strong recommendation against the intervention | **Conditional recommendation against the intervention** | Conditional recommendation for either the intervention or the comparison | Conditional recommendation for the intervention | Strong recommendation for the intervention |
| --- | --- | --- | --- | --- |
| ○ | **●** | ○ | ○ | ○ |

Conclusions

| Recommendation |
| --- |
| **The panel suggests vaginal birth between 37+0 and 39+0 weeks in children with uncomplicated gastroschisis. ***  *Linked to recommendation chapter 1b. |
|  |
| Justification |
| **Overall justification** The panel considers full term delivery to be safe option and there is no evidence that suggests advantages of delivery before 37 weeks.  **Detailed justification**  *Balance of benefits and harms*  We have no indications that preterm birth has any advantages and we do have indications that preterm birth has possible neurodevelopmental disadvantages. Therefore the panel sees no basis to recommend or suggest the invasive option of preterm delivery, unless any other obstetrical complications occur.  *Certainty of evidence*  More data is needed from prospective data or RCTs, measuring other key outcomes such as neurodevelopmental outcomes as well as the differences between patients with (suspected) complex gastroschisis and uncomplicated gastroschisis. |

| Subgroup considerations |
| --- |
| For the subgroup with suspected complex gastroschisis preterm delivery, can be considered. If there are signs of complicated gastroschisis (for example signs of peritonitis or anamnios), the risks of awaiting birth outweigh the benefits of birth at term. In particular, dilated bowel (extra-abdominal or intra-abdominal, see chapter 2) are associated with complex gastroschisis. |

| Research priorities |
| --- |
| Prospective studies of induction of preterm delivery in children with simple or suspected complex gastroschisis, to see if delivering children gastroschisis early does indeed lead to better outcomes, including long term and neurodevelopmental outcomes. |

References

Landisch RM, Yin Z, Christensen M, Szabo A, Wagner AJ. Outcomes of gastroschisis early delivery: A systematic review and meta-analysis. J Pediatr Surg. 2017 Dec;52(12):1962-1971. doi: 10.1016/j.jpedsurg.2017.08.068. Epub 2017 Sep 7. PMID: 28947324

Johnson, S., Evans, T.A., Draper, E.S., Field, D.J., Manktelow, B.N., Marlow, N., Matthews, R., Petrou, S., Seaton, S.E., Smith, L.K., Boyle, E.M., 2015.

Neurodevelopmental outcomes following late and moderate prematurity: a population-based cohort study. Archives of Disease in Childhood - Fetal and Neonatal Edition 100, F301–F308.. doi:10.1136/archdischild-2014-307684

| Question 1.1b | |
| --- | --- |
| **Should cesarean section vs. vaginal birth be used for woman expecting a child with gastroschisis ?** | |
| **Population:** | woman expecting a child with gastroschisis |
| **Intervention:** | cesarean section |
| **Comparison:** | vaginal birth |
| **Main outcomes:** | Neonatal mortality and morbidity. |
| **Setting:** | HIgh resourse hospital |

Assessment

| Problem  Is the problem a priority? | | |
| --- | --- | --- |
| Judgement | Research evidence | Additional considerations |
| ○ No ○ Probably no ○ Probably yes ○ Yes ○ Varies ○ Don't know | In some hospitals a cesarean section is a standard procedure for the birth of gastroschisis babies. Some advantages in the organization of care have been mentioned to advocate for a planned birth. However, we are unsure if the expected benefits of this policy have any evidence base to support them if we look at neonatal outcomes for gastroschisis. |  |
| Desirable Effects  How substantial are the desirable anticipated effects? | | |
| Judgement | Research evidence | Additional considerations |
| ● Trivial ○ Small ○ Moderate ○ Large ○ Varies ○ Don't know | No significant effect was found for any of the outcomes (mortality, sepsis, gastro-intestinal complications, time on PN) |  |
| Undesirable Effects  How substantial are the undesirable anticipated effects? | | |
| Judgement | Research evidence | Additional considerations |
| ○ Large ○ Moderate ○ Small ○ Trivial ○ Varies ○ Don't know | No clear undesirable effects for the neonate were found. | C-section delivery carries some morbidity and risks, primarily to the mother, compared to vaginal birth. Caesarean sections can cause significant and sometimes permanent complications, disability or death particularly in settings that lack the facilities and/or capacity to properly conduct safe surgery and treat surgical complications. Cesarean sections should ideally only be undertaken when medically necessary (WHO, 2015). Some studies mention disadvantages for the neonate as well, such as a higher risk of respiratory tract infections (Słabuszewska-Jóźwiak et al., 2020) In the evaluated review, only neonatal outcomes were measured. Avoiding a c-section may have a positive effect on the mother and we did not find any neonatal harms associated with vaginal delivery. |
| Certainty of evidence  What is the overall certainty of the evidence of effects? | | |
| Judgement | Research evidence | Additional considerations |
| ● Very low ○ Low ○ Moderate ○ High ○ No included studies | Evidence from observational studies only. For all outcomes, imprecision was detected and some concerns for methodological bias. |  |
| Values  Is there important uncertainty about or variability in how much people value the main outcomes? | | |
| Judgement | Research evidence | Additional considerations |
| ○ Important uncertainty or variability ○ Possibly important uncertainty or variability ○ Probably no important uncertainty or variability ○ No important uncertainty or variability |  | Outcomes are focussed on advantages of the baby, (dis)advantages of the mother should be considered. |
| Balance of effects  Does the balance between desirable and undesirable effects favor the intervention or the comparison? | | |
| Judgement | Research evidence | Additional considerations |
| ○ Favors the comparison ○ Probably favors the comparison ○  Does not favor either the intervention or the comparison ○ Probably favors the intervention ○ Favors the intervention ○ Varies ○ Don't know | There is no difference in outcomes, meaning that there is no evidence that cesarean section (the more invasive option) has any advantages over vaginal birth. The panel considers vaginal birth to be a safe option. |  |
| Resources required  How large are the resource requirements (costs)? | | |
| Judgement | Research evidence | Additional considerations |
| ○ Large costs ○ Moderate costs ○ Negligible costs and savings ○ Moderate savings ○ Large savings ○ Varies ○ Don't know | C-Section would increase costs in most European countries. These are costs in the care for the mother, but overall, the effect will be small compared to the costs of the treatment of the child. If the delivery occurs at night or during the weekend, the cost of mobilizing the resources for the newborn may outweigh the savings of the vaginal delivery. |  |
| Certainty of evidence of required resources  What is the certainty of the evidence of resource requirements (costs)? | | |
| Judgement | Research evidence | Additional considerations |
| ○ Very low ○ Low ○ Moderate ○ High ● No included studies |  |  |
| Cost effectiveness  Does the cost-effectiveness of the intervention favor the intervention or the comparison? | | |
| Judgement | Research evidence | Additional considerations |
| ○ Favors the comparison ○ Probably favors the comparison ○ Does not favor either the intervention or the comparison ○ Probably favors the intervention ○ Favors the intervention ○ Varies ○ No included studies |  |  |
| Equity  What would be the impact on health equity? | | |
| Judgement | Research evidence | Additional considerations |
| ○ Reduced ○ Probably reduced ○ Probably no impact ○ Probably increased ○ Increased ○ Varies ○ Don't know |  |  |
| Acceptability  Is the intervention acceptable to key stakeholders? | | |
| Judgement | Research evidence | Additional considerations |
| ○ No ○ Probably no ○ Probably yes ○ Yes ○ Varies ○ Don't know | There will be differences in acceptability around Europe as the mode of delivery is influenced by cultural differences. Offering cesarean section as primary option will probably not be acceptable based on the current evidence. |  |
| Feasibility  Is the intervention feasible to implement? | | |
| Judgement | Research evidence | Additional considerations |
| ○ No ○ Probably no ● Probably yes ○ Yes ○ Varies ○ Don't know |  |  |

Summary of judgements

|  | **Judgement** | | | | | | |
| --- | --- | --- | --- | --- | --- | --- | --- |
| **Problem** | No | Probably no | Probably yes | Yes |  | Varies | Don't know |
| **Desirable Effects** | **Trivial** | Small | Moderate | Large |  | Varies | Don't know |
| **Undesirable Effects** | Large | Moderate | **Small** | Trivial |  | Varies | Don't know |
| **Certainty of evidence** | **Very low** | Low | Moderate | High |  |  | No included studies |
| **Values** | Important uncertainty or variability | Possibly important uncertainty or variability | **Probably no important uncertainty or variability** | No important uncertainty or variability |  |  |  |
| **Balance of effects** | Favors the comparison | **Probably favors the comparison** | Does not favor either the intervention or the comparison | Probably favors the intervention | Favors the intervention | Varies | Don't know |
| **Resources required** | Large costs | Moderate costs | **Negligible costs and savings** | Moderate savings | Large savings | Varies | Don't know |
| **Certainty of evidence of required resources** | Very low | Low | Moderate | High |  |  | **No included studies** |
| **Cost effectiveness** | Favors the comparison | Probably favors the comparison | Does not favor either the intervention or the comparison | Probably favors the intervention | Favors the intervention | Varies | **No included studies** |
| **Equity** | Reduced | Probably reduced | **Probably no impact** | Probably increased | Increased | Varies | Don't know |
| **Acceptability** | No | **Probably no** | Probably yes | Yes |  | Varies | Don't know |
| **Feasibility** | No | Probably no | **Probably yes** | Yes |  | Varies | Don't know |

Type of recommendation

| Strong recommendation against the intervention | **Conditional recommendation against the intervention** | Conditional recommendation for either the intervention or the comparison | Conditional recommendation for the intervention | Strong recommendation for the intervention |
| --- | --- | --- | --- | --- |
| ○ | ● | ○ | ○ | ○ |

Conclusions

| Recommendation |
| --- |
| **The panel suggests vaginal birth between 37+0 and 39+0 weeks in children with uncomplicated gastroschisis. ***  * Linked to recommendation 1a. |
|  |
| Justification |
| **Overall justification** The panel considers vaginal delivery to be safe option and there is no evidence that suggests advantages of delivery via cesarean section.  **Detailed justification**  *Balance of benefits and harms*  We have no indications that cesarean section birth has any advantages and cesarean section holds some disadvantages to the mother. Therefore, the panel sees no basis to recommend or suggest the invasive option of cesarean delivery, unless any there are any obstetric reasons for cesarean section.  *Certainty of evidence*  More data is needed from prospective data or RCTs, measuring other key outcomes such as neurodevelopmental outcomes for the neonates and effects for the mothers as well as the differences between patients with (suspected) complex gastroschisis and uncomplicated gastroschisis. |
|  |

| Subgroup considerations |
| --- |
| If there are signs of complicated gastroschisis, the risks of awaiting birth may outweigh the benefits of induced birth or cesarean section. In particular, dilated bowels (extra-abdominal or intra-abdominal, see chapter 2) are associated withcomplex gastroschisis. For this subgroup with suspected complex gastroschisis, (preterm) delivery (vaginal or with cesarean section), can be considered. |

| Research priorities |
| --- |

Research prospectively comparing cesarean delivery and vaginal birth in children with suspected complex gastroschisis, to determine whether delivering children with suspected complex gastroschisis via cesarean section leads to better outcomes.

|  | |
| --- | --- |
| Question 1.2 | |
| **Should ultrasound markers be used for prognosis in gastroschisis ?** | |
| **Population:** | Women pregnant with a fetus with gastroschisis |
| **Intervention:** | Prenatal measurement of Intra-abdominal bowel dilation, extra-abdominal bowel dilation and mesenteric artery flows with ultrasound |
| **Comparison:** |  |
| **Main outcomes:** | Predictive value for complex gastroschisis. |
| **Setting:** | Modern high resource hospital. |

Assessment

| Problem  Is the problem a priority? | | |
| --- | --- | --- |
| Judgement | Research evidence | Additional considerations |
| ○ No ○ Probably no ○ Probably yes ● Yes ○ Varies ○ Don't know | Prenatal identification of complex gastroschisis would improve parental counseling and perinatal planning. It still remains unclear which of these ultrasonographic findings can predict a more complex course of disease |  |
| Desirable Effects  How substantial are the desirable anticipated effects? | | |
| Judgement | Research evidence | Additional considerations |
| ○ Trivial ● Small ○ Moderate ○ Large ○ Varies ○ Don't know | Using intra-abdominal bowel and extra-abdominal bowel dilation as prognostic factors would be helpful to predict complex gastroschisis and longer hospital stay. Predicting these in an early stage might have desirable effects such as better information provision to parents and perinatal care planning. However, tangible desirable effects only exist if detection of divergent ultrasound parameters would change the course of treatment to improve outcomes. So far, no evidence suggests an intervention for cases where signs of complex gastroschisis are detected.  One study showed indications of an association between higher neonatal mortality and perturbed mesentery artery flows. The definition of ‘perturbed’ was not clearly described and could not be found in other studies. |  |
| Undesirable Effects  How substantial are the undesirable anticipated effects? | | |
| Judgement | Research evidence | Additional considerations |
| ○ Large ○ Moderate ○ Small ● Trivial ○ Varies ○ Don't know | If ultrasound markers are used to predict the course of the disease, frequent hospital visits for ultrasounds are a consequence as the condition of a fetus cannot be determined based on one or two measurements. The panel perceives this as a trivial undesirable effect because most patients do not report that frequent visits are bothersome. |  |
| Certainty of evidence  What is the overall certainty of the evidence of effects? | | |
| Judgement | Research evidence | Additional considerations |
| ○ Very low ○ Low ● Moderate ○ High ○ No included studies | Moderate levels of evidence for IABD and EABD from a meta-analysis (Sun et al., 2021; D’Antonio et al., 2015). Dilation of the bowel, especially during the second trimester, is associated with a more complex course of the condition. However, the definition for both IABD and EABD was inconsistent and varied in most of the studies, therefore the quality of evidence was graded moderate. In one observational study, the prognostic value of mesenteric artery flows was explored, however no definition was given for how perturbed mesenteric artery flows should be interpreted. |  |
| Values  Is there important uncertainty about or variability in how much people value the main outcomes? | | |
| Judgement | Research evidence | Additional considerations |
| ○ Important uncertainty or variability ○ Possibly important uncertainty or variability ○ Probably no important uncertainty or variability ○ No important uncertainty or variability |  |  |
| Balance of effects  Does the balance between desirable and undesirable effects favor the intervention or the comparison? | | |
| Judgement | Research evidence | Additional considerations |
| ○ Favors the comparison ○ Probably favors the comparison ○ Does not favor either the intervention or the comparison ● Probably favors the intervention ○ Favors the intervention ○ Varies ○ Don't know | With support, counselling and careful prenatal management, fetuses with gastroschisis and their parents benefit from a prenatal diagnosis. Especially during the third trimester, intra-abdominal and extra-abdominal bowel diameter should be monitored to identify fetuses at risk for complex gastroschisis. However, ongoing research to set clear cut-offs on bowel diameter should be performed. |  |
| Resources required  How large are the resource requirements (costs)? | | |
| Judgement | Research evidence | Additional considerations |
| ○ Large costs ○ Moderate costs ● Negligible costs and savings ○ Moderate savings ○ Large savings ○ Varies ○ Don't know | In most European countries there are already prenatal screening programs. Looking at 2 extra or less parameters would not change the costs. | Only if prenatal surveillance can identify fetuses at risk for complex gastroschisis early, and management options would be available to prevent further development of complexity, than the ultrasound screenings have a preventive function and could possibly save costs in the treatment of the neonate. |
| Certainty of evidence of required resources  What is the certainty of the evidence of resource requirements (costs)? | | |
| Judgement | Research evidence | Additional considerations |
| ○ Very low ○ Low ○ Moderate ○ High ● No included studies |  |  |
| Cost effectiveness  Does the cost-effectiveness of the intervention favor the intervention or the comparison? | | |
| Judgement | Research evidence | Additional considerations |
| ○ Favors the comparison ○ Probably favors the comparison ○ Does not favor either the intervention or the comparison ○ Probably favors the intervention ○ Favors the intervention ○ Varies ● No included studies |  |  |
| Equity  What would be the impact on health equity? | | |
| Judgement | Research evidence | Additional considerations |
| ○ Reduced ○ Probably reduced ○ Probably no impact ● Probably increased ○ Increased ○ Varies ○ Don't know | If risk factors for complex gastroschisis are indentified, postnatal treatment can be planned and parents can be accurately informed.  The panel agrees that every child should be born in the best possible condition. If a positive US test for IABD prompts measures to support the first moments of life, then ultrasound measures could increase equity for all GS patients. |  |
| Acceptability  Is the intervention acceptable to key stakeholders? | | |
| Judgement | Research evidence | Additional considerations |
| ○ No ○ Probably no ○ Probably yes ○ Yes ○ Varies ○ Don't know | The rationale of ultrasound screening of fetuses with gastroschisis is to identify fetuses at risk for adverse outcome to optimize perinatal planning and counseling of future parents expecting a child with gastroschisis. The panel therefore does not expect problems in the acceptability of these recommendations. |  |
| Feasibility  Is the intervention feasible to implement? | | |
| Judgement | Research evidence | Additional considerations |
| ○ No ○ Probably no ○ Probably yes ● Yes ○ Varies ○ Don't know | Prenatal ultrasound should already be available in every European member state and is a part of daily clinical practice. Therefore, we don't expect problems related to the implementation of the recommendations. |  |

Summary of judgements

|  | **Judgement** | | | | | | |
| --- | --- | --- | --- | --- | --- | --- | --- |
| **Problem** | No | Probably no | Probably yes | **Yes** |  | Varies | Don't know |
| **Desirable Effects** | Trivial | Small | **Moderate** | Large |  | Varies | Don't know |
| **Undesirable Effects** | Large | Moderate | Small | **Trivial** |  | Varies | Don't know |
| **Certainty of evidence** | Very low | Low | **Moderate** | High |  |  | No included studies |
| **Values** | Important uncertainty or variability | Possibly important uncertainty or variability | Probably no important uncertainty or variability | No important uncertainty or variability |  |  |  |
| **Balance of effects** | Favors the comparison | Probably favors the comparison | Does not favor either the intervention or the comparison | **Probably favors the intervention** | Favors the intervention | Varies | Don't know |
| **Resources required** | Large costs | Moderate costs | **Negligible costs and savings** | Moderate savings | Large savings | Varies | Don't know |
| **Certainty of evidence of required resources** | Very low | Low | Moderate | High |  |  | **No included studies** |
| **Cost effectiveness** | Favors the comparison | Probably favors the comparison | Does not favor either the intervention or the comparison | Probably favors the intervention | Favors the intervention | Varies | **No included studies** |
| **Equity** | Reduced | Probably reduced | Probably no impact | **Probably increased** | Increased | Varies | Don't know |
| **Acceptability** | No | Probably no | **Probably yes** | Yes |  | Varies | Don't know |
| **Feasibility** | No | Probably no | **Probably yes** | Yes |  | Varies | Don't know |

Type of recommendation

| Strong recommendation against the intervention | Conditional recommendation against the intervention | Conditional recommendation for either the intervention or the comparison | **Conditional recommendation for the intervention** | Strong recommendation for the intervention |
| --- | --- | --- | --- | --- |
| ○ | ○ | ○ | **●** | ○ |

Conclusions

| Recommendation |
| --- |
| The panel suggests interpreting IABD and EABD as predictors for complex gastroschisis.  The panel recommends evaluating the fetus with gastroschisis based on a complete image of different ultrasound parameters combined. It would, therefore, be useful to evaluate bowel thickness, gastric dilation, herniation of the stomach and/or bladder through the abdominal wall defect, presence of polyhydramnios, fetal growth parameters, fetal movement and size of the abdominal wall defect.  The panel suggests to refrain from using altered mesenteric artery flow as a prognostic factor for complex gastroschisis. |
|  |
| Justification |
| **Overall justification**  We have moderate quality of evidence suggesting increased risks for complex gastroschisis in case of IABD and/or EABD. However, in this chapter only a preselected group of ultrasound markers were reviewed, but it remains important to consider the whole picture. It would, therefore, be useful to evaluate other ultrasound parameters such as bowel thickness, gastric dilation, herniation of the stomach and/or bladder through the abdominal wall defect, presence of polyhydramnios, fetal growth parameters, fetal movement, and size of the abdominal wall defect.  **Detailed justification**  *Balance of benefits and harms*  The harms of using these ultrasound parameters are trivial and we are moderately confident that IABD and EABD are predictors for complex gastroschisis. |

| Subgroup considerations |
| --- |
| For very large dilation there are even higher concerns for complex gastroschisis although no clear cut-off could be defined from the analyzed literature. |

| Research priorities |
| --- |
| Future research should focus on defining clear cut-off values for intra-abdominal dilated bowel, extra abdominal dilated bowel and bladder position, especially during third trimester. In the revision of the datasets for the gastroschisis registry, these knowledge gaps should be taken into account. |

**References Summary**R. C. Sun, K. Hessami, E. Krispin, M. Pammi, S. Mostafaei, L. Joyeux, J. Deprest, S. Keswani, T. C. Lee, A. King, M. A. Belfort and A. A. Shamshirsaz. Prenatal ultrasonographic markers for prediction of complex gastroschisis and adverse perinatal outcomes: a systematic review and meta-analysis. Arch Dis Child Fetal Neonatal Ed 2022; 107: 371-379.

F. D'Antonio, C. Virgone, G. Rizzo, A. Khalil, D. Baud, T. E. Cohen-Overbeek, M. Kuleva, L. J. Salomon, M. E. Flacco, L. Manzoli and S. Giuliani. Prenatal Risk Factors and Outcomes in Gastroschisis: A Meta-Analysis. Pediatrics 2015; 136: e159-169.

### Module 2

| Question 2.1 | |
| --- | --- |
| **Should Bianchi’s Approach vs. Primary surgical closure be used for closure of the abdominal wall in gastroschisis?** | |
| **Population:** | closure of the abdominal wall in patients with simple gastroschisis |
| **Intervention:** | Bianchi’s approach |
| **Comparison:** | Primary closure |
| **Main outcomes:** | Mortality, Sepsis, major GI-complications , cosmetic result |
| **Setting:** | Modern high resource hospital |

Assessment

| Problem  Is the problem a priority? | | |
| --- | --- | --- |
| Judgement | Research evidence | Additional considerations |
| ○ No ○ Probably no ● Probably yes ○ Yes ○ Varies ○ Don't know | A European Survey from 2011 disclosed that 75 – 80% vs 20-25% of surgeons favored primary operative closure to Bianchi approach as the first line of management of gastroschisis. (1) It is however unclear what method has best outcomes. |  |
| Desirable Effects  How substantial are the desirable anticipated effects? | | |
| Judgement | Research evidence | Additional considerations |
| ● Trivial ○  Small ○ Moderate ○ Large ○ Varies ○ Don't know | For outcomes mortality, septicemia, bowel ischemia, TPN >60 days, hospital length of stay no significant difference was observed (3-5). | In Bianchi’s approach, an additional positive effect could be avoidance of general anesthesia and, maybe, intubation. As there are indications for neurotoxic effect of general anesthetics in neonates, avoiding anesthesia could be a long term benefit. Avoiding intubation could mean avoiding the risk of endotracheal tube complications.  There was no evidence for cosmetic result, but experts think that if sterile strips are used for closure after Bianchi, there is probably a cosmetic benefit. |
| Undesirable Effects  How substantial are the undesirable anticipated effects? | | |
| Judgement | Research evidence | Additional considerations |
| ○ Large ○ Moderate ○ Small ○ Trivial ○ Varies ● Don't know |  | The neurocognitive effect of general anesthesia was not considered as undesirable outcome in any of the included studies.  Bianchi's approach poaches a higher risk on later hernia formation. This was not described in outcome measures of analyzed studies, but Youssef et al. (2016) found a significant increase in hernia formation after sutureless closure (41% vs. 19%, p=0.0001). |
| Certainty of evidence  What is the overall certainty of the evidence of effects? | | |
| Judgement | Research evidence | Additional considerations |
| ● Very low ○ Low ○ Moderate ○ High ○ No included studies | The quality of evidence of the available literature is very low. None of the studies explicated based on what criteria patients were selected for primary closure or Bianchi’s approach. This initiates a serious risk of selection bias. Because all evidence comes from retrospective observational studies, confounding and selection bias result in the need for careful interpretations of conclusions. |  |
| Values  Is there important uncertainty about or variability in how much people value the main outcomes? | | |
| Judgement | Research evidence | Additional considerations |
| ○ Important uncertainty or variability ○ Possibly important uncertainty or variability ● Probably no important uncertainty or variability  ○ No important uncertainty or variability | The importance of the main outcomes (mortality, sepsis, and major GI-complications) is clear to all stakeholders | Patients might place higher value on the long-term cosmetic results and for parents, comfort and pain in the neonate during treatment is important. Neither outcomes were evaluated in the current available studies. |
| Balance of effects  Does the balance between desirable and undesirable effects favor the intervention or the comparison? | | |
| Judgement | Research evidence | Additional considerations |
| ○ Favors the comparison ○ Probably favors the comparison ● Does not favor either the intervention or the comparison ○ Probably favors the intervention ○ Favors the intervention ○ Varies ○ Don't know | For the main outcomes, there are no indications one procedure provides better results than the other. | The panel believes that the cosmetic results is better in Bianchi’s approach combined with sutureless closure. and that it could be beneficial for the neonate to avoid general anesthesia and intubation. The risk of herniation however could be increased if sutureless closure is achieved. |
| Resources required  How large are the resource requirements (costs)? | | |
| Judgement | Research evidence | Additional considerations |
| ○ Large costs ○ Moderate costs ○ Negligible costs and savings ● Moderate savings ○ Large savings ○ Varies ○ Don't know | For a surgical intervention like primary closure, a fully equipped team is necessary. If you can avoid a surgical intervention with using Bianchi there will probably be savings on the surgical team. For cot-site reduction you only need the time and patience of one surgeon alone. |  |
| Certainty of evidence of required resources  What is the certainty of the evidence of resource requirements (costs)? | | |
| Judgement | Research evidence | Additional considerations |
| ○ Very low ○ Low ○ Moderate ○ High ● No included studies |  |  |
| Cost effectiveness  Does the cost-effectiveness of the intervention favor the intervention or the comparison? | | |
| Judgement | Research evidence | Additional considerations |
| ○ Favors the comparison ○ Probably favors the comparison ○ Does not favor either the intervention or the comparison ○ Probably favors the intervention ○ Favors the intervention ○ Varies ● No included studies |  |  |
| Equity  What would be the impact on health equity? | | |
| Judgement | Research evidence | Additional considerations |
| ○ Reduced ○ Probably reduced ● Probably no impact ○ Probably increased ○ Increased ○ Varies ○ Don't know |  |  |
| Acceptability  Is the intervention acceptable to key stakeholders? | | |
| Judgement | Research evidence | Additional considerations |
| ○ No ○ Probably no ● Probably yes ○ Yes ○ Varies ○ Don't know | Implementing Bianchi’s approach as primary choice would not be acceptable, but the analysis shows that it is feasible in selected cases and can have some benefits. Therefore, considering it as an option next to primary closure in these cases is probably acceptable. |  |
| Feasibility  Is the intervention feasible to implement? | | |
| Judgement | Research evidence | Additional considerations |
| ○ No ○ Probably no ○ Probably yes ○ Yes ● Varies ○ Don't know | For successful implementation of Bianchi’s approach as a treatment option you need a specialized and experienced team. Not all European centers have this experience, and it is hard to obtain experience if you only have two Gastroschisis cases a year. Since not all patients are suitable for Bianchi’s, getting the exposure to build experience and knowledge is the main problem. Next to that, the panel believes that only a selected group of patients (with simple gastroschisis and good bowel conditions) is suitable for this procedure. |  |

Summary of judgements

|  | **Judgement** | | | | | | |
| --- | --- | --- | --- | --- | --- | --- | --- |
| **Problem** | No | Probably no | Probably yes | Yes |  | Varies | Don't know |
| **Desirable Effects** | **Trivial** | Small | Moderate | Large |  | Varies | Don't know |
| **Undesirable Effects** | Large | Moderate | Small | **Trivial** |  | Varies | Don't know |
| **Certainty of evidence** | **Very low** | Low | Moderate | High |  |  | No included studies |
| **Values** | Important uncertainty or variability | Possibly important uncertainty or variability | **Probably no important uncertainty or variability** | No important uncertainty or variability |  |  |  |
| **Balance of effects** | Favors the comparison | Probably favors the comparison | **Does not favor either the intervention or the comparison** | Probably favors the intervention | Favors the intervention | Varies | Don't know |
| **Resources required** | Large costs | Moderate costs | Negligible costs and savings | **Moderate savings** | Large savings | Varies | Don't know |
| **Certainty of evidence of required resources** | Very low | Low | Moderate | High |  |  | **No included studies** |
| **Cost effectiveness** | Favors the comparison | Probably favors the comparison | Does not favor either the intervention or the comparison | Probably favors the intervention | Favors the intervention | Varies | **No included studies** |
| **Equity** | Reduced | Probably reduced | **Probably no impact** | Probably increased | Increased | Varies | Don't know |
| **Acceptability** | No | Probably no | **Probably yes** | Yes |  | Varies | Don't know |
| **Feasibility** | No | Probably no | Probably yes | Yes |  | **Varies** | Don't know |

Type of recommendation

| Strong recommendation against the intervention | Conditional recommendation against the intervention | **Conditional recommendation for either the intervention or the comparison** | Conditional recommendation for the intervention | Strong recommendation for the intervention |
| --- | --- | --- | --- | --- |
| ○ | ○ | **●** | ○ | ○ |

Conclusions

| Recommendation |
| --- |
| The panel suggests to consider Bianchi’s approach as an option for treatment of neonates with simple gastroschisis and bowel in a good condition with no matting. |
|  |
| Justification |
| **Overall Justification**  Potentially, there may be some benefits to the patient with Bianchi’s approach if general anesthesia can be avoided. There was no evidence for harms in the current evaluation. Therefore, the panel agrees that if a surgeon is comfortable performing Bianchi’s approach and a patient has favorable bowel condition with no indication of possible complex disease, then it is worth considering as an option for closing the abdominal wall.  *Balance of effects*  The available limited evidence suggests that critical outcomes following Bianchi’s approach are comparable to those of primary closure under general anesthesia.  *Resources required* If general anesthesia can be avoided using Bianchi there will probably be savings on the surgical team. |

| Research priorities |
| --- |
| To evaluate the complete spectrum of effects for this intervention, future evaluation of Bianchi’s approach without general anesthesia versus primary closure with general anesthesia is warranted. The panel agrees that value could be added if future evaluation is prospective and randomized and includes pain or discomfort measurements, neurocognitive and cosmetic outcomes together with incidence of paraumbilical hernia in addition to the critical outcomes that were already evaluated. |

**References Summary**1. Zani A, Ruttenstock E, Davenport M, Ade-Ajayi N. Is there unity in Europe? First survey of EUPSA delegates on the management of gastroschisis. Eur J ediatr Surg. 2013 Feb;23(1):19-24.
2. Pet GE, Stark RA, Meehan JJ, Javid PJ. Outcomes of bedside sutureless umbilical closure without endotracheal intubation for gastroschisis repair in surgical infants. Am J Surg. 2017 May;213(5):958-962.
3. Rao SC, Pirie S, Minutillo C, et al. Ward reduction of gastroschisis in a single stage without general anaesthesia may increase the risk of short-term morbidities: results of a retrospective audit. Journal of Paediatrics and Child Health. 2009 Jun;45(6):384-388.
4. Choi WW, McBride CA, Bourke C, Borzi P, Choo K, Walker R, Nguyen T, Davies M, Donovan T, Cartwright D, Kimble RM. Long-term review of sutureless ward reduction in neonates with gastroschisis in the neonatal unit. J Pediatr Surg. 2012 Aug;47(8):1516-20.
5. Leadbeater K, Kumar R, Feltrin R. Ward reduction of gastroschisis: risk stratification helps optimise the outcome. Pediatr Surg Int. 2010 Oct;26(10):1001-5. doi: 10.1007/s00383-010-2659-5. PMID: 20658297.

| Question 2.2 |  |
| --- | --- |
|  | |
| **Should sutureless vs. Sutered approach be used for closure of the abdominal wall in gastroschisis?** | |
| **Population:** | Neonates with gastroschisis that have completed bowel and viscera reduction into the abdominal cavity by staged reduction with silo |
| **Intervention:** | Sutereless approach |
| **Comparison:** | Sutured approach |
| **Main outcomes:** | Mortality  Surgical site/wound infections (complications)  Duration of ventilation  Length of stay  PN-duration  Hernia formation |
| **Setting:** | Modern high resource hospital |

Assessment

| Problem Is the problem a priority? | | |
| --- | --- | --- |
| Judgement | Research evidence | Additional considerations |
| ○ No ○ Probably no ○ Probably yes ○ Yes ○ Varies ○ Don't know | Several factors may influence the choice of abdominal closure technique, such as general conditions, bowel appearance, abdominal capacity and compliance, the presence of complicated or simple gastroschisis. Primary reduction and closure or need for silo-staged reduction are related to different abdominal or general conditions. As a consequence, abdominal wall defect closure may be influenced by the type of reduction and results should be differentiated between newborns undergoing primary or silo-staged repair. For the purpose of this guideline the safety and efficacy of sutureless repair was considered in gastroschisis newborns undergoing silo-staged reduction. |  |
| Desirable Effects How substantial are the desirable anticipated effects? | | |
| Judgement | Research evidence | Additional considerations |
| ○ Trivial ● Small ○ Moderate ○ Large ○ Varies ○ Don't know | Sutureless closure seems to lower the risk of surgical site infection. The RR was calculated as 0.58 (95%CI 0.36-0.96), a significant risk difference favouring sutureless plastic closure.  Sutureless plastic closure seems to decrease the necessity of ventilation for gastroschisis patients who undergo silo-staged reduction. The effects on length of stay were unclear; there was no significant difference in one study but LoS was significantly decreased in the sample of Miyake et al., 2018. | There was no evidence for cosmetic result, but experts think that with plastic closure, there is probably a cosmetic benefit.  If plastic closure means that general anesthesia can be avoided, than this is a beneficial effect too. |
| Undesirable Effects How substantial are the undesirable anticipated effects? | | |
| Judgement | Research evidence | Additional considerations |
| ○ Large ○ Moderate ● Small ○  Trivial ○ Varies ○ Don't know | Youssef et al. (2016) found a significant increase in hernia formation after sutureless closure (41% vs. 19%, p=0.0001). | Very few of the hernia’s after gastroschisis closure needed surgical intervention (Choi et al. 2012, Sandler et al., 2004) |
| Certainty of evidence What is the overall certainty of the evidence of effects? | | |
| Judgement | Research evidence | Additional considerations |
| ● Very low ○ Low ○ Moderate ○ High ○ No included studies | The overall evidence level is rated with GRADE as very low. This is mainly due to observational (retrospective) nature of the studies included in the meta-analysis. Of the three studies included two were meta-analysis and one retrospective cohort study. We needed to take into account the overlap of some studies included in the two meta-analyses, which further reduced the available population when calculating the relative risk making the results at risk for imprecision. Authors of the meta-analysis by Miyaki et al.(2018) performed the analysis of their quality of evidence reporting a very low level and all included studies were rated to have a serious risk of bias (ROBINS-I). Overal bias risk for papers included in Youssef et al.(2016) was lower than for Miyaki et al., however, group comparability was suboptimal. |  |
| Values Is there important uncertainty about or variability in how much people value the main outcomes? | | |
| Judgement | Research evidence | Additional considerations |
| ○ Important uncertainty or variability ○ Possibly important uncertainty or variability ○Probably no important uncertainty or variability ● No important uncertainty or variability | There is agreement that prevention of a surgical site infection is a major advantage. Surgical site infection can cause huge problems in the vulnerable neonate, while if herniation occurs and if surgical intervention is indicated, you can operate on an older child, which bears less risks than a SSI in a neonate. |  |
| Balance of effects Does the balance between desirable and undesirable effects favor the intervention or the comparison? | | |
| Judgement | Research evidence | Additional considerations |
| ○ Favors the comparison ○ Probably favors the comparison ○ Does not favor either the intervention or the comparison ● Probably favors the intervention ○ Favors the intervention ○ Varies ○ Don't know | Looking at the critical outcomes, sutureless approach bears less risks for infection and this is a critical outcome. The avoidanace of general anesthesia, could be an additional benefit to sutureless closure. There are no direct undesirable effects of sutureless closure but in the long term it might increase the risk for herniation. |  |
| Resources required How large are the resource requirements (costs)? | | |
| Judgement | Research evidence | Additional considerations |
| ○ Large costs ○ Moderate costs ● Negligible costs and savings ○ Moderate savings ○ Large savings ○ Varies ○ Don't know | Orion et al. Evaluated the resource use of sutureless versus sutured approach and found no significant difference between the two. |  |
| Certainty of evidence of required resources What is the certainty of the evidence of resource requirements (costs)? | | |
| Judgement | Research evidence | Additional considerations |
| ● Very low ○ Low ○ Moderate ○ High ○ No included studies |  |  |
| Cost effectiveness Does the cost-effectiveness of the intervention favor the intervention or the comparison? | | |
| Judgement | Research evidence | Additional considerations |
| ○ Favors the comparison ○ Probably favors the comparison ○ Does not favor either the intervention or the comparison ○ Probably favors the intervention ○ Favors the intervention ○ Varies ● No included studies |  |  |
| Equity What would be the impact on health equity? | | |
| Judgement | Research evidence | Additional considerations |
| ○ Reduced ○ Probably reduced ● Probably no impact ○  Probably increased ○ Increased ○ Varies ○ Don't know |  |  |
| Acceptability Is the intervention acceptable to key stakeholders? | | |
| Judgement | Research evidence | Additional considerations |
| ○ No ○ Probably no ● Probably yes ○ Yes ○ Varies ○ Don't know |  |  |
| Feasibility Is the intervention feasible to implement? | | |
| Judgement | Research evidence | Additional considerations |
| ○ No ○ Probably no ● Probably yes ○ Yes ○ Varies ○ Don't know | It does require a training for surgeons, anesthetists and neonatologists |  |

Summary of judgements

|  | **Judgement** | | | | | | |
| --- | --- | --- | --- | --- | --- | --- | --- |
| **Problem** | No | Probably no | Probably yes | Yes |  | Varies | Don't know |
| **Desirable Effects** | Trivial | **Small** | Moderate | Large |  | Varies | Don't know |
| **Undesirable Effects** | Large | Moderate | **Small** | Trivial |  | Varies | Don't know |
| **Certainty of evidence** | **Very low** | Low | Moderate | High |  |  | No included studies |
| **Values** | Important uncertainty or variability | Possibly important uncertainty or variability | **Probably no important uncertainty or variability** | No important uncertainty or variability |  |  |  |
| **Balance of effects** | Favors the comparison | Probably favors the comparison | Does not favor either the intervention or the comparison | **Probably favors the intervention** | Favors the intervention | Varies | Don't know |
| **Resources required** | Large costs | Moderate costs | **Negligible costs and savings** | Moderate savings | Large savings | Varies | Don't know |
| **Certainty of evidence of required resources** | **Very low** | Low | Moderate | High |  |  | No included studies |
| **Cost effectiveness** | Favors the comparison | Probably favors the comparison | Does not favor either the intervention or the comparison | Probably favors the intervention | Favors the intervention | Varies | **No included studies** |
| **Equity** | Reduced | Probably reduced | **Probably no impact** | Probably increased | Increased | Varies | Don't know |
| **Acceptability** | No | Probably no | **Probably yes** | Yes |  | Varies | Don't know |
| **Feasibility** | No | Probably no | **Probably yes** | Yes |  | Varies | Don't know |

Type of recommendation

| Strong recommendation against the intervention | Conditional recommendation against the intervention | Conditional recommendation for either the intervention or the comparison | **Conditional recommendation for the intervention** | Strong recommendation for the intervention |
| --- | --- | --- | --- | --- |
| ○ | ○ | ○ | **●** | ○ |

Conclusions

| Recommendation |
| --- |
| The panel suggests sutureless closure in neonates with gastroschisis if this means that general anesthesia can be avoided.  If primary closure under general anesthesia is performed, the panel suggests a sutured closure to avoid possible hernia surgery later in life. |
|  |
| Justification |
| **Overall Justification**  There are cautious indications that the risk of SSI is lower if a sutureless approach is used. The avoidance of general anesthesia is an additional benefit.  *Quality of evidence*  The quality of evidence is very low, the panel believes it is possible that the actual effect is substantially different from the effect estimate for the critical outcome (SSI).  *Balance of effects*  The avoidance of general anesthesia is an additional benefit in favour of sutureless closure, however if the patient is already under general anesthesia in the operating room for primary closure of the gastroschisis, then this benefit is not an argument anymore. The evidence for decreasing the risk of SSI and the increase in the risk for herniation is both very low, so in these patients, there are little indications for benefit of one approach over the other. |

| Subgroup considerations |
| --- |
| These recommendations apply only to patients with simple gastroschisis. |

| Research priorities |
| --- |
| To obtain a higher quality of evidence, a well-designed, preferably randomized, prospective study is needed, focusing on both short and long term outcomes, in particular surgical site infection, the incidence of hernia and cosmetic results. |

**References Summary**

1) Miyake, H., Seo, S., O’Connell, J. S., Janssen Lok, M., & Pierro, A. (2019). Safety and usefulness of plastic closure in infants with gastroschisis: a systematic review and meta-analysis. Pediatric Surgery International, 35(1), 107-116.

2) Youssef, F., Gorgy, A., Arbash, G., Puligandla, P. S., & Baird, R. J. (2016). Flap versus fascial closure for gastroschisis: a systematic review and meta-analysis. Journal of pediatric surgery, 51(5), 718-725.

3) Fraser JD, Deans KJ, Fallat ME, Helmrath MA, Kabre R, Leys CM, Burns RC, Corkum K, Dillon PA, Downard CD, Gadepalli SK, Grabowski JE, Hernandez E, Hirschl RB, Johnson KN, Kohler JE, Landman MP, Landisch RM, Lawrence AE, Mak GZ, Minneci PC, Rymeski B, Sato TT, Scannell M, Slater BJ, Wilkinson KH, Wright TN, St Peter SD; Midwest Pediatric Surgery Consortium. Sutureless vs sutured abdominal wall closure for gastroschisis: Operative characteristics and early outcomes from the Midwest Pediatric Surgery Consortium. J Pediatr Surg. 2020 Nov;55(11):2284-2288. doi: 10.1016/j.jpedsurg.2020.02.017. Epub 2020 Feb 20. PMID: 32151403.

4) Choi, W. W., McBride, C. A., Bourke, C., Borzi, P., Choo, K., Walker, R., ... & Kimble, R. M. (2012). Long-term review of sutureless ward reduction in neonates with gastroschisis in the neonatal unit. *Journal of Pediatric Surgery*, 47(8), 1516-1520
5) Sandler, A., Lawrence, J., Meehan, J., Phearman, L., & Soper, R. (2004). A “plastic” sutureless abdominal wall closure in gastroschisis. *Journal of pediatric surgery*, 39(5), 738-741.

| Question 2.3 | |
| --- | --- |
| **Should Primary repair vs. primary reduction and delayed surgery be used for patients with complex gastroschisis ?** | |
| **Population:** | Patients with complex gastroschisis |
| **Intervention:** | Primary repair |
| **Comparison:** | Primary reduction and delayed surgery |
| **Main outcomes:** | Mortality, sepsis, major GI-complications, length of stay, time on TPN |
| **Setting:** | Modern high resource hospital |

Assessment

| Problem  Is the problem a priority? | | |
| --- | --- | --- |
| Judgement | Research evidence | Additional considerations |
| ○ No ○ Probably no ●Probably yes ○ Yes ○ Varies ○ Don't know | Complex gastroschisis (CGS) can be defined as gastroschisis with presence of bowel atresia, stenosis, perforation, volvulus or ischemia. These signs can be present at birth or develop later (for example due to abdominal compartment syndrome). Cases of gastroschisis categorized as complex have an increased morbidity and mortality rate in comparison with simple cases (Molik,2001).  Different surgical strategies for the closure of the abdominal wall are possible in newborns with CGS. The most prominent choice to make is the one between immediate intestinal resection (creating a stoma or performing anastomosis) or primary reduction and delayed surgery. Delay of non-urgent intestinal surgery, to allow for bowel recovery, has been promoted by multiple authors over the past three decades but has never been extensively evaluated. |  |
| Desirable Effects  How substantial are the desirable anticipated effects? | | |
| Judgement | Research evidence | Additional considerations |
| ○ Trivial ●Small ○ Moderate ○ Large ○ Varies ○ Don't know | One paper by Alsheri et al. 2013 compared early and late surgery. There were no significant differences between early surgery (<21 days) and late surgery group (>21) for outcomes mortality, line sepsis, wound infection and hospital stay.  In this sample, patients who had undergone early surgery, were less likely to be depending on TPN at 28 days of life (RR 0.41 , 95% CI , 0.17 to 1.02, p=0.06) but it did not reach significance. There was however significant difference in age at the first enteral feed (14.8 ± 2.6 days versus 44.7 ± 7.4 days, p= 0.002). This indicates that patients who undergo early surgery receive and tolerate enteral feeding earlier. |  |
| Undesirable Effects  How substantial are the undesirable anticipated effects? | | |
| Judgement | Research evidence | Additional considerations |
| ○ Large ○ Moderate ○ Small ○ Trivial ○ Varies ○ Don't know | No undesirable effects of early surgery on the critical and important outcomes were found. |  |
| Certainty of evidence  What is the overall certainty of the evidence of effects? | | |
| Judgement | Research evidence | Additional considerations |
| ○ Very low ○ Low ○ Moderate ○ High ○ No included studies | Few papers deal with this specific subject. No RCT but only systematic reviews of lower evidence or expert opinion could be analyzed. Low event numbers and small sample sizes may have caused imprecision and made it difficult to reach significance for some of the outcomes. A high risk of methodological bias is suspected because a multivariate analysis or clear correction for known confounding factors is missing. In this study, it is unknown why neonates had early or late surgery which induces the risk of selection bias. |  |
| Values  Is there important uncertainty about or variability in how much people value the main outcomes? | | |
| Judgement | Research evidence | Additional considerations |
| ○ Important uncertainty or variability ○ Possibly important uncertainty or variability ○ Probably no important uncertainty or variability ○ No important uncertainty or variability |  |  |
| Balance of effects  Does the balance between desirable and undesirable effects favor the intervention or the comparison? | | |
| Judgement | Research evidence | Additional considerations |
| ○ Favors the comparison ○ Probably favors the comparison ● Does not favor either the intervention or the comparison ○ Probably favors the intervention ○ Favors the intervention ○ Varies ○  Don't know | In some cases early intervention is probably favorable, establishing intestinal continuity, decreasing the risk of infection and lesser time on TPN are possibly large advantages. The quality of available data is very low which makes it difficult to judge based on the evidence, however, the favorable results in the early group, pose an argument for early repair in a well selected population.  The panel agrees that the therapeutic strategy should be well designed in these patients based on the general condition of the patient and the bowel. However these results showed, early establishment of intestinal continuity seems a safe option in selected patients with GS and atresia, where the procedure is seen as feasible. | The panel considered published opinions of other experts here. Bhat et al. (2020) suggest that the surgical approach of intestinal atresia must be individualized based on gestational age, birth weight, clinical status, and the condition of the bowel. In good condition, intestinal continuity restoration can be done together with primary closure.  Other experts (Sherif et al., 2018; Rentea, 2022) suggest a universal algorithm for these patients is not possible because every case is unique. |
| Resources required  How large are the resource requirements (costs)? | | |
| Judgement | Research evidence | Additional considerations |
| ○ Large costs ○ Moderate costs ○ Negligible costs and savings ● Moderate savings ○ Large savings ○ Varies ○ Don't know | Complex GS patients will always demand high costs both economical and personal. If early reconstruction may help to reduce hospital time, it could possibly reduce costs. |  |
| Certainty of evidence of required resources  What is the certainty of the evidence of resource requirements (costs)? | | |
| Judgement | Research evidence | Additional considerations |
| ○ Very low ○ Low ○ Moderate ○ High ● No included studies |  |  |
| Cost effectiveness  Does the cost-effectiveness of the intervention favor the intervention or the comparison? | | |
| Judgement | Research evidence | Additional considerations |
| ○ Favors the comparison ○ Probably favors the comparison ○ Does not favor either the intervention or the comparison ○ Probably favors the intervention ○ Favors the intervention ○ Varies ● No included studies |  |  |
| Equity  What would be the impact on health equity? | | |
| Judgement | Research evidence | Additional considerations |
| ○ Reduced ○ Probably reduced ○ Probably no impact ○ Probably increased ○ Increased ○ Varies ○ Don't know |  |  |
| Acceptability  Is the intervention acceptable to key stakeholders? | | |
| Judgement | Research evidence | Additional considerations |
| ○ No ○ Probably no ○ Probably yes ○ Yes ○ Varies ○ Don't know |  |  |
| Feasibility  Is the intervention feasible to implement? | | |
| Judgement | Research evidence | Additional considerations |
| ○ No ○ Probably no ○ Probably yes ○ Yes ○ Varies ○ Don't know |  |  |

Summary of judgements

|  | **Judgement** | | | | | | |
| --- | --- | --- | --- | --- | --- | --- | --- |
| **Problem** | No | Probably no | **Probably yes** | Yes |  | Varies | Don't know |
| **Desirable Effects** | Trivial | **Small** | Moderate | Large |  | Varies | Don't know |
| **Undesirable Effects** | Large | Moderate | Small | **Trivial** |  | Varies | Don't know |
| **Certainty of evidence** | **Very low** | Low | Moderate | High |  |  | No included studies |
| **Values** | Important uncertainty or variability | Possibly important uncertainty or variability | Probably no important uncertainty or variability | **No important uncertainty or variability** |  |  |  |
| **Balance of effects** | Favors the comparison | Probably favors the comparison | Does not favor either the intervention or the comparison | **Probably favors the intervention** | Favors the intervention | Varies | Don't know |
| **Resources required** | Large costs | Moderate costs | Negligible costs and savings | **Moderate savings** | Large savings | Varies | Don't know |
| **Certainty of evidence of required resources** | Very low | Low | Moderate | High |  |  | **No included studies** |
| **Cost effectiveness** | Favors the comparison | Probably favors the comparison | Does not favor either the intervention or the comparison | Probably favors the intervention | Favors the intervention | Varies | **No included studies** |
| **Equity** | Reduced | Probably reduced | **Probably no impact** | Probably increased | Increased | Varies | Don't know |
| **Acceptability** | No | Probably no | Probably yes | **Yes** |  | Varies | Don't know |
| **Feasibility** | No | Probably no | Probably yes | **Yes** |  | Varies | Don't know |

Type of recommendation

| Strong recommendation against the intervention | Conditional recommendation against the intervention | Conditional recommendation for either the intervention or the comparison | Conditional recommendation for the intervention | Strong recommendation for the intervention |
| --- | --- | --- | --- | --- |
| ○ | ○ | ○ | ○ | ○ |

Conclusions

| Recommendation |
| --- |
| The panel suggests primary intestinal repair for complex gastroschisis patients with atresia if the general condition and bowel allow for primary intestinal repair.  The panel suggests determining the treatment strategy for patients with complex gastroschisis based on the individual characteristics, general condition, and bowel condition of each patient. |
|  |
| Justification |
| The panel agrees that there is not enough evidence to recommend a universal therapeutic strategy cannot for all complex cases, and that the therapeutical strategy should be individually designed in these patients based on the general condition of the patient and the bowel. However, these results showed that if both are favorable, primary restoration of bowel continuity is a feasible option.  References Summary  Alshehri et al. J Pediatr Surg. 2013 Oct;48(10):2022-6.  Bhat, V., Moront, M., & Bhandari, V. (2020). Gastroschisis: a state-of-the-art review. Children, 7(12), 302.  Alani, M., & Rentea, R. M. (2022). Midgut malrotation. In StatPearls [Internet]. StatPearls Publishing.  Molik, K. A., Gingalewski, C. A., West, K. W., Rescorla, F. J., Scherer Iii, L. R., Engum, S. A., & Grosfeld, J. L. (2001). Gastroschisis: a plea for risk categorization. Journal of pediatric surgery, 36(1), 51-55. |

| Question 2.4 | |
| --- | --- |
| **Should Ventilation vs. no ventilation be used for patients with gastroschisis during silo reduction?** | |
| **Population:** | patients with gastroschisis during silo reduction |
| **Intervention:** | no ventilation |
| **Comparison:** | Ventilation |
| **Main outcomes:** | Mortality  Sedation  Infection: Sepsis or Bronchopneumonia  Days of Hospital Stay  Days to full enteral feeding  Bowel complications |
| **Setting:** | Modern high resource hospital |

Assessment

| Problem  Is the problem a priority? | | |
| --- | --- | --- |
| Judgement | Research evidence | Additional considerations |
| ○ No ○ Probably no ○ Probably yes ○ Yes ○ Varies ○ Don't know | Patients with gastroschisis undergoing gradual reduction with silo treatment have traditionally been intubated and mechanically ventilated for this purpose, placing the silo and performing final repair under general anesthesia. Since the emergence of preformed silos, as they can be placed without sutures and without general anesthesia, the option of keeping patients breathing spontaneously, without the need to be intubated and ventilated during the gradual reduction of intra-abdominal content, arises. Usually, this gradual reduction with silo takes several days, thus it would be beneficial for the patient to avoid endotracheal intubation and mechanical ventilation during this process. It would also associate the theoretical benefit of avoiding general anesthesia and its potential effects on neurodevelopment. This has not been properly evaluated for use in guidelines. |  |
| Desirable Effects  How substantial are the desirable anticipated effects? | | |
| Judgement | Research evidence | Additional considerations |
| ● Trivial ○ Small ○ Moderate ○ Large ○ Varies ○ Don't know | One study (Hong et al.) compared ventilated and non-ventilated children undergoing a silo procedure. Conversely, general anesthesia was not avoided because final closure was done operatively. In this small sample, no particular desirable effects were observed in the non-ventilation group, but mortality and infection rates were low. In the other study (Owen et al.), preformed silos with no general anesthesia (PSnoGA) was compared to primary operative closure. In the PSnoGA group 3/21 suffered wound infections and 2/21 had respiratory infections. This was not significantly different from the group that received immediate facial closure. | Avoidance of any general anesthesia and intubation can be beneficial to patients. |
| Undesirable Effects  How substantial are the undesirable anticipated effects? | | |
| Judgement | Research evidence | Additional considerations |
| ○ Large ○ Moderate ○ Small ● Trivial ○ Varies  ○ Don't know | No undesirable effects could be observed. | None of the studies reported patient’s baseline parameters (e.g. respiratory rate, heart rate, etc.) during reduction maneuvers, therefore patients wellbeing during these procedures can be difficultly objectivized. |
| Certainty of evidence  What is the overall certainty of the evidence of effects? | | |
| Judgement | Research evidence | Additional considerations |
| ● Very low ○ Low ○ Moderate ○ High ○  No included studies | The quality of the evidence was very low. The two observational studies together only provided 26 non-ventilated patients which puts the results at risk for imprecision and therefore very low quality evidence. Both included studies are at high risk of bias due to confounding and are dated (inclusion between 1990 and 2008). |  |
| Values  Is there important uncertainty about or variability in how much people value the main outcomes? | | |
| Judgement | Research evidence | Additional considerations |
| ○ Important uncertainty or variability ○ Possibly important uncertainty or variability ○ Probably no important uncertainty or variability ○ No important uncertainty or variability | The evaluated outcomes are important to all, but patient wellbeing and comfort are not evaluated and not described in available studies. This is potentially a very critical outcome for specialists but also for parents. |  |
| Balance of effects  Does the balance between desirable and undesirable effects favor the intervention or the comparison? | | |
| Judgement | Research evidence | Additional considerations |
| ○ Favors the comparison ○ Probably favors the comparison ○ Does not favor either the intervention or the comparison ○ Probably favors the intervention ○ Favors the intervention ○ Varies ○ Don't know | Weak evidence that it results in similar outcomes on infection, enteral feeding and bowel complications. Due to the small patient numbers the evidence is not robust enough to base any decisions on. The panel members feel that the fact that no associations with negative effects were observed in these two studies, indicates that the procedure is feasible but that we can not recommend it if there is no evidence for the benefits of it. At this point, the only (theoretical) benefit could be the avoidance of GA. | No objective measures of pain or discomfort were included in these studies. |
| Resources required  How large are the resource requirements (costs)? | | |
| Judgement | Research evidence | Additional considerations |
| ○ Large costs ○ Moderate costs ○ Negligible costs and savings ○ Moderate savings ○ Large savings ○ Varies ● Don't know | If the intervention is effective, it should produce savings for the newborn and the hospital that take care of the patient. |  |
| Certainty of evidence of required resources  What is the certainty of the evidence of resource requirements (costs)? | | |
| Judgement | Research evidence | Additional considerations |
| ○ Very low ○ Low ○ Moderate ○ High ● No included studies |  |  |
| Cost effectiveness  Does the cost-effectiveness of the intervention favor the intervention or the comparison? | | |
| Judgement | Research evidence | Additional considerations |
| ○ Favors the comparison ○ Probably favors the comparison ○ Does not favor either the intervention or the comparison ○ Probably favors the intervention ○ Favors the intervention ○ Varies ● No included studies |  |  |
| Equity  What would be the impact on health equity? | | |
| Judgement | Research evidence | Additional considerations |
| ○ Reduced ○ Probably reduced ○ Probably no impact ○ Probably increased ○ Increased ○ Varies ● Don't know |  |  |
| Acceptability  Is the intervention acceptable to key stakeholders? | | |
| Judgement | Research evidence | Additional considerations |
| ○ No ○ Probably no ○ Probably yes ○ Yes ○ Varies ● Don't know | Comfort and pain in the patient are not evaluated and this should be done to reach acceptability |  |
| Feasibility  Is the intervention feasible to implement? | | |
| Judgement | Research evidence | Additional considerations |
| ○ No ○ Probably no ● Probably yes ○ Yes ○ Varies ○ Don't know |  |  |

Summary of judgements

|  | **Judgement** | | | | | | |
| --- | --- | --- | --- | --- | --- | --- | --- |
| **Problem** | No | Probably no | **Probably yes** | Yes |  | Varies | Don't know |
| **Desirable Effects** | **Trivial** | Small | Moderate | Large |  | Varies | **Don't know** |
| **Undesirable Effects** | Large | Moderate | Small | **Trivial** |  | Varies | **Don't know** |
| **Certainty of evidence** | **Very low** | Low | Moderate | High |  |  | No included studies |
| **Values** | Important uncertainty or variability | **Possibly important uncertainty or variability** | Probably no important uncertainty or variability | No important uncertainty or variability |  |  |  |
| **Balance of effects** | Favors the comparison | Probably favors the comparison | Does not favor either the intervention or the comparison | Probably favors the intervention | Favors the intervention | Varies | **Don't know** |
| **Resources required** | Large costs | Moderate costs | Negligible costs and savings | Moderate savings | Large savings | Varies | **Don't know** |
| **Certainty of evidence of required resources** | Very low | Low | Moderate | High |  |  | **No included studies** |
| **Cost effectiveness** | Favors the comparison | Probably favors the comparison | Does not favor either the intervention or the comparison | Probably favors the intervention | Favors the intervention | Varies | **No included studies** |
| **Equity** | Reduced | Probably reduced | Probably no impact | Probably increased | Increased | Varies | **Don't know** |
| **Acceptability** | No | Probably no | Probably yes | Yes |  | Varies | **Don't know** |
| **Feasibility** | No | Probably no | **Probably yes** | Yes |  | Varies | Don't know |

Type of recommendation

| Strong recommendation against the intervention | Conditional recommendation against the intervention | **Conditional recommendation for either the intervention or the comparison** | Conditional recommendation for the intervention | Strong recommendation for the intervention |
| --- | --- | --- | --- | --- |
| ○ | ○ | **●** | ○ | ○ |

Conclusions

| Recommendation |
| --- |
| The panel suggests to consider spontaneous breathing as an alternative to intubation and mechanical ventilation in patients with stable conditions and simple gastroschisis if staged closure is the treatment option of choice.  The panel recommends close monitoring of comfort and pain in patients undergoing staged closure while breathing spontaneously using objective measurements. |
| \| Justification \| \| --- \| \| **Overall Justification**  Available evidence about silo treatment without ventilation and general anesthesia make it a feasible procedure for patients with simple gastroschisis, stable condition, and no need for total bowel evaluation. An adequate measurement and management of pain throughout the process is mandatory in these patients.  *Balance of effects*  An adequate measurement and management of pain throughout the process is mandatory in these patients. \| |
|  |

| Subgroup considerations | |
| --- | --- |
| The spontaneous breathing approach is only indicated for patients with simple gastroschisis and stable conditions. | |
| Research priorities | |
| There is a need for randomized controlled trials to compare silo-staged reduction with and without intubation and ventilation of the patients. These trials must focus on non-complicated gastroschisis and should include pain and discomfort assessment and cost analysis, as well as general morbidity outcomes. Alongside, longitudinal studies comparing neurodevelopment in patients who underwent silo treatment with and without general anesthesia/ventilation might help define the risk-benefit rates of the two approaches. | |
| Question 2.5 | |
| **Should biological vs. non-absorbable patches be used for patients with gastroschisis?** | |
| **Population:** | Patients with gastroschisis, with abdominal wall defect that is unfit for closure after silo reduction |
| **Intervention:** | Biological patches |
| **Comparison:** | Non-absorbable patches (synthetic) |
| **Main outcomes:** | - Mortality  - Infection (skin infection around patch or sepsis)  - Ventral herniation |
| **Setting:** | Modern high resource hospital |

Assessment

| Problem  Is the problem a priority? | | |
| --- | --- | --- |
| Judgement | Research evidence | Additional considerations |
| ○ No ○ Probably no ○ Probably yes ○ Yes ○ Varies ○ Don't know | Caretakers around world use different options according to local preferences and experience. However, it is unclear if there are important outcome differences between the options or if this situation has led to unwanted variance in patient care. |  |
| Desirable Effects  How substantial are the desirable anticipated effects? | | |
| Judgement | Research evidence | Additional considerations |
| ○ Trivial ○ Small ○ Moderate ○ Large ○ Varies ● Don't know | No evidence comparing the two options for the desired outcomes was found. | One long term follow-up study on GoreTex mesh indicated that the mesh was helpful in reducing the defect and that the mesh could be removed the the abdominal wall fully closed in 22/26 patients with GS after a median of 9.5 days. |
| Undesirable Effects/  How substantial are the undesirable anticipated effects? | | |
| Judgement | Research evidence | Additional considerations |
| ○ Large ○ Moderate ○ Small ○ Trivial ○ Varies ● Don't know | No evidence comparing the two options for the desired outcomes was found. | From anecdotal evidence, infection rates in synthetic mesh (Gore-Tex) are going up to 30%. Skin infections are most frequently reported. In the largest series by Risby et al. (2016) 10% were operated for a ventral hernia at a follow-up varying from 1-18 years. It should be taken into consideration that with a synthetic patch, children will always need re-operation to remove the mesh.  For biologic mesh, single arm series indicate that 10% of patients had hernia surgery (Chivukula et al. 2017). Ventral hernia is possible to repair, so not most important outcome. |
| Certainty of evidence  What is the overall certainty of the evidence of effects? | | |
| Judgement | Research evidence | Additional considerations |
| ○ Very low ○ Low ○ Moderate ○ High ● No included studies |  |  |
| Values  Is there important uncertainty about or variability in how much people value the main outcomes? | | |
| Judgement | Research evidence | Additional considerations |
| ○ Important uncertainty or variability ○ Possibly important uncertainty or variability ○ Probably no important uncertainty or variability ○ No important uncertainty or variability | Mortality is the most important outcome, however mortality is often not (reported to be) related to the mesh procedures and gives therefore limited insight in the effects of the procedure. | The cosmetic result is possibly an important outcome to patients. There are no long-term follow-ups on patients satisfaction with this. Surgeons in the guideline panel hardly see any patients that ask for a scar correction so believe that patients are not bothered by the scars. However, it was not possible to test this hypothesis in the goal population  An important outcome measure according to the panel is an abdominal wall specific QoL long term measurement. This can help the evaluation of mesh results as some late onset mesh complications (bowel adhesions, ileus) have possibly large consequences for QoL.  In these children the Length of Stay is a proxy for complications and health, therefore this is an important outcome that is missing from current data. The same holds for time to closure as the primary aim is to close the abdomen a delay in closure corresponds to the success rate of the closing. |
| Balance of effects  Does the balance between desirable and undesirable effects favor the intervention or the comparison? | | |
| Judgement | Research evidence | Additional considerations |
| ○ Favors the comparison ○ Probably favors the comparison ○ Does not favor either the intervention or the comparison ○ Probably favors the intervention ○ Favors the intervention ○ Varies ○ Don't know |  | Volume combined with surgeon’s expertise is associated to better outcomes (Chowdhury et al.2007). Data on location of the patch and mesh exposure (bridging vs. augmentation) missing, additional outcome measure should be long-term "abdominal wall" QoL |
| Resources required  How large are the resource requirements (costs)? | | |
| Judgement | Research evidence | Additional considerations |
| ○ Large costs ○ Moderate costs ○ Negligible costs and savings ○ Moderate savings ○ Large savings ○ Varies ● Don't know |  |  |
| Certainty of evidence of required resources  What is the certainty of the evidence of resource requirements (costs)? | | |
| Judgement | Research evidence | Additional considerations |
| ○ Very low ○ Low ○ Moderate ○ High ● No included studies |  |  |
| Cost effectiveness  Does the cost-effectiveness of the intervention favor the intervention or the comparison? | | |
| Judgement | Research evidence | Additional considerations |
| ○ Favors the comparison ○ Probably favors the comparison ○ Does not favor either the intervention or the comparison ○ Probably favors the intervention ○ Favors the intervention ○ Varies ● No included studies |  |  |
| Equity  What would be the impact on health equity? | | |
| Judgement | Research evidence | Additional considerations |
| ○ Reduced ○ Probably reduced ○ Probably no impact ○ Probably increased ○ Increased ○ Varies ● Don't know |  |  |
| Acceptability  Is the intervention acceptable to key stakeholders? | | |
| Judgement | Research evidence | Additional considerations |
| ○ No ○ Probably no ○ Probably yes ○ Yes ○ Varies ● Don't know |  |  |
| Feasibility  Is the intervention feasible to implement? | | |
| Judgement | Research evidence | Additional considerations |
| ○ No ○ Probably no ○ Probably yes ○ Yes ○ Varies ● Don't know |  |  |

Summary of judgements

|  | **Judgement** | | | | | | |
| --- | --- | --- | --- | --- | --- | --- | --- |
| **Problem** | No | Probably no | Probably yes | Yes |  | Varies | Don't know |
| **Desirable Effects** | Trivial | Small | Moderate | Large |  | Varies | **Don't know** |
| **Undesirable Effects** | Large | Moderate | Small | Trivial |  | Varies | **Don't know** |
| **Certainty of evidence** | Very low | Low | Moderate | High |  |  | **No included studies** |
| **Values** | Important uncertainty or variability | Possibly important uncertainty or variability | Probably no important uncertainty or variability | No important uncertainty or variability |  |  |  |
| **Balance of effects** | Favors the comparison | Probably favors the comparison | Does not favor either the intervention or the comparison | Probably favors the intervention | Favors the intervention | Varies | Don't know |
| **Resources required** | Large costs | Moderate costs | Negligible costs and savings | Moderate savings | Large savings | Varies | **Don't know** |
| **Certainty of evidence of required resources** | Very low | Low | Moderate | High |  |  | **No included studies** |
| **Cost effectiveness** | Favors the comparison | Probably favors the comparison | Does not favor either the intervention or the comparison | Probably favors the intervention | Favors the intervention | Varies | **No included studies** |
| **Equity** | Reduced | Probably reduced | Probably no impact | Probably increased | Increased | Varies | Don't know |
| **Acceptability** | No | Probably no | Probably yes | Yes |  | Varies | **Don't know** |
| **Feasibility** | No | Probably no | Probably yes | Yes |  | Varies | **Don't know** |

Type of recommendation

| Strong recommendation against the intervention | Conditional recommendation against the intervention | **Conditional recommendation for either the intervention or the comparison** | Conditional recommendation for the intervention | Strong recommendation for the intervention |
| --- | --- | --- | --- | --- |
| ○ | ○ | **●** | ○ | ○ |

Conclusions

| Recommendation |
| --- |
| The panel suggests to use either synthetic or biologic mesh in cases where fascial closure is not feasible after silo reduction. The final decision for the type of mesh should be based on inhouse expertise of the pediatric surgical team. |
|  |
| Justification |
| *Balance of effects*  There are no comparative studies to indicate the use of one type of mesh above others. Older studies have indicated that the combination of treatment in high volume centers and treatment according to surgeon’s expertise is associated with better outcomes in pediatric surgery. |

| Research priorities |
| --- |
| The goal population for this question is a very rare subgroup of gastroschisis patients. Therefore, European collaboration to retrieve and analyze data is of high importance for further development of evidence-based practice. The panel suggests a European mesh registry, not only for gastroschisis but also for other defects for which mesh is applied. The registry ideally includes a wide variety of outcomes including long term disease specific quality of life and esthetic results. |

References Summary

Risby K, Jakobsen MS, Qvist N. Congenital abdominal wall defects. Stagged closure by Dual Mesh. J Neonatal Surg. 2016;5:2.

Chivukula KK, Holands C. Human acellular dermal matrix for neonates with complex abdominal wall defects. Short-term and long-term outcomes. Am Surg 2012;78:E346-8.

Chowdhury, M. M., Dagash, H., & Pierro, A. (2007). A systematic review of the impact of volume of surgery and specialization on patient outcome. Journal of British Surgery, 94(2), 145-161.

### Module 3

| Question 3.1a | |
| --- | --- |
| **Should enteral feeds <7 days of life vs. >7 days of life be used for patients with gastroschisis?** | |
| **Population:** | Patients with gastroschisis |
| **Intervention:** | enteral feeds <7 days of life |
| **Comparison:** | >7 days of life |
| **Main outcomes:** | -Time to full enteral feeding - Time on parenteral nutrition - Length of hospital stay - Infection or sepsis |
| **Setting:** | Modern high resource hospital |

Assessment

| Problem  Is the problem a priority? | | |
| --- | --- | --- |
| Judgement | Research evidence | Additional considerations |
| ○ No ○ Probably no **○ Probably yes** ○ Yes ○ Varies ○ Don't know | For patients with gastroschisis, the time to full enteral feeding after surgical repair is longer than other neonatal abdominal surgical conditions. Longer time to achieve full enteral feeding implies more time on parenteral nutrition and dependence on a central line. These factors clearly have an impact on the risk of infection, adverse effects of parenteral nutrition and on length of hospital stay and overall cost. It seems reasonable that any action focused on reducing the time to full feeds will have a positive effect on dependence on parenteral nutrition, infection, cost or hospital stay). To date it is unclear which is the right time to start enteral feeds in order to achieve these hypothesized benefits. |  |
| Desirable Effects  How substantial are the desirable anticipated effects? | | |
| Judgement | Research evidence | Additional considerations |
| ○ Trivial ● Small ○ Moderate ○ Large ○ Varies ○ Don't know | Aljahdali et al. (2013) analyzed the effect of timing of first feeds (TTFF) on outcome. Differences between four groups were analyzed. TTFF was ≤7 days (n=70) for group 1, 8-14 days (n= 253) for group 2, 15-21 days (n=152) for group 3 and >21 days (n = 95) for group 4.  Groups 3 and 4 both had significantly higher rates of surgical site infection (SSI) compared to group 1 (OR 2.4 respectively 4.8). | In most children, clinical signs can indicate if feeds can be started/increased or not. If there is a lot of bilious vomit, then it is probably not the right time to start. However, if a baby has only small aspiration volumes, it is reasonable to try and initiate or increase feeds. |
| Undesirable Effects  How substantial are the undesirable anticipated effects? | | |
| Judgement | Research evidence | Additional considerations |
| ○ Large ○Moderate  ●Small ○ Trivial ○ Varies ○ Don't know | Length of stay and time on TPN were significantly longer for group 1 (<7 days) compared to group 2(8-14 days). Because of possible complications that can arise with PN dependency, longer time on TPN is perceived as an important undesirable effect in this question. However, because the confidence intervals of group 1 and 2 are, despite the significant difference, overlapping, there are also patients with a shorter time of TPN and length of stay in group 1 over group 2. The panel believes therefore the effect is small. | All outcomes were worse in groups 3 and 4 (start of feeding later than day 15) and regression analysis indicated that each day delay in feeding is associated with 1.5 days longer hospital stay. |
| Certainty of evidence  What is the overall certainty of the evidence of effects? | | |
| Judgement | Research evidence | Additional considerations |
| ● Very low ○ Low ○ Moderate ○ High ○ No included studies | There is no RCT that specifically addressed our question of interest. All the other studies that have been considered were case series. They showed a high risk of bias because they included no randomized control group and used historical series to compare. Additionally, the intervention included not only the moment at which enteral feedings started but also the amount and speed of feed increments or antibiotic or central line policy that are important confounders. For these reasons, we considered that the quality of evidence in our research very low. | All studies were retrospective. Therefore, we cannot exclude the possibility that the occurrence of an infection was the reason not to start feeds within the first week. |
| Values  Is there important uncertainty about or variability in how much people value the main outcomes? | | |
| Judgement | Research evidence | Additional considerations |
| ○ Important uncertainty or variability ○ Possibly important uncertainty or variability ○ Probably no important uncertainty or variability ○ No important uncertainty or variability |  |  |
| Balance of effects  Does the balance between desirable and undesirable effects favor the intervention or the comparison? | | |
| Judgement | Research evidence | Additional considerations |
| ○ Favors the comparison ○ Probably favors the comparison ○ Does not favor either the intervention or the comparison ○ Probably favors the intervention ○ Favors the intervention ○ Varies ● Don't know | The evidence is too weak to make a judgement, especially because the effects on the main outcomes (infection rates and time on PN) are in favor of opposite directions. |  |
| Resources required  How large are the resource requirements (costs)? | | |
| Judgement | Research evidence | Additional considerations |
| ○ Large costs ○ Moderate costs ○ Negligible costs and savings ○ Moderate savings ○ Large savings ○ Varies ● Don't know | The cost of starting feeds one day or another is negligible. The amount of savings that you (as a hospital) or the patient can achieve from decreasing the time on PN and the hospital stay, may be moderate. |  |
| Certainty of evidence of required resources  What is the certainty of the evidence of resource requirements (costs)? | | |
| Judgement | Research evidence | Additional considerations |
| ○ Very low ○ Low ○ Moderate ○ High ● No included studies |  |  |
| Cost effectiveness  Does the cost-effectiveness of the intervention favor the intervention or the comparison? | | |
| Judgement | Research evidence | Additional considerations |
| ○ Favors the comparison ○ Probably favors the comparison ○ Does not favor either the intervention or the comparison ○ Probably favors the intervention ○ Favors the intervention ○ Varies ● No included studies |  |  |
| Equity  What would be the impact on health equity? | | |
| Judgement | Research evidence | Additional considerations |
| ○ Reduced ○ Probably reduced ○ Probably no impact ○ Probably increased ○ Increased ○ Varies ● Don't know |  |  |
| Acceptability  Is the intervention acceptable to key stakeholders? | | |
| Judgement | Research evidence | Additional considerations |
| ○ No ○ Probably no ○ Probably yes ○ Yes ○ Varies ○ Don't know |  |  |
| Feasibility  Is the intervention feasible to implement? | | |
| Judgement | Research evidence | Additional considerations |
| ○ No ○ Probably no ○ Probably yes ○ Yes ○ Varies ○ Don't know |  |  |

Summary of judgements

|  | **Judgement** | | | | | | |
| --- | --- | --- | --- | --- | --- | --- | --- |
| **Problem** | No | Probably no | Probably yes | Yes |  | Varies | Don't know |
| **Desirable Effects** | Trivial | **Small** | Moderate | Large |  | Varies | Don't know |
| **Undesirable Effects** | Large | Moderate | **Small** | Trivial |  | Varies | Don't know |
| **Certainty of evidence** | **Very low** | Low | Moderate | High |  |  | No included studies |
| **Values** | Important uncertainty or variability | Possibly important uncertainty or variability | **Probably no important uncertainty or variability** | No important uncertainty or variability |  |  |  |
| **Balance of effects** | Favors the comparison | Probably favors the comparison | Does not favor either the intervention or the comparison | Probably favors the intervention | Favors the intervention | Varies | **Don't know** |
| **Resources required** | Large costs | Moderate costs | Negligible costs and savings | Moderate savings | Large savings | Varies | **Don't know** |
| **Certainty of evidence of required resources** | Very low | Low | Moderate | High |  |  | **No included studies** |
| **Cost effectiveness** | Favors the comparison | Probably favors the comparison | Does not favor either the intervention or the comparison | Probably favors the intervention | Favors the intervention | Varies | **No included studies** |
| **Equity** | Reduced | Probably reduced | Probably no impact | Probably increased | Increased | Varies | **Don't know** |
| **Acceptability** | No | Probably no | Probably yes | **Yes** |  | Varies | Don't know |
| **Feasibility** | No | Probably no | Probably yes | **Yes** |  | Varies | Don't know |

Type of recommendation

| Strong recommendation against the intervention | Conditional recommendation against the intervention | **Conditional recommendation for either the intervention or the comparison** | Conditional recommendation for the intervention | Strong recommendation for the intervention |
| --- | --- | --- | --- | --- |
| ○ | ○ | **●** | ○ | ○ |

Conclusions

| Recommendation |
| --- |
| The panel suggests to start enteral feeding within the first 14 days post repair  If the neonate's condition is favorable, then starting enteral feeds before the 7^th^ day can be considered. |
|  |
| Justification |
| *Balance of effects* The evidence is too weak to make a judgement, especially because the main outcome effects (infection rates and time on PN) are in favor of opposite directions.  *Desirable effects – additional considerations* In most children, clinical signs will indicate if feedings can be started/increased or not. Bilous vomiting and/or large aspiration volumes are a contraindication to start/increase enteral feeds. However, if a baby has only small aspiration volumes, it is reasonable to try and start or increase the feeds.  *Undesirable effects – additional considerations*  All outcomes were worse in groups 3 and 4 (start of feeding later than day 15) and regression analysis indicated that each day delay in feeding, correlates to 1.5 days longer hospital admission. |

| Research priorities |
| --- |
|  |

References Summary
Aljahdali, A., Mohajerani, N., & Skarsgard, E. D. (2013). Effect of timing of enteral feeding on outcome in gastroschisis. Journal of Pediatric Surgery, 48(5), 971-976*.*

| Question 3.1b | |
| --- | --- |
| **Should protocolized vs. not protocolized feeding strategies be used for patients with gastroschisis ?** | |
| **Population:** | Patients with gastroschisis in whom the abdomen is closed |
| **Intervention:** | Protocolized feeding strategies for enteral feeding |
| **Comparison:** | Non-protocolized feeding strategies for enteral feeding |
| **Main outcomes:** | - Time to full enteral feeding  - Time on parenteral nutrition  - Length of hospital stay  - Infection or sepsis |
| **Setting:** | Modern high resource hospital |

Assessment

| Problem  Is the problem a priority? | | |
| --- | --- | --- |
| Judgement | Research evidence | Additional considerations |
| ○ No ○ Probably no ○ Probably yes ○ Yes ○ Varies ○ Don't know |  |  |
| Desirable Effects  How substantial are the desirable anticipated effects? | | |
| Judgement | Research evidence | Additional considerations |
| ○ Trivial ● Small ○ Moderate ○ Large ○ Varies ○ Don't know | The relative risk of sepsis in the traditional group compared to the intervention protocol group was RR 3.5. For other outcomes, such as time to full enteral feeds and length of stay no significant differences were found. | In a survey that included 23 parents of gastroschisis patients from 5 European countries, 60% reported that their hospital used a specific feeding protocol and all of them found the protocol helpful (Gastroschisis guideline panel, 2022). |
| Undesirable Effects  How substantial are the undesirable anticipated effects? | | |
| Judgement | Research evidence | Additional considerations |
| ○ Large ○ Moderate ○ Small ● Trivial ○ Varies ○ Don't know | No undesirable effects were reported. |  |
| Certainty of evidence  What is the overall certainty of the evidence of effects? | | |
| Judgement | Research evidence | Additional considerations |
| ● Very low ○ Low ○ Moderate ○ High ○ No included studies | There are no RCT that specifically addressed our question of interest. All the other studies that have been considered were case series. They showed a high risk of bias because they included no randomized control group and used historical series to compare. In the meta-analysis of Raduma et al. studies comparing a cohort of gastroschisis patients without a feeding protocol (traditional approach) to a cohort with a feeding protocol were analyzed. The feeding protocols were different among studies and included different combinations of interventions (starting day, volume of feeds, amounts of increase, type of feeding etc.). Therefore, the evidence only gives indications of the benefits of using a feeding protocol and cannot tell us which protocol to follow. |  |
| Values  Is there important uncertainty about or variability in how much people value the main outcomes? | | |
| Judgement | Research evidence | Additional considerations |
| ○ Important uncertainty or variability ○ Possibly important uncertainty or variability ● Probably no important uncertainty or variability ○ No important uncertainty or variability |  |  |
| Balance of effects  Does the balance between desirable and undesirable effects favor the intervention or the comparison? | | |
| Judgement | Research evidence | Additional considerations |
| ○ Favors the comparison ○ Probably favors the comparison ○ Does not favor either the intervention or the comparison ○ Probably favors the intervention ○ Favors the intervention ○ Varies ○ Don't know |  |  |
| Resources required  How large are the resource requirements (costs)? | | |
| Judgement | Research evidence | Additional considerations |
| ○ Large costs ○ Moderate costs ○ Negligible costs and savings ● Moderate savings ○ Large savings ○ Varies ○ Don't know | One study has performed a cost analysis. After correction for gestational age and length of NICU stay in a multivariate regression analysis, Utria et al.(2021) concluded a reduction in hospital costs associated with the use of a feeding protocol of 9.77% | The panel recognizes the mechanism of higher standardization leading to better care and lower costs. |
| Certainty of evidence of required resources  What is the certainty of the evidence of resource requirements (costs)? | | |
| Judgement | Research evidence | Additional considerations |
| ○ Very low ● Low ○ Moderate ○ High ○ No included studies | In the study of Utria et al. (2021) Multivariate analysis was performed to correct for the most important confounding factors of hospital costs. However, it remains evidence from a single observational study that used a historical cohort to compare. |  |
| Cost effectiveness  Does the cost-effectiveness of the intervention favor the intervention or the comparison? | | |
| Judgement | Research evidence | Additional considerations |
| ○ Favors the comparison ○ Probably favors the comparison ○ Does not favor either the intervention or the comparison ● Probably favors the intervention ○ Favors the intervention ○ Varies ○  No included studies | The only existing evidence indicates lower costs in a situation with a standardized feeding protocol. The panel recognizes the mechanism of higher standardization leading to better care and lower costs and therefore feels that it is reasonable that cost-effectiveness will be in favor of protocolized feeding. |  |
| Equity  What would be the impact on health equity? | | |
| Judgement | Research evidence | Additional considerations |
| ○ Reduced ○ Probably reduced ○ Probably no impact ○ Probably increased ○ Increased ○ Varies ○ Don't know | Protocolized feeding is at the moment not a standard practice in all European expert centers. If all children are fed through a protocol, they can all have equal opportunities for the benefits of it. |  |
| Acceptability  Is the intervention acceptable to key stakeholders? | | |
| Judgement | Research evidence | Additional considerations |
| ○ No ○ Probably no ● Probably yes ○ Yes ○ Varies ○ Don't know |  |  |
| Feasibility  Is the intervention feasible to implement? | | |
| Judgement | Research evidence | Additional considerations |
| ○ No ○ Probably no ● Probably yes ○ Yes ○ Varies ○ Don't know |  |  |

Summary of judgements

|  | **Judgement** | | | | | | |
| --- | --- | --- | --- | --- | --- | --- | --- |
| **Problem** | No | Probably no | Probably yes | Yes |  | Varies | Don't know |
| **Desirable Effects** | Trivial | **Small** | Moderate | Large |  | Varies | Don't know |
| **Undesirable Effects** | Large | Moderate | Small | **Trivial** |  | Varies | Don't know |
| **Certainty of evidence** | **Very low** | Low | Moderate | High |  |  | No included studies |
| **Values** | Important uncertainty or variability | Possibly important uncertainty or variability | **Probably no important uncertainty or variability** | No important uncertainty or variability |  |  |  |
| **Balance of effects** | Favors the comparison | Probably favors the comparison | Does not favor either the intervention or the comparison | **Probably favors the intervention** | Favors the intervention | Varies | Don't know |
| **Resources required** | Large costs | Moderate costs | Negligible costs and savings | **Moderate savings** | Large savings | Varies | Don't know |
| **Certainty of evidence of required resources** | **Very low** | Low | Moderate | High |  |  | No included studies |
| **Cost effectiveness** | Favors the comparison | Probably favors the comparison | Does not favor either the intervention or the comparison | **Probably favors the intervention** | Favors the intervention | Varies | No included studies |
| **Equity** | Reduced | Probably reduced | Probably no impact | **Probably increased** | Increased | Varies | Don't know |
| **Acceptability** | No | Probably no | **Probably yes** | Yes |  | Varies | Don't know |
| **Feasibility** | No | Probably no | **Probably yes** | Yes |  | Varies | Don't know |

Type of recommendation

| Strong recommendation against the intervention | Conditional recommendation against the intervention | Conditional recommendation for either the intervention or the comparison | **Conditional recommendation for the intervention** | Strong recommendation for the intervention |
| --- | --- | --- | --- | --- |
| ○ | ○ | ○ | **●** | ○ |

Conclusions

| Recommendation |
| --- |
| The panel suggests the implementation of a feeding protocol in centers to start enteral feeding after closure of the abdomen in neonates with gastroschisis. |
|  |
| Justification |
| *Desirable effects* There are indications that protocolized feeding is associated with lower risks for septicemia.  *Additional considerations desirable effects* Sixty percent of the parents of children with gastroschisis from different European countries who replied to the survey reported that standardized feeding protocols were used and were helpful  *Cost-effectiveness* A recent (2021) multivariate analysis indicates that a protocolized feeding strategy is associated with a 9.77% reduce in total hospital costs per patient. |

| Research priorities |
| --- |
| The analyzed data in chapter 8a and 8b provide some indications on how to handle the start of enteral feeding after closing the abdomen. There is not enough evidence to conclude how the exact protocol to achieve the estimated benefits should look like. The guideline panel firmly believes in bundling knowledge of European experts to solve issues where evidence is lacking for rare diseases like gastroschisis. Therefore, the panel advises installing a feeding protocol working group to design an ERNICA feeding protocol. This process may include systematic evaluation and comparison of published feeding protocols for gastroschisis or similar conditions in neonates and the use of formal consensus methodologies like a Delphi process. |
| **REFERENCES SUMMARY** Dama, M., Rao, U., Gollow, I., Bulsara, M., & Rao, S. (2017). Early commencement of enteral feeds in gastroschisis: a systematic review of literature. European Journal of Pediatric Surgery, 27(06), 503-515. Raduma, O. S., Jehangir, S., & Karpelowsky, J. (2021). The effect of standardized feeding protocol on early outcome following gastroschisis repair: A systematic review and meta-analysis. Journal of Pediatric Surgery, 56(10), 1776-1784. Utria, A. F., Wong, M., Faino, A., Jacobson, E., & Javid, P. J. (2022). The role of feeding advancement strategy on length of stay and hospital costs in newborns with gastroschisis. Journal of Pediatric Surgery, 57(3), 356-359. |

| Question 3.2 | |
| --- | --- |
| **What is the optimal placement location for a central line in infants with gastroschisis?** | |
| **Population:** | Infants with gastroschisis |
| **Intervention:** | Upper extremity central line for total parenteral nutrition |
| **Comparison:** | Lower extremity central line for total parenteral nutrition |
| **Main outcomes:** | Complication rates of central line (infiltration, phlebitis) |
| **Setting:** | Modern high resource hospital |

Assessment

| Problem  Is the problem a priority? | | |
| --- | --- | --- |
| Judgement | Research evidence | Additional considerations |
| ○ No ○ Probably no ● Probably yes ○ Yes ○ Varies ○ Don't know | Neonates with gastroschisis often have a prolonged time until full enteral feeding is reached. There is little knowledge on the best location to place a central line, therefore it is often chosen by preference of the caregiver. |  |
| Desirable Effects  How substantial are the desirable anticipated effects? | | |
| Judgement | Research evidence | Additional considerations |
| ○ Trivial  ○ Small ● Moderate ○ Large ○ Varies ○ Don't know | This study suggests that the risk of complications (mainly infiltration and phlebitis) is significantly lower when the line is placed in the upper extremity ( RR 0.17  95% CI 0.05to 0.60) . The complication risks of PICCs generally increase with the length of time the catheter remains in place, therefore Ma et al. (2014) expressed their results in complication rate in the first five days after abdominal closure. If the results are split between primary closure and silo, then the complication rates amongst patients with primary closure do not reach significant differences. In group treated with silo, the overall complication risk was over 9 times higher when a PICC line was placed in the lower extremity (OR 9.7 95 % CI: 1.9 to 49.7). | The higher risk for complications in silo patients can possibly be explained by compression tot the veins in the lower extremities during silo which increases the risk for thrombosis. |
| Undesirable Effects  How substantial are the undesirable anticipated effects? | | |
| Judgement | Research evidence | Additional considerations |
| ○ Large ○ Moderate ○ Small ● Trivial ○ Varies ○ Don't know | No undesirable effects reported. |  |
| Certainty of evidence  What is the overall certainty of the evidence of effects? | | |
| Judgement | Research evidence | Additional considerations |
| ● Very low ○ Low ○ Moderate ○ High ○ No included studies | Although the study corrected for treatment type and time that the catheter was in place, the lack of a multivariate analysis to adjust for confounders could be an issue for reliability of the results. The selection procedure of patients, even if drawn from the same sample, the placement of lines according to the nurse’s preference could have led to selection bias. Therefore, the level of evidence was downgraded for risk of bias to very low. This implies that the true effect may variate substantially from this estimate. |  |
| Values  Is there important uncertainty about or variability in how much people value the main outcomes? | | |
| Judgement | Research evidence | Additional considerations |
| ○ Important uncertainty or variability ○ Possibly important uncertainty or variability ● Probably no important uncertainty or variability ○ No important uncertainty or variability |  |  |
| Balance of effects  Does the balance between desirable and undesirable effects favor the intervention or the comparison? | | |
| Judgement | Research evidence | Additional considerations |
| ○ Favors the comparison ○ Probably favors the comparison ○ Does not favor either the intervention or the comparison ●Probably favors the intervention ○ Favors the intervention ○ Varies ○ Don't know | There is no evidence that upper extremity placement is associated with any particular harms compared with lower extremity, and does show benefits, especially for patients with a silo. Even though the quality of evidence is low, the panel agrees that the potential risk difference is probably in favor of the upper extremity placement. |  |
| Resources required  How large are the resource requirements (costs)? | | |
| Judgement | Research evidence | Additional considerations |
| ○ Large costs ○ Moderate costs ● Negligible costs and savings  ○ Moderate savings ○ Large savings ○ Varies ○ Don't know | The main decrease in complications with an upper extremity line compared to a lower line are from phlebitis and infiltration. These conditions often do not require treatments that lead to large additional costs. |  |
| Certainty of evidence of required resources  What is the certainty of the evidence of resource requirements (costs)? | | |
| Judgement | Research evidence | Additional considerations |
| ○ Very low ○ Low ○ Moderate ○ High ● No included studies |  |  |
| Cost effectiveness  Does the cost-effectiveness of the intervention favor the intervention or the comparison? | | |
| Judgement | Research evidence | Additional considerations |
| ○ Favors the comparison ○ Probably favors the comparison ○ Does not favor either the intervention or the comparison ○ Probably favors the intervention ○ Favors the intervention ○ Varies ● No included studies |  |  |
| Equity  What would be the impact on health equity? | | |
| Judgement | Research evidence | Additional considerations |
| ○ Reduced ○ Probably reduced ○ Probably no impact ● Probably increased ○ Increased ○ Varies ○ Don't know | If complications such as infection are higher with a lower extremity line, suggesting the option to choose an upper extremity line first can cause an increase in equity amongst patients treated in different hospitals as this results in equal estimated risks for infection for all patients. |  |
| Acceptability  Is the intervention acceptable to key stakeholders? | | |
| Judgement | Research evidence | Additional considerations |
| ○ No ○ Probably no ○ Probably yes ● Probably yes ○ Yes ● Varies ○ Don't know |  | For some hospitals, the PICC is not the first option as they would resort to a central venous line. These hospitals would like to see evidence comparing the PICC to the central venous catheter before accepting the recommendation. For this guideline, such evidence was looked for but could not be found.  The tendency within the guideline panel based on their professional opinion is that PICC lines do lead to less infections over central venous catheters and that PICC lines probably are associated with less discomfort for the neonate. |
| Feasibility  Is the intervention feasible to implement? | | |
| Judgement | Research evidence | Additional considerations |
| ○ No ○ Probably no ● Probably yes ○ Yes ○ Varies ○ Don't know |  |  |

Summary of judgements

|  | **Judgement** | | | | | | |
| --- | --- | --- | --- | --- | --- | --- | --- |
| **Problem** | No | Probably no | Probably yes | Yes |  | Varies | Don't know |
| **Desirable Effects** | Trivial | **Small** | Moderate | Large |  | Varies | Don't know |
| **Undesirable Effects** | Large | Moderate | Small | **Trivial** |  | Varies | Don't know |
| **Certainty of evidence** | **Very low** | Low | Moderate | High |  |  | No included studies |
| **Values** | Important uncertainty or variability | Possibly important uncertainty or variability | **Probably no important uncertainty or variability** | No important uncertainty or variability |  |  |  |
| **Balance of effects** | Favors the comparison | Probably favors the comparison | Does not favor either the intervention or the comparison | Probably favors the intervention | Favors the intervention | Varies | Don't know |
| **Resources required** | Large costs | Moderate costs | **Negligible costs and savings** | Moderate savings | Large savings | Varies | Don't know |
| **Certainty of evidence of required resources** | Very low | Low | Moderate | High |  |  | **No included studies** |
| **Cost effectiveness** | Favors the comparison | Probably favors the comparison | Does not favor either the intervention or the comparison | Probably favors the intervention | Favors the intervention | Varies | **No included studies** |
| **Equity** | Reduced | Probably reduced | Probably no impact | **Probably increased** | Increased | Varies | Don't know |
| **Acceptability** | No | Probably no | Probably yes | Yes |  | **Varies** | Don't know |
| **Feasibility** | No | Probably no | **Probably yes** | Yes |  | Varies | Don't know |

Type of recommendation

| Strong recommendation against the intervention | Conditional recommendation against the intervention | Conditional recommendation for either the intervention or the comparison | **Conditional recommendation for the intervention** | Strong recommendation for the intervention |
| --- | --- | --- | --- | --- |
| ○ | ○ | ○ | **●** | ○ |

| Justification |
| --- |
| *Balance of effects*  The upper extremity placement of a central venous catheter is superior compared to the lower extremity by leading to less complications.  If the upper extremity placement fails, placement in the neck can be considered. |

ConclusioNs

| Recommendation |
| --- |
| The panel suggests the upper extremity as first choice in case of placement of a peripherically-insterted central catheter (PICC)   \| Subgroup considerations \| \| --- \| \| The risk difference is larger in patients with a silo. Should lower extremity PICC line be the option of choice in a patient with a silo, cautious surveillance for complications is warranted.   \| Research priorities \| \| --- \| \| For some hospitals, the PICC is not the first option as they would resort to a central venous line. Randomized or prospective studies comparing these types of lines in neonates with gastroschisis could be of value for future development of care. \| \| |
|  |

|  |
| --- |

References Summary

Ma, M., Garingo, A., Jensen, A. R., Bliss, D., & Friedlich, P. (2015). Complication risks associated with lower versus upper extremity peripherally inserted central venous catheters in neonates with gastroschisis. *Journal of pediatric surgery*, *50*(4), 556-558.
