# Supplementary material for: European reference network for rare inherited congenital anomalies (ERNICA) evidence based guideline on the management of gastroschisis
Source: Orphanet J Rare Dis. 2024 Feb 12;19:60. doi: 10.1186/s13023-024-03062-8 (PMC10860293; doi:10.1186/s13023-024-03062-8)
Supplement: Supplementary file 4 — Additional file 4. Appendix D: Survey for patient-parent perspective. [file 13023_2024_3062_MOESM4_ESM.docx]

**Appendix D: Survey for patient-parent perspective**

# Survey for parents of patients with gastroschisis.

## Care before birth

1. Did you get enough information on why it is important to follow up (including ultrasound) pregnancies with gastroschisis?
    Yes
    No
   1b. If no, what did you miss?
   _______________________________________________________________________
   _______________________________________________________________________
2. Did you have the opportunity to discuss the mode of delivery (vaginal birth or cesarean section) of your child with your doctor?
    Yes
    No
3. Did you have the opportunity to discuss the timing of delivery of your child with your doctor?
    Yes
    No

## Closure of the abdominal wall

1. Did you receive any form of counselling before the surgery/treatment about the options for closure of gastroschisis?
    Yes and it was helpful
    Yes but is was not helpful/not enough

No

4a. If counselling was not offered, or not helpful/not enough, what would have been helpful?
_______________________________________________________________________
_______________________________________________________________________

## Feeding

1. Does a specific feeding protocol exist in your hospital?
    Yes
    No

I don’t know / it was not mentioned to me

5a. If yes, was is helpful for you?
 Yes
 No

1. Did you miss any kind of support/information with regard to feeding your child while in the hospital?
    Yes, I missed support or supportive materials
    Yes, I missed information
    Yes, I missed both support/supportive materials and information
    No

## Organization of Care

1. Where did you turn for information on gastroschisis? (multiple answers possible)
    I received information from my hospital, spoken and/or written
    I searched google
    I looked into the ERNICA website
   Other, namely:_____________________________________________________________ ________________________________________________________________________________________________________________________________________________________
    I tried different options but didn’t find the information I was looking for
2. Did your doctor tell you what to expect of the care pathway? For example length of hospital stay, going home with your baby, check-ups?
    Yes
    No
3. Did you miss any kind of support/information with regard to feeding your child after discharge?
    Yes, I missed support or supportive materials
    Yes, I missed information
    Yes, I missed both support/supportive materials and information
    No
4. Did you receive any form of counselling or (psychological) support (before and/or after birth)?
    Yes, and I thought it was helpful
    Yes, but it was not helpful for me
    It was offered to me but I did not use it
    It was not offered to me, but I would have liked it
    It was not offered to me, but I did not miss it.
5. Did you get in touch with other parents with a child with gastroschisis (before and/or after birth)?
    Yes, and I thought it was helpful
    Yes, but it was not helpful for me
    I know about parent support options in my region but I did not turn to it.
    I have never met other parents, but I would have liked it
    I have never met other parents, but I did not miss it.

   11a. If you got in touch with other parents, how did you do so?
   I found other parents going through the same on social media and we got in touch without
    any organization
   Informal online support group such as a (closed) Facebook group.
   A parental support group organized by my hospital
   Support groups or informative events offered by a professional patient advocacy
    organization
   Other, namely:_____________________________________________________________ ________________________________________________________________________________________________________________________________________________________
6. Which parts of care, such as ward visits, information folders, websites or people (nurses, doctors, social workers) were most helpful for you and your child? (during pregnancy or after birth)
   _______________________________________________________________________
   _______________________________________________________________________
   _______________________________________________________________________
7. What would you like to see improved in the care, treatment, diagnosis or counselling of a child with gastroschisis in your country?
   ___________________________________________________________________________
   ___________________________________________________________________________
   ___________________________________________________________________________
